# A super-bulky phosphanide ligand enables the observation of scalar coupling between ^31^P and ^7^Li, ^23^Na, ^85^Rb/^87^Rb, and ^133^Cs†

**DOI:** 10.1039/d6dt02307j

**Published:** 2026-10-21

**Authors:** Ahmed Hadadi, Corinne Y. Wills, Casey M. Dixon, Paul G. Waddell, Keith Izod

**Affiliations:** a Main Group Chemistry Laboratories, School of Chemistry, Newcastle University Newcastle upon Tyne NE1 7RU UK; b Department of Chemistry, Imam Mohammad Ibn Saud Islamic University (IMSIU) Riyadh 11623 Saudi Arabia keith.izod@ncl.ac.uk

## Abstract

Scalar coupling between nuclei provides some of the most useful information available from an NMR spectrum. While scalar coupling between ^31^P and ^6/7^Li may be observed at low temperatures, or in a very small number of cases at room temperature, coupling between ^31^P and the remaining alkali metal nuclei has remained elusive. The super-bulky chlorophosphane (^Me^Bhp)_2_PCl (1Cl) provides convenient access to the alkali metal phosphanides {(^Me^Bhp)_2_P}ML [ML = Li(THF)_2_·1/2PhMe (1Li), Na(OEt_2_) (1Na), K(PhMe)·2.5PhMe (1K·Tol), K(THF)_3_·2PhMe (1K·THF), Rb (1Rb) and Cs(OEt_2_)·Et_2_O (1Cs·Et_2_O)], which crystallise with short M–P distances (except 1K·THF) and trigonal planar phosphorus centres (except 1Na). The structural features of these compounds enable the NMR observation of scalar coupling between heavier alkali metal nuclei and another nucleus (in this case ^31^P) for the first time.

## Abstract 2

A full set of alkali metal complexes of a super-bulky phosphanide ligand was synthesised.  These compounds enable observation by NMR spectroscopy of scalar coupling between ^31^P and heavier alkali metal nuclei for the first time.

## Graphical abstract

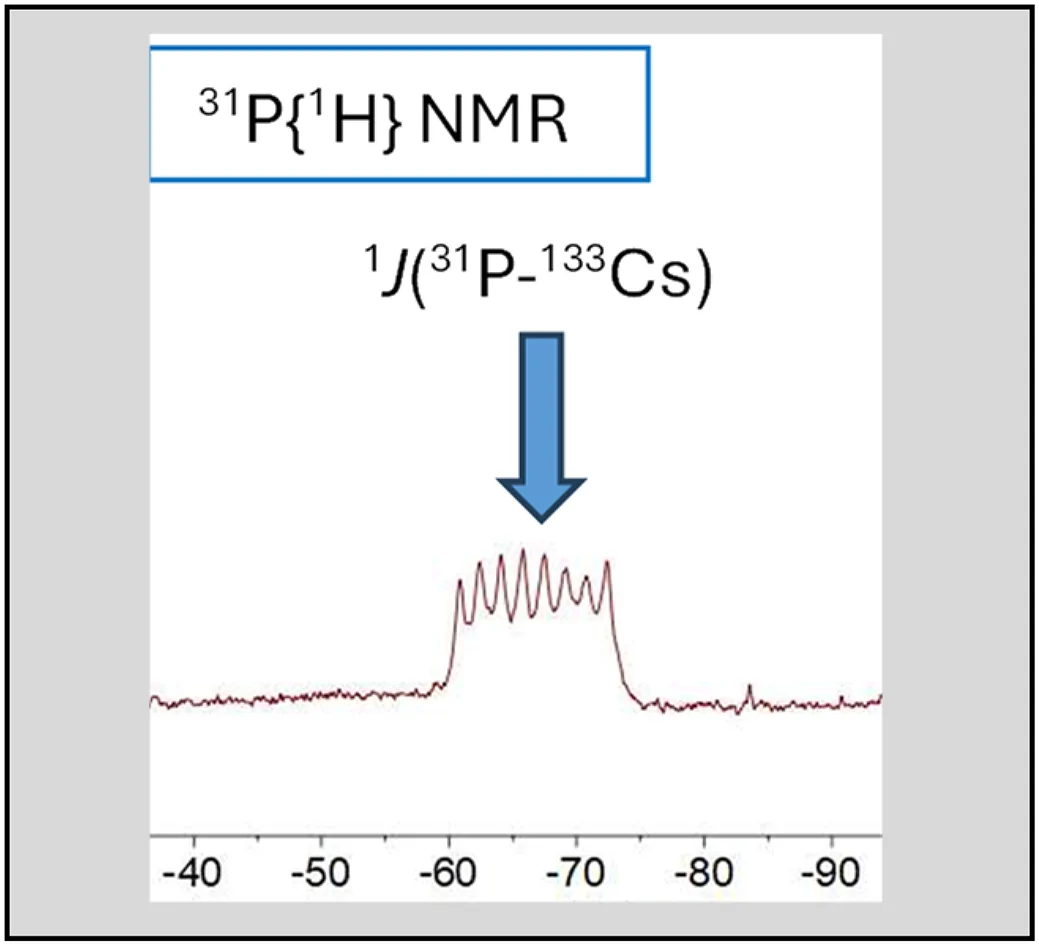

## Introduction

The chemical shift of a nucleus and the magnitude of indirect scalar coupling between nuclei provide some of the most useful information available from an NMR spectrum. Where NMR active nuclei of reasonable natural abundance are covalently bonded, scalar coupling is usually relatively straightforward to observe in solution (in the absence of significant line-broadening, *e.g.* quadrupolar, effects).^1^ However, where bonds between two atoms are more electrostatic in nature, scalar coupling information is usually lost in solution due to rapid intermolecular exchange of the coupled nuclei. It is, therefore, of little surprise that complexes of the highly electropositive alkali metals rarely exhibit scalar coupling between the alkali metal and ligand nuclei at room temperature in solution.

The two naturally occurring nuclei of lithium (^6^Li, ^7^Li) together comprise the total natural abundance of terrestrial lithium and both have amenable NMR properties: ^6^Li *I* = 1, 7.42%, ^7^Li *I* = 3/2, 92.58%.^2^ While both ^6^Li and ^7^Li are quadrupolar, the quadrupole moments of these nuclei are sufficiently small that spectral line shapes are relatively sharp, both for the nuclei themselves and for the adjacent nuclei to which they are coupled. Like complexes of the other alkali metals, lithium complexes are rather labile; however, the high charge: radius ratio of the small Li^+^ cation generally results in stronger complexes, such that at lower temperatures dynamic intermolecular exchange may be slow enough that scalar coupling between *e.g.*^13^C and ^7^Li/^6^Li may be resolved (especially when the material has been isotopically enriched with ^13^C and/or ^6^Li).^3,4^ In a small number of cases, the rate of exchange is sufficiently slow that scalar coupling to ^7^Li/^6^Li is observed at room temperature.^5^

Alkali metal phosphanides R_2_PM (R = *e.g.* alkyl, aryl, silyl, H; M = Li, Na, K, Rb, Cs) are essential starting materials for the synthesis of both organic and inorganic phosphorus-containing materials *via* stoichiometric, *e.g.* salt metathesis, reactions. Recently, Mulvey and co-workers have also shown that alkali metal phosphanides are excellent catalysts for the hydrophosphination of unsaturated organic compounds.^6,7^ In particular, these authors have demonstrated that, for the compounds (Ph_2_P)M(crown) [M = Li, Na, K, Rb, Cs; crown = 15-crown-5 or 18-crown-6] the caesium derivative significantly outperforms the lighter members of the group in catalysing the hydrophosphination of a range of alkenes and alkynes.^7^ In the broader context, this may be viewed as a manifestation of the so-called “caesium effect”, whereby caesium compounds have been shown to be both more soluble and more reactive for a variety of organic transformations than their lighter congeners.^8^ This effect may, at least in part, be attributed to the low charge-to-radius ratio of the large caesium cation, which leads to the formation of separated ion pairs, and thus reactive anions, in polar solvents. In addition to the foregoing, Stephan and co-workers have shown that potassium phosphanides act as Frustrated Lewis pair catalysts for the hydrogenation of alkenes and imines.^9^

In the solid state alkali metal phosphanides adopt a plethora of structural motifs, including monomers, dimers, trimers, cuboidal clusters, ladders, polymers and 3-dimensional lattices.^10–13^ The majority of structurally characterised compounds of this type are lithium derivatives,^12^ as these are the most accessible species (through deprotonation of the corresponding phosphane with *e.g. n*BuLi), and thus the most easily available reagents for onward metathesis reactions. Structural studies of heavier alkali metal phosphanides remain rather scarce, especially for the heaviest elements rubidium and caesium.^6,7,13^

Among alkali metal complexes phosphanides provide perhaps the best opportunity to observe scalar coupling to the alkali metal nucleus, since ^31^P has a nuclear spin of ½ and has 100% natural abundance. However, once again, the majority of NMR studies are of lithium phosphanides. The first example of ^7^Li–^31^P scalar coupling was reported by McFarlane and co-workers in 1982:^14^ at 200 K the ^7^Li and ^31^P{^1^H} NMR spectra of Ph_2_PLi in diethyl ether consist of a binomial triplet and a 1 : 2 : 3 : 4 : 3 : 2 : 1 septet, respectively, confirming the dimeric nature of this compound in solution. Several other NMR studies have demonstrated the existence of monomers, dimers, and trimers in solution and have shown that these oligomers are often in a temperature-dependent equilibrium with each other.^15^ Beyond lithium, to the best of our knowledge, scalar coupling to NMR active nuclei of the heavier group 1 elements has yet to be observed.

Direct *dipolar* coupling to heavier alkali metal nuclei may be observed in the solid state. However, scalar and dipolar coupling are mediated by two distinct mechanisms: scalar coupling is primarily propagated through bonds, whereas dipolar coupling is a through space effect.^16^ In essence scalar coupling provides evidence of bond connectivity whereas dipolar coupling may provide information on internuclear distances. In solution molecular tumbling averages dipolar coupling to zero and, in the solid state under magic angle spinning (MAS) conditions, dipolar coupling is also zeroed.^16^ However, it is possible to observe dipolar coupling under MAS conditions using double-resonance recoupling schemes such as REDOR (Rotational-Echo Double Resonance) and TRAPDOR (Transfer of Populations *via* Double Resonance).^17,18^ These techniques provide unique structural information such as cation-phosphorus internuclear distance distributions and, therefore, medium-range order in, for example, alkali metal phosphate glasses.^17,18^

In this contribution we report the synthesis of a super-bulky phosphanide ligand, which forms low coordinate complexes with the alkali metals, enabling the observation of scalar coupling between ^31^P and the heavier alkali metal nuclei ^23^Na, ^85^Rb/^87^Rb and ^133^Cs for the first time. This represents the first observation of scalar coupling between these heavier alkali metal nuclei and *any other nucleus*.

## Results and discussion

While the primary chlorophosphane (^Me^Bhp)PCl_2_ has been prepared previously,^19^ the significantly more bulky secondary chlorophosphane (^Me^Bhp)_2_PCl has not been reported [^Me^Bhp = 2,6-(Ph_2_CH)_2_-4-MeC_6_H_2_]. Unexpectedly, we found that the reaction between two equivalents of (^Me^Bhp)Li and PCl_3_ in toluene at −40 °C gave the chlorophosphane (^Me^Bhp)_2_PCl (1Cl) as a colourless crystalline solid in good (72%) yield (Scheme 1), in spite of the steric bulk of the ^Me^Bhp substituents. It proved essential to maintain the temperature below −40 °C during the course of this reaction to prevent isomerisation of (^Me^Bhp)Li to the more thermally stable benzylic derivative 1-(Ph_2_CH)-3-Me-5-{Ph_2_C(Li)}C_6_H_2_.

**Scheme 1:**
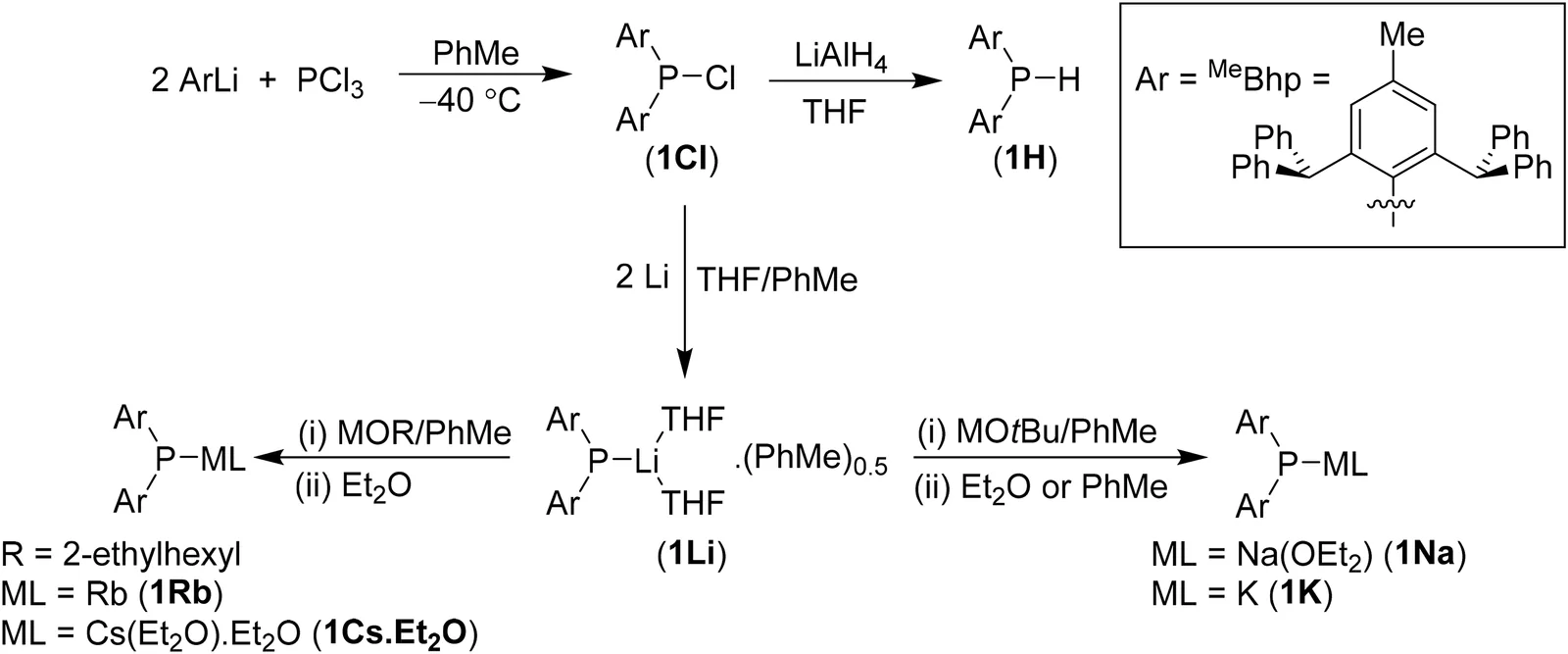
Synthesis of 1Cl, 1H and 1M.

Treatment of 1Cl with LiAlH_4_, followed by an aqueous work-up with degassed water gave the secondary phosphane (^Me^Bhp)_2_PH (1H) as a colourless solid in good (66%) yield. Compound 1H was crystallised as four distinct solvates, (^Me^Bhp)_2_PH, (^Me^Bhp)_2_PH·4PhMe, (^Me^Bhp)_2_PH·4PhH, and (^Me^Bhp)_2_PH·2½PhMe·THF (see the SI), although the latter three solvates desolvate rapidly on exposure to vacuum to give the solvent-free form. The ready synthesis of 1H contrasts with the often fraught syntheses of severely sterically hindered diarylphosphanes. For example, Killian and co-workers reported that reduction of super-bulky Mes*_2_P(O)Cl (2) does not yield the corresponding secondary phosphane Mes*_2_PH, under a variety of forcing conditions and employing a range of hydride-transfer reagents (Mes* = 2,4,6-*t*Bu_3_C_6_H_2_).^20^ The more straightforward isolation of 1H is reminiscent of the ready syntheses of the significantly less hindered (Dipp)_2_PH^21^ and (Trip)_2_PH^22^ and demonstrates the less sterically demanding nature of the benzhydryl groups in 1H compared to the *t*Bu-groups in Mes* derivatives, since the former can rotate such that the phenyl rings are directed away from the phosphorus atom, thereby reducing the steric hindrance at this centre (Dipp = 2,6-*i*Pr_2_C_6_H_3_; Trip = 2,4,6-*i*Pr_3_C_6_H_2_).

While 1H is readily accessible, we find that deprotonation of this compound to give the corresponding alkali metal phosphanides is non-trivial, probably due to the very sterically occluded nature of the P–H hydrogen atom and/or competing deprotonation of the benzhydryl substituents to give trityl-like carbanions. Thus, the reaction between 1H and *n*BuLi under a variety of conditions, including reaction at elevated temperatures for extended periods in a variety of solvents, did not yield the corresponding lithium complex {(^Me^Bhp)_2_P}Li. Nevertheless, the reaction between 1H and an excess of PhCH_2_K in THF under reflux for three hours led to complete deprotonation, according to ^31^P{^1^H} NMR spectroscopy, to give the potassium complex {(^Me^Bhp)_2_P}K (1K), although this could be isolated in only moderate yield and purity; compound 1K is more easily accessed *via* the corresponding lithium derivative (see below).

The lithium phosphanide {(^Me^Bhp)_2_P}Li(THF)_2_·1/2PhMe (1Li) was successfully synthesised from the reaction between 1Cl and two equivalents of elemental lithium in THF (Scheme 1). Sonication of this mixture for one hour, followed by stirring at room temperature for three days, led to complete consumption of 1Cl to give 1Li in excellent (86%) yield, after removal of the LiCl side-product and crystallisation from cold toluene, as a crystalline red solid.

Treatment of 1Li with either NaO*t*Bu or KO*t*Bu in toluene, followed by crystallisation from diethyl ether gave the corresponding heavier alkali metal complexes {(^Me^Bhp)_2_P}Na(OEt_2_) (1Na) and {(^Me^Bhp)_2_P}K (1K), respectively. Crystallisation of 1K from toluene gave single crystals of polymeric [{(^Me^Bhp)_2_P}K(PhMe)·(PhMe)_2.5_]_∞_ (1K·Tol), whereas crystallisation from a THF/toluene mixture gave single crystals of the alternative monomeric solvate {(^Me^Bhp)_2_P}K(THF)_3_·2PhMe (1K·THF). Similarly, the reactions between 1Li and either RbOR or CsOR (R = CH_2_CH(Et)(CH_2_)_3_Me) in toluene gave the complexes [{(^Me^Bhp)_2_P}Rb]_∞_ (1Rb) and {(^Me^Bhp)_2_P}Cs(PhMe) (1Cs·Tol), respectively, as dark red powders. Single crystals of solvent-free 1Rb were obtained by slow evaporation of a diethyl ether solution at room temperature, whereas single crystals of the alternative solvate of 1Cs, [{(^Me^Bhp)_2_P}Cs(OEt_2_)·Et_2_O]_∞_ (1Cs·Et_2_O), were obtained from cold diethyl ether. Compounds 1M are very sensitive to oxygen and moisture, but are stable in the solid state and in solution in the absence of air for several weeks.

Compounds 1Li, 1Na, and 1K·THF adopt monomeric structures in the solid state, whereas compounds 1K·Tol, 1Rb, and 1Cs·Et_2_O are polymeric (Fig. 1). The solid state structures of each compound exhibit short M–P distances and multihapto interactions between the alkali metal centres and one or more of the benzhydryl phenyl rings. The lithium ion in 1Li has an η^1^-Ph contact to one of the phenyl rings of a benzhydryl group of the ligand, in addition to coordination by the phosphorus centre and two molecules of THF, to give a pseudo-tetrahedral geometry about the lithium ion; there is an additional half a molecule of toluene in the asymmetric unit. In 1Na the sodium ion has an η^2^-Ph interaction with one of the benzhydryl groups, in addition to coordination by the phosphorus centre and one molecule of diethyl ether. The potassium ion in monomeric 1K·THF is coordinated by the phosphorus centre and by three molecules of THF and has one η^3^- and one η^6^-Ph interaction with a phenyl ring in separate benzhydryl groups of the ligand. In contrast, the potassium ion in polymeric 1K·Tol has a short contact to the phosphorus centre, and one η^3^- and one η^5^-Ph interaction with a phenyl ring in separate benzhydryl groups of the ligand; the coordination of this ion is completed by an η^2^-interaction with a molecule of toluene and one η^1^- and one η^2^-Ph interactions with a single benzhydryl group of a second {(^Me^Bhp)_2_P}K unit, creating a polymeric chain. The rubidium ion in 1Rb is solvent free and is coordinated by the phosphorus centre, one η^3^- and one η^5^-Ph interaction with phenyl rings in separate benzhydryl groups of the ligand; these units are linked into a sheet polymer *via* one η^1^- and one η^2^-Ph interaction to one benzhydryl group on an adjacent {(^Me^Bhp)_2_P}Rb unit and one η^1^-Ph interaction to a third {(^Me^Bhp)_2_P}Rb unit. Finally, the caesium ion in 1Cs·Et_2_O is coordinated by the phosphorus centre and one molecule of diethyl ether; the coordination of the caesium ion is completed by η^6^-Ph interactions with two benzhydryl groups of the phosphanide ligand and η^2^-Ph interactions to two phenyl rings in a single benzhydryl group of an adjacent {(^Me^Bhp)_2_P}Cs unit, generating a polymeric chain.

**Fig. 1:**
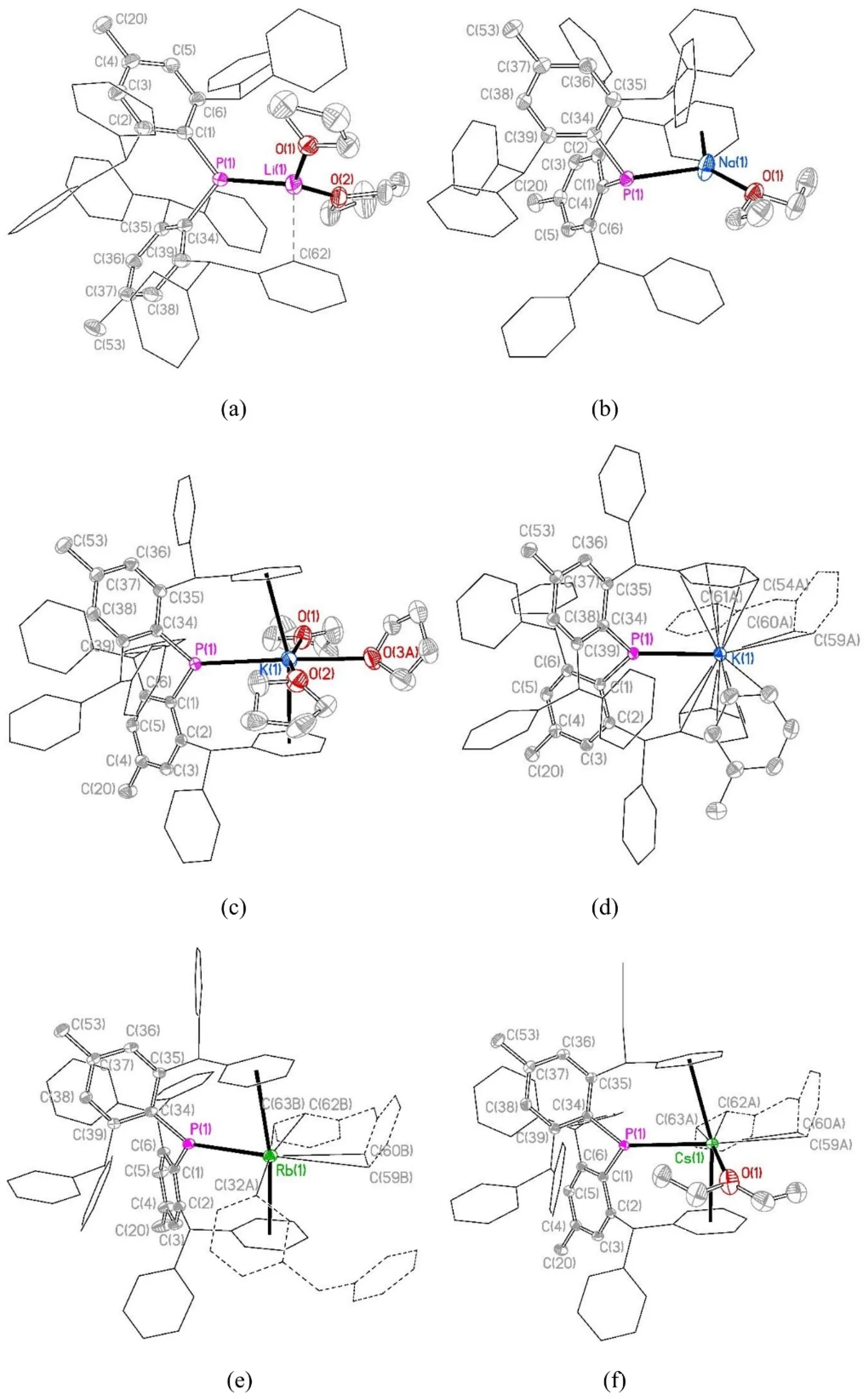
Solid-state structures of (a) 1Li, (b) 1Na, (c) 1K·THF, (d) 1K·Tol, (e) 1Rb, and (f) 1Cs·Et_2_O. Probability ellipsoids are set at 40% and benzhydryl substituents are shown in wireframe to reduce visual clutter; disorder components, hydrogen atoms, and solvent of crystallization are omitted for clarity; partial ligands shown with dotted lines are included for completion of coordination around the metal.

With the single exception of 1Na, which has a distinctly pyramidal phosphorus atom [sum of angles at P = 319.17(9)°], in each case the sum of angles around the phosphorus centres approaches 360°, indicating a planar geometry at phosphorus in the solid state [sum of angles at P = 358.31(13) (1Li), 359.92(8) (1K·Tol), 360.01(8) (1K·THF), 360.00(12) (1Rb), 359.69(11)° (1Cs·Et_2_O)]. With the exception of 1K·THF (K–P distance 3.3089(5) Å), the M–P distances lie at the shorter end of previously reported M–P distances in alkali metal phosphanides [Li–P 2.421(3), Na–P 2.8127(9), K–P 3.0947(4) (1K·Tol), Rb–P 3.2384(5), Cs–P 3.4379(4) Å].^6,7,10–13,15^ This bond shortening may be attributed, at least in part, to (i) the planarity, and hence formal sp^2^ hybridization, of the phosphorus centres (with the exception of 1Na, see above), and (ii) the low formal coordination numbers of the metal centres in 1M. However, we note that the M–P distances in 1M are also short in comparison to previously reported alkali metal phosphanides possessing a planar phosphorus centre.

In order to contextualise the steric demand of our new phosphanide ligand we have calculated the percent buried volume (%*V*_bur_) for Ph_2_P^−^, Mes_2_P^−^, and (^Me^Bhp)_2_P^−^ for comparison (Mes = 2,4,6-Me_3_C_6_H_2_).^23^ Using the X-ray crystallographic coordinates for the monomeric complexes (Ph_2_P)Li(PMDETA),^12*c*^ (Mes_2_P)Li(THF)_3_,^15^ and 1Li, we obtained values of %*V*_bur_ of 24.9, 26.7, and 45.5, respectively, for the phosphanide ligands in each case, clearly demonstrating the much greater steric profile of the (^Me^Bhp)_2_P^−^ ligand. Using the same protocol for the complete series of our compounds, we obtain %*V*_bur_ values of 45.5, 61.6, 48.7, 48.7, and 46.9 for the phosphanide ligands in 1Li, 1Na, 1K·Tol, 1Rb, and 1Cs·Et_2_O, respectively. The pyramidal geometry exhibited by the phosphorus centre in 1Na (see above) may account for the significantly increased %*V*_bur_ value calculated for the phosphanide ligand in this complex, in comparison to those calculated for the remaining compounds, all of which possess near planar phosphorus centres in the solid state. The large %*V*_bur_ values for the (^Me^Bhp)_2_P ligands in 1M strongly suggest that aggregation in solution *via* bridging phosphanide ligands to give dimers or higher aggregates is unlikely. In support of this, we note that, where aggregation is observed in the solid state (1K·Tol, 1Rb, 1Cs·Et_2_O), this is propagated by M⋯Ph interactions, rather than M–P–M contacts.

In solution, the ^31^P NMR signals of compounds 1M exhibit a significant downfield shift with increasing atomic number of the group 1 ion: the ^31^P chemical shifts are −101.6, −100.3, −88.4, −81.7, and −62.6 ppm for 1Li, 1Na, 1K·Tol, 1Rb, and 1Cs·Et_2_O, respectively, a total change in chemical shift of 39.0 ppm. Although caution is warranted in the interpretation of these data, due to the different solvents used in obtaining the relevant spectra and, in some cases, the different co-ligands, the overall trend is clear. NMR investigations of similarly complete series of group 1 phosphanides are rare; however, Mulvey and co-workers have recently reported the ^31^P{^1^H} NMR spectra of a complete series of alkali metal derivatives of the diphenylphosphanide ligand as their crown ether complexes.^13*h*^ These authors note a similar, although less pronounced, downfield shift in the ^31^P resonance from −17.4 ppm for (Ph_2_P)Li(15-crown-5) to +1.5 ppm for (Ph_2_P)Cs(18-crown-6) (a total change in shift of 18.9 ppm, all measured in C_6_D_6_); the trend in ^31^P chemical shifts for these compounds was not commented on in detail. Both of these sets of data indicate a significant deshielding of the ^31^P nucleus with increasing atomic number of the alkali metal cation.

A similar downfield shift is observed in the solid-state ^31^P{^1^H} CP-MAS NMR spectra of 1M (the ^31^P chemical shifts are −107.0, −70.7, −87.2, −77.0, and −64.2 ppm for 1Li, 1Na, 1K·Tol, 1Rb, and 1Cs·Et_2_O, respectively, see Table 1). It is notable that the ^31^P chemical shift of 1Na measured in the solid state is significantly downfield of the signal observed for the *d*_8_-THF solution (−70.7 *vs.* −100.3 ppm, respectively). For the remainder of the series (1Li, 1K·Tol, 1Rb, and 1Cs·Et_2_O) the solution- and solid-state ^31^P chemical shifts differ little and so it is reasonable to assume that, for these compounds, the planar phosphorus centres observed in the solid state are preserved in solution. In contrast, in the solid state the phosphorus centre of 1Na adopts a pyramidal geometry. In THF solution it is likely that the coordination of the sodium ion will differ and so the phosphorus centre may adopt a planar geometry similar to that observed for the other compounds in the series. Such a change in geometry would explain the distinct difference in ^31^P chemical shift of 1Na between the solid and solution states and the similarity of the solution state shift to that expected from the general trend in ^31^P chemical shift of the remaining congeners.

**Table 1:** Selected NMR data for 1Li, 1Na, 1K·Tol, 1Rb, and 1Cs·Et_2_O (spectra were recorded at 298 K unless otherwise stated)

Compound	Solvent	*δ*_P_/ppm	*J*(^31^P–M)/Hz	*K*(^31^P–M)^a^	*δ*_M_/ppm
Ar_2_PLi(THF)_2_·1/2PhMe (1Li)	C_6_D_6_	−101.6	115.7	—	−0.9
	SSNMR^b^	−107.0	120.4	63.6	0.6
Ar_2_PNa(OEt_2_) (1Na)	*D*_8_-THF	−100.3	—	—	—
	SSNMR	−70.7	199.4	154.8	−21.8
[Ar_2_PK(PhMe)·2.5PhMe]_∞_ (1K·Tol)	*D*_8_-THF	−88.4	—	—	—
	SSNMR	−87.2	—	—	—
[Ar_2_PRb]_∞_ (1Rb)	*D*_8_-THF	−81.7	—	—	—
	SSNMR	−77.0	632 (^87^Rb)^c^	395.6	—
			186 (^85^Rb)	394.5	
[Ar_2_PCs(OEt_2_)·Et_2_O]_∞_ (1Cs·Et_2_O)	C_6_D_6_	−62.6	—	—	−50.1
	C_7_D_8_^d^	−66.5	321.3	—	−68.8
	SSNMR	−64.2	422.5	657.8	0.95

a 10^19^ T^2^ J^−1^.

b SSNMR: data from (CP-)MAS solid-state NMR experiments.

c Values obtained from simulated spectrum (see text), *J*(^85^Rb–^31^P) calculated from *J*(^87^Rb–^31^P) using the ratio of magnetogyric ratios of the two nuclei.

d Measured at 233 K.

In order to probe this dichotomy further we have carried out a brief DFT study (see the SI). Minimum energy geometries were located for configurations of 1Na, with identical coordination to that observed in the solid-state structure (*i.e.* with one coordinated molecule of diethyl ether), with either a pyramidal (1Na_pyr_) or a planar phosphorus centre (1Na_plan_). The former of these was calculated to be more stable than the latter by 7.7 kcal mol^−1^, consistent with the observation of a pyramidal phosphorus centre in 1Na in the solid state. However, the degree of solvation of the sodium ion is likely to be different in THF solution compared to the solid state, given that THF is a less bulky ligand and a stronger donor than diethyl ether. This is demonstrated by the coordination of *two* molecules of THF to the smaller lithium ion in 1Li. With a similar coordination environment at the sodium ion, *i.e.* with two coordinated THF ligands per sodium ion, we were able to locate a single minimum energy geometry for this species (1Na·THF_plan_), in which the phosphorus centre is essentially planar (sum of angles at P = 359.93°); all attempts to locate a geometry with a pyramidal phosphorus centre for 1Na·THF resulted in convergence to 1Na·THF_plan_. These DFT studies support our conjecture that, while 1Na adopts a structure in which the phosphorus centre is pyramidal in the solid state, in THF solution the phosphorus centre adopts a planar geometry; the greater steric hindrance induced by the second THF ligand essentially forces the phosphorus centre to adopt a planar configuration, which occupies less of the coordination sphere of the sodium ion (see the %*V*_bur_ calculations above).

At first sight, the increasingly downfield chemical shifts observed for the ^31^P nuclei in compounds 1M appear at odds with the increasingly ionic character of the M–P bond on descending the group from 1Li to 1Cs·Et_2_O, since this should lead to increased electron density at phosphorus and hence increased shielding of the ^31^P nucleus (and a consequent *upfield* change in chemical shift). However, it has been shown that for compounds of the heavier elements, significant contributions to the chemical shift may arise from both scalar and spin–orbit relativistic effects (*i.e.* spin–orbit heavy-atom-on-light-atom (SO-HALA) effects), the latter of which has the greatest influence.^24^ The impact of SO-HALA effects has been little studied for complexes of the alkali metal cations and examples of complete series of alkali metal systems where such effects may be observed are scarce. For the alkali metals, which possess an empty valence shell, the SO-HALA effect is calculated to be *deshielding* and to increase in magnitude with increasing atomic number of the heavy atom, so that the effect should be particularly dominant for the heaviest elements Rb and Cs.^24^ Since the SO-HALA effect is mediated by a Fermi contact-type mechanism, the magnitude of the SO-HALA effect is also related to the hybridization of the light atom, increasing in magnitude with increasing s-character of the light atom contribution to the HA–LA bond. Thus, for ^13^C SO-HALA effects increase as the hybridization of the carbon atom changes from sp^3^ to sp^2^ to sp. A similar situation pertains to the phosphorus nuclei in 1M. Compounds 1M exhibit a planar sp^2^-hybridised phosphorus centre and short internuclear M–P separations and their ^31^P chemical shifts show a substantial downfield shift with increasing atomic number of the alkali metal (a difference of 39.0 ppm from 1Li to 1Cs·Et_2_O compared to a difference of 18.9 ppm from (Ph_2_P)Li(15-crown-5) to (Ph_2_P)Cs(18-crown-6)).^13*h*^ Thus, the foregoing appears to be consistent with a significant SO-HALA contribution to the ^31^P chemical shifts of 1M, leading to increasingly downfield chemical shifts with increasing atomic number of the alkali metal.

Unusually, 1Li exhibits well-resolved ^1^*J*(^31^P–^7^Li) coupling in both its ^31^P{^1^H} and ^7^Li NMR spectra in *d*_8_-toluene solution (^1^*J*_PLi_ = 115.7 Hz in C_6_D_6_), indicating that exchange of the lithium cations is slow on the NMR time-scale at room temperature in this solvent (Fig. 2). The ^31^P–^7^Li coupling constant observed for 1Li is, to our knowledge, the second largest such coupling constant to be reported; for lithium phosphanides typical ^31^P–^7^Li coupling constants lie in the range 35–60 Hz.^10^ The largest ^31^P–^7^Li coupling constant reported previously (*J*_PLi_ = 122 Hz) was recorded for the very sterically hindered, presumably monomeric [{(Me_3_Si)_2_CH}_2_P]Li(TMEDA),^11^ although this compound was not structurally characterised.

**Fig. 2:**
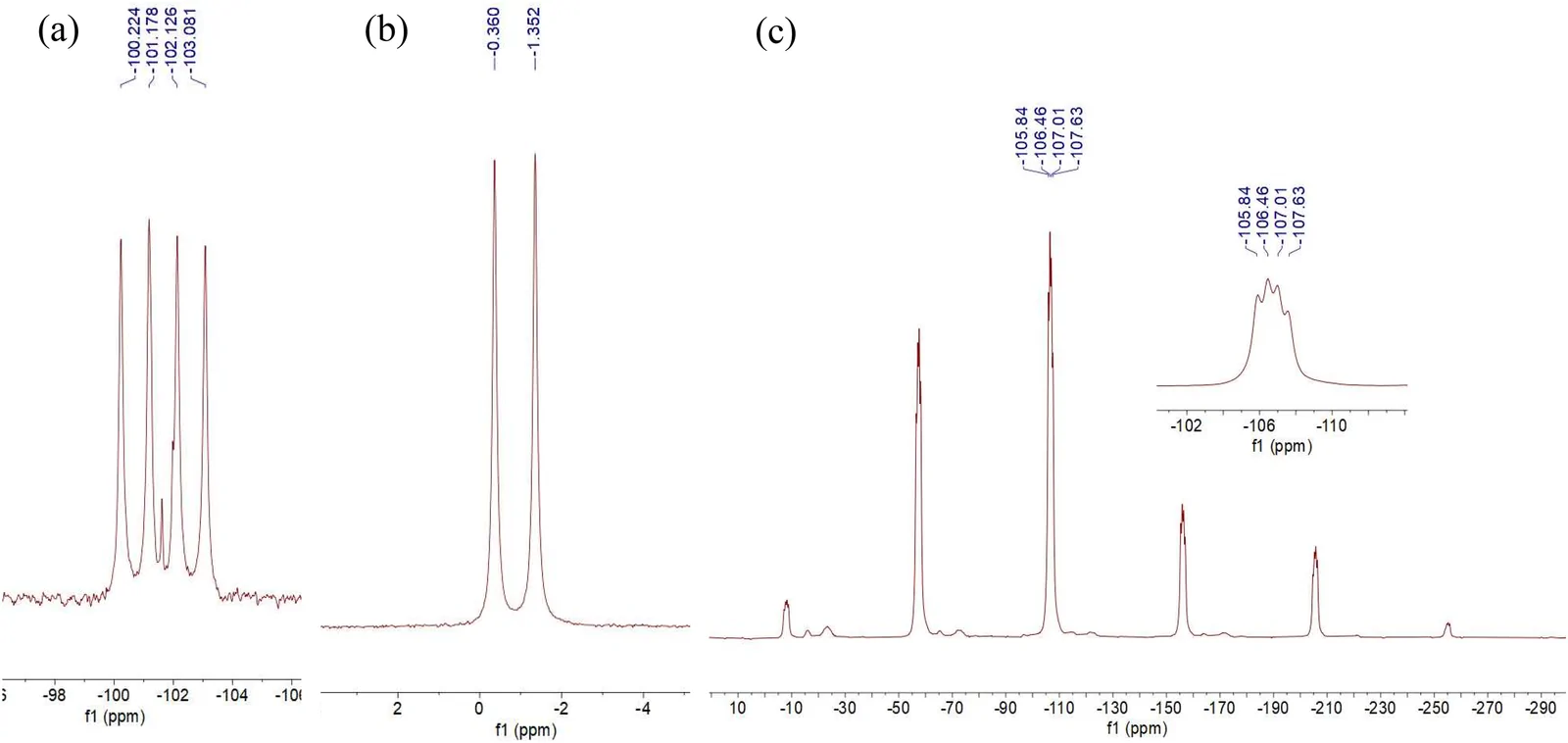
(a) ^31^P{^1^H} and (b) ^7^Li NMR spectra of 1Li at 298 K in *d*_8_-toluene; (c) ^31^P{^1^H} CP-MAS solid-state NMR spectrum of 1Li (spin rate 10 kHz).

The ^31^P{^1^H} Cross-Polarization Magic Angle Spinning (CP-MAS) NMR spectrum of 1Li also exhibits well-resolved ^31^P–^7^Li coupling (*δ*_P_ −107.0 ppm, ^1^*J*_PLi_ = 120.4 Hz), although this coupling is not resolved in the corresponding ^7^Li MAS NMR spectrum, which exhibits a broad singlet due to significant quadrupolar broadening at 0.6 ppm (the quadrupole moment of ^7^Li is −4 × 10^−30^ m^2^).^2^ The close correspondence between the solution-state and solid-state MAS ^31^P{^1^H} and ^7^Li NMR spectra of 1Li suggests that the solid-state structure observed by X-ray crystallography is preserved in solution.

The magnitude of the M–P coupling constant is related through the Fermi contact interaction to the degree of s-character in the M–P bond. In 1Li the degree of s-character in the Li–P bond is enhanced by (i) the largely ionic character of the Li–P bond, and (ii) the planar sp^2^-hybridised nature of the phosphorus centre. These two factors combine to give the very large ^31^P–^7^Li coupling constant observed for 1Li.

Given (i) the large ^31^P–^7^Li coupling constant observed for 1Li, (ii) the fact that compounds 1Li–1Cs·Et_2_O possess very short M–P distances and, (iii) that, with the exception of 1Na, compounds 1Li–1Cs·Et_2_O possess near-planar phosphorus centres in the solid state, we postulated that these compounds would provide the ideal conditions under which to observe coupling between ^31^P and a heavier alkali metal nucleus for the first time.

Although quadrupolar (^133^Cs *I* = 7/2, natural abundance 100%), the quadrupole moment of ^133^Cs is sufficiently small (*Q* = −3 × 10^−31^ m^2^) that line-broadening in non-spherically symmetric systems is often reasonably minor and so we considered that 1Cs·Et_2_O would provide the best opportunity among our compounds for observing ^31^P–^*n*^M coupling for the heavier alkali metals. We first investigated the solid-state ^31^P{^1^H} CP-MAS NMR spectrum of 1Cs·Et_2_O, which exhibits an extremely well resolved equal intensity octet centred at −64.2 ppm (^1^*J*_PCs_ = 422.5 Hz) with relatively few spinning side bands, indicating a relatively low chemical shift anisotropy (Fig. 3(a)); the corresponding ^133^Cs MAS NMR spectrum of 1Cs·Et_2_O consists of a well-resolved doublet at 0.95 ppm (Fig. 3(b)).

**Fig. 3:**
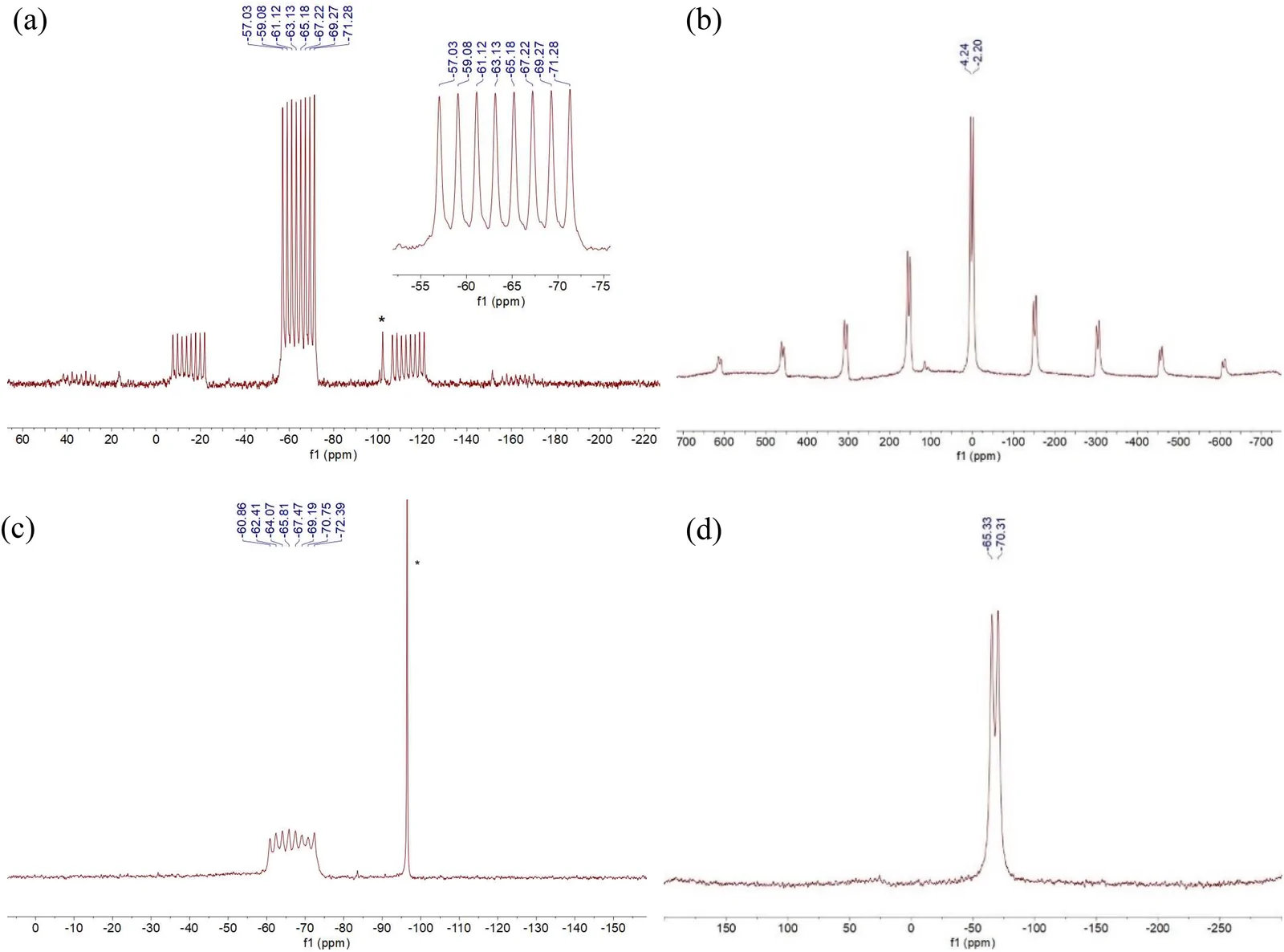
(a) ^31^P{^1^H} CP-MAS solid-state NMR spectrum of 1Cs·Et_2_O (spin rate 10 kHz); (b) ^133^Cs MAS solid-state NMR spectrum of 1Cs·Et_2_O (spin rate 10 kHz); (c) ^31^P{^1^H} and (d) ^133^Cs NMR spectra of 1Cs·Et_2_O in *d*_8_-toluene at 233 K. *Residual (^Me^Bhp)_2_PH due to sample hydrolysis.

The success in observing ^31^P–^133^Cs scalar coupling in the solid state prompted us to investigate the ^31^P{^1^H} and ^133^Cs NMR spectra of 1Cs·Et_2_O in solution. At room temperature in *d*_8_-toluene, the ^31^P{^1^H} and ^133^Cs NMR spectra of 1Cs·Et_2_O consist of broad singlets at −62.6 and −50.1 ppm, respectively. As the temperature is reduced these signals sharpen, eventually resolving at 233 K into an equal intensity octet and a well-resolved doublet (^1^*J*_PCs_ = 321.3 Hz) at −66.5 and −68.8 ppm, respectively (Fig. 3(c) and (d)). Below this temperature the signals broaden again and coupling information is lost. This low-temperature broadening is typical for quadrupolar systems and is connected to the reduced rate of tumbling of the molecules at low temperatures.^2^ The coupling pattern clearly indicates that in solution each phosphorus centre is bonded to a single caesium ion, suggesting that the structure adopted in solution resembles the repeat units of the polymer observed in the solid state.

The above represents the first reported observation of ^31^P–^133^Cs scalar coupling and, to the best of our knowledge, the first observation by NMR spectroscopy of scalar coupling between ^133^Cs and any other nucleus. The absence of previous observations of ^31^P–^133^Cs coupling is a consequence of the largely ionic bonding in caesium complexes and the large size of the Cs^+^ cation, which together result in extremely labile complexes which undergo rapid intermolecular exchange in solution (see above). In 1Cs·Et_2_O exchange is rapid at room temperature, but slows sufficiently by 233 K for coupling to be observed.

The significant difference in ^133^Cs chemical shift between the solid and solution states is indicative of a change in coordination environment around the Cs centre. However, we note that ^133^Cs chemical shifts are extremely sensitive to chemical environment and that relatively small changes in coordination can result in large differences in shift.^25^ For example, the ^133^Cs chemical shifts of Cs_2_CrO_4_ determined by ^133^Cs MAS NMR spectroscopy are −321 and −195 ppm for the two similar, but crystallographically distinct, Cs sites, which are surrounded by ten and nine O atoms, respectively.^26^ In accord with this, we note that, while the ^133^Cs chemical shift of 1Cs·Et_2_O differs by 69.8 ppm between the solution and solid states at room temperature, the ^31^P chemical shifts differ by just 1.6 ppm, suggesting that any change in coordination environment or gross structure between the solid and solution states is minor.

Encouraged by the ready observation of ^31^P–^133^Cs coupling in 1Cs·Et_2_O we were eager to test whether similar ^31^P–^*n*^M coupling could be observed for the remaining complexes 1Na, 1K·Tol, and 1Rb. Although 1Na is monomeric in the solid state it has limited solubility in aromatic/non-polar solvents. Because of this, we were unable to obtain variable-temperature ^31^P{^1^H} NMR spectra of 1Na in *d*_8_-toluene; the solution-state ^31^P{^1^H} NMR spectrum of 1Na in *d*_8_-THF exhibits a sharp singlet at −100.3 ppm, due to fast intermolecular exchange, and this signal does not change appreciably with temperature. However, the ^31^P{^1^H} CP-MAS NMR spectrum of 1Na consists of a fairly well-resolved, equal intensity quartet at −70.7 ppm (Fig. 4; ^1^*J*_PNa_ = 199.4 Hz, ^23^Na *I* = 3/2, 100%). Coupling is not resolved in the corresponding ^23^Na MAS NMR spectrum, which exhibits a broad singlet at −21.8 ppm, due to the very large quadrupole moment of ^23^Na (*Q* = 1.0 × 10^29^ m^2^).

**Fig. 4:**
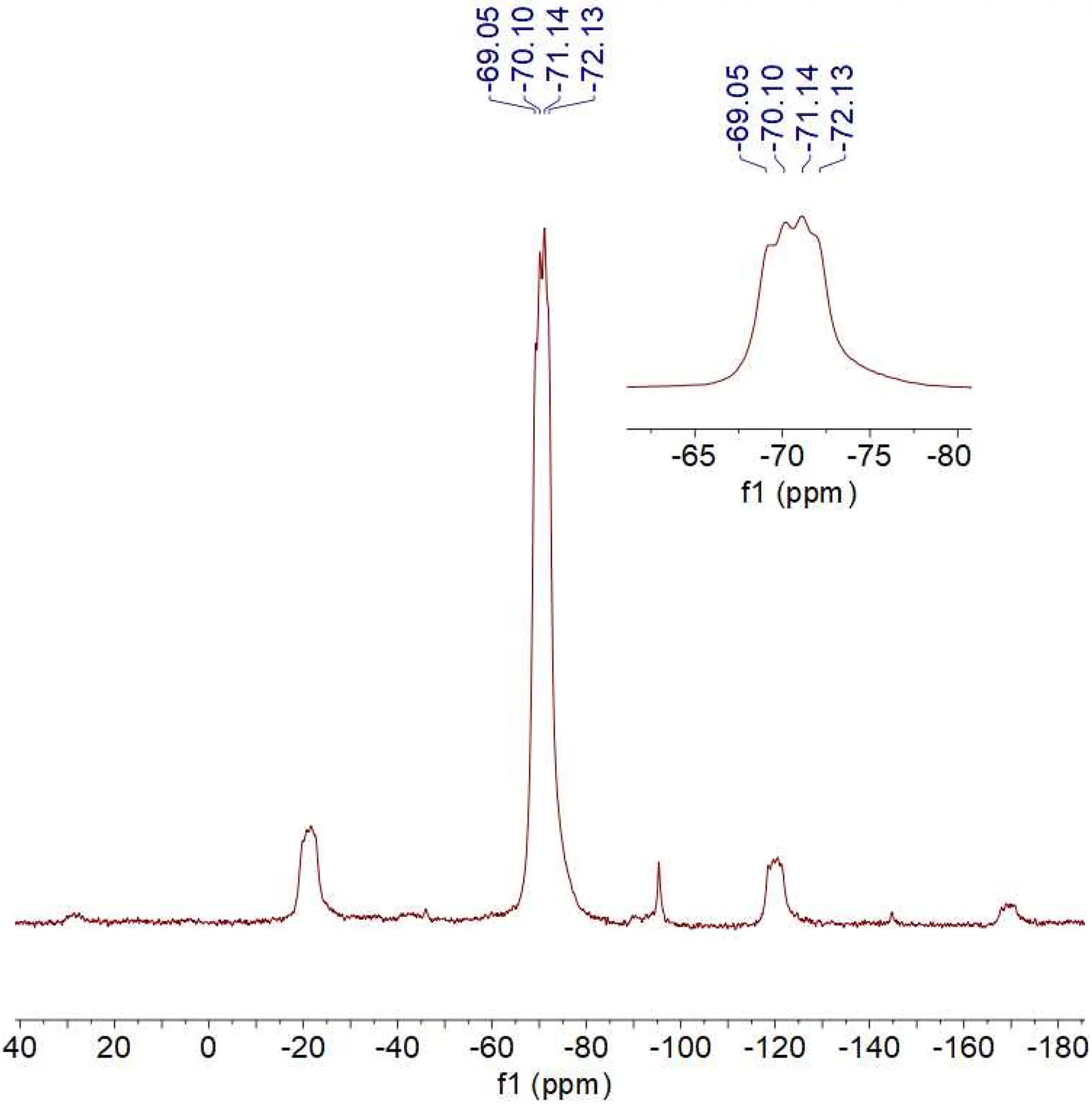
^31^P{^1^H} CP-MAS NMR spectrum of 1Na (spin rate 10 kHz).

In contrast to monomeric 1Na, in the solid state compounds 1K·Tol and 1Rb adopt polymeric chain and sheet-like structures, respectively, and both compounds exhibit very poor solubility in *d*_8_-toluene, especially at low temperature, again preventing detailed solution state studies of these compounds in non-coordinating solvents (where M–P contacts might be preserved). Again, while both 1K·Tol and 1Rb are soluble in THF, this solvent promotes rapid intermolecular M–P exchange and so leads to loss of coupling information.

Rubidium has two NMR active nuclei: ^85^Rb (*I* = 5/2, 72.15%) and ^87^Rb (*I* = 3/2, 27.85%), both of which have a large quadrupole moment (^85^Rb *Q* = 2.6 × 10^29^ m^2^; ^87^Rb *Q* = 1.3 × 10^29^ m^2^). Despite the large quadrupole moments of ^85^Rb and ^87^Rb the ^31^P{^1^H} CP-MAS NMR spectrum of 1Rb consists of a broad multiplet at −77.0 ppm (Fig. 5(a)), clearly indicating coupling between ^31^P and the two Rb nuclei. Indeed, this spectrum was successfully simulated (Fig. 5(b)) to obtain the coupling constants ^1^*J*(^31^P–^85^Rb) = 186 Hz and ^1^*J*(^31^P–^87^Rb) = 632 Hz. Unfortunately, we were unable to locate a ^87^Rb MAS NMR signal, most likely due to extensive quadrupolar broadening.

**Fig. 5:**
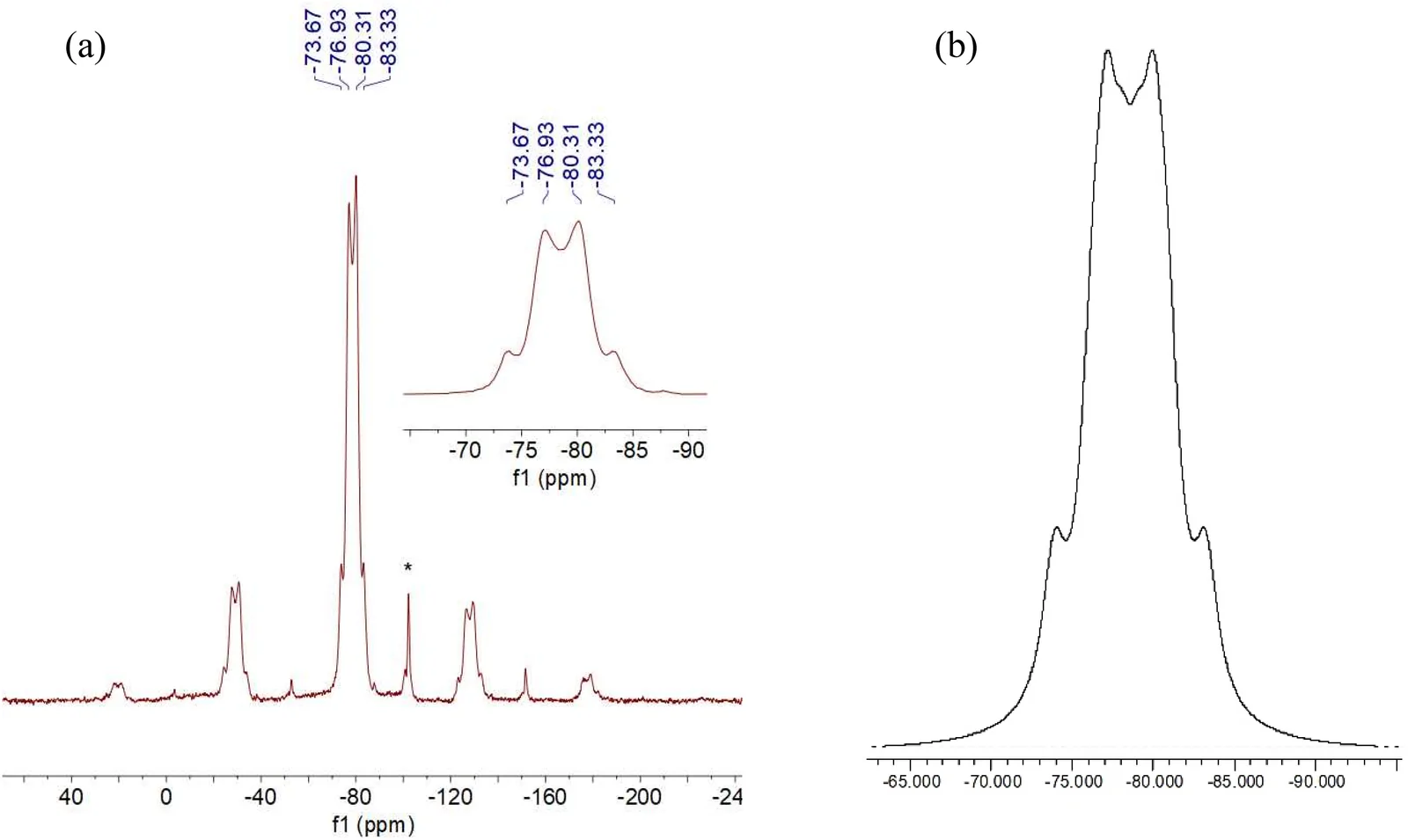
(a) ^31^P{^1^H} CP-MAS NMR spectrum of 1Rb; (b) simulated ^31^P{^1^H} NMR spectrum of 1Rb with ^1^*J*(^31^P–^85^Rb) = 186 Hz, ^1^*J*(^31^P–^87^Rb) = 632 Hz and line width = 350 Hz.

Potassium has a single naturally abundant NMR active nucleus (^39^K *I* = 3/2, 100%) which has a quadrupole moment of similar magnitude to that of ^7^Li (^7^Li *Q* = −4 × 10^−30^, ^39^K *Q* = 4.9 × 10^−30^ m^2^). It might therefore be expected that quadrupolar broadening of the ^31^P nuclei due to ^7^Li and ^39^K would be similar and so scalar coupling between ^31^P and ^39^K might be resolved in 1K·Tol, which has a similar environment about the P and K centres in the solid state to the P and Li centres in 1Li. However, ^39^K has a rather low magnetogyric ratio (*γ*(^39^K) = 1.2498 × 10^7^ rad T^−1^ s^−1^; *cf*. *γ*(^7^Li) = 10.3975 × 10^7^ rad T^−1^ s^−1^) and, since scalar coupling is directly proportional to this, the ^31^P–^39^K coupling constant will be commensurately small. Consistent with this, we find that the ^31^P{^1^H} CP-MAS NMR spectrum of 1K·Tol consists of a broad singlet at −87.2 ppm with no evidence for ^31^P–^39^K coupling.

In order to better understand why ^31^P–^39^K coupling is not observed in 1K·Tol we have calculated the reduced coupling constant *K* [*K* = (4π^2^/*h*)(*J*/*γ*_A_*γ*_B_)], which eliminates the dependency of the magnitude of the coupling constant on the magnetogyric ratios of the coupled nuclei, for the compounds 1Li, 1Na, 1Rb and 1Cs·Et_2_O, using the values of ^1^*J*(^31^P–^*n*^M) obtained from the CP-MAS solid state ^31^P{^1^H} NMR spectra (see Table 1). Various correlations have been proposed between the value of *K* and the atomic numbers *Z* of the coupled nuclei. A simple linear relationship has been proposed separately by McFarlane, by Dalling and Gutowsky, and by Wasylishen and co-workers,^27^ while a linear relationship between *Z* and the square root of *K* has been proposed by Reeves and co-workers.^28^ Since in the present series only the alkali metal nucleus is varied it is necessary only to consider the variation of *K* with the atomic number of this atom. A plot of *K versus* atomic number of the alkali metal (Fig. 6) gave a surprisingly good fit to a straight line, allowing a reasonable estimation of *K*(^31^P–^39^K) = 235 × 10^19^ T^2^ J^−1^ and therefore ^1^*J*(^31^P–^39^K) = 53 Hz. A very slightly better straight-line fit is obtained when √*K* is plotted against *Z*, leading to values of *K*(^31^P–^39^K) = 197 × 10^19^ T^2^ J^−1^ and ^1^*J*(^31^P–^39^K) = 45 Hz. Either of these values of *J* is sufficiently small that the coupling is subsumed into the natural linewidth of the quadrupolar broadened ^31^P{^1^H} NMR signal and so ^31^P–^39^K coupling is not resolved.

**Fig. 6:**
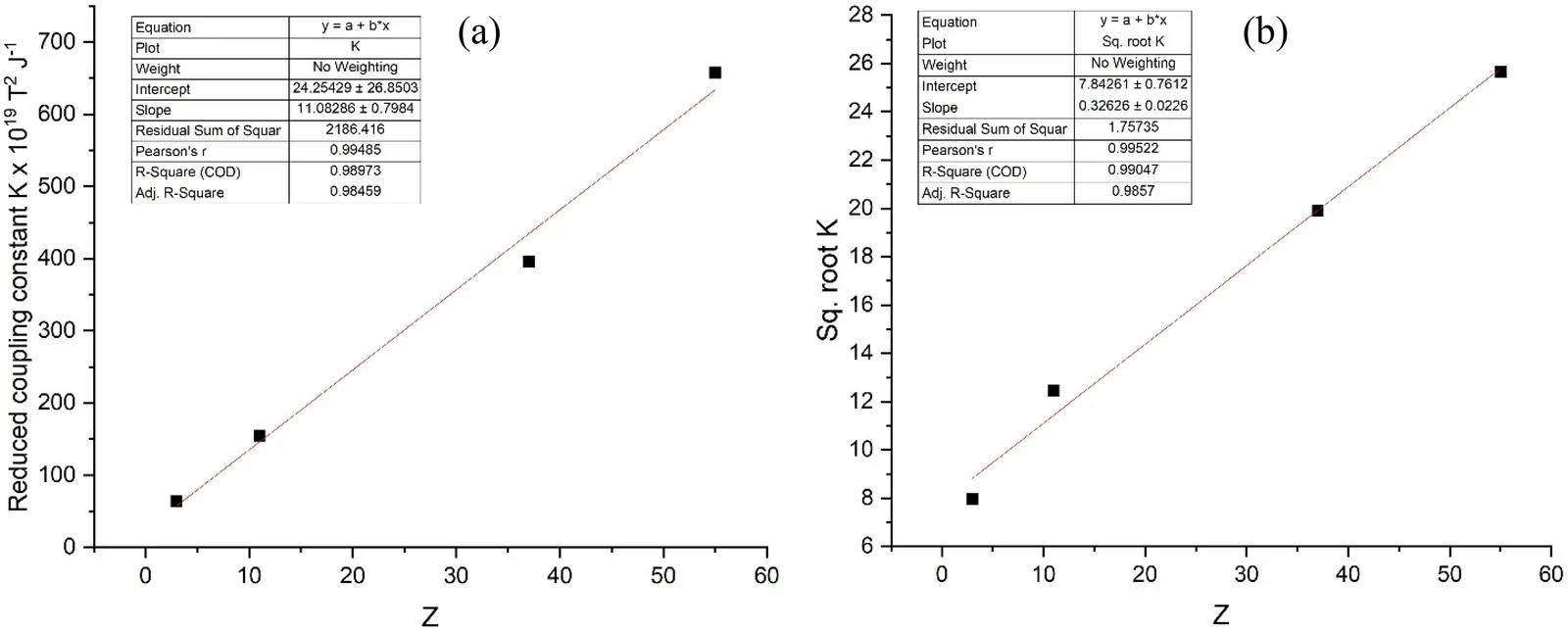
Plots of atomic number of the metal (*Z*) *vs.* (a) the reduced coupling constant *K* (10^19^ T^2^ J^−1^) and (b) √*K*.

## Conclusions

The super-bulky secondary chlorophosphane (^Me^Bhp)_2_PCl (1Cl) is readily accessible, in spite of the large steric bulk of the two aryl substituents. While 1Cl may be converted to the secondary phosphane (^Me^Bhp)_2_PH (1H), this compound does not undergo straightforward deprotonation at the phosphorus centre. However, the lithium phosphanide {(^Me^Bhp)_2_P}Li(THF)_2_·1/2PhMe (1Li) may be synthesised through the reaction of 1Cl with elemental lithium and this compound provides access to the heavier alkali metal complexes {(^Me^Bhp)_2_P}ML [ML = Na(OEt_2_) (1Na), K(PhMe)·2.5PhMe (1K·Tol), K(THF)_3_·2PhMe (1K·THF), Rb (1Rb) and Cs(OEt_2_)·Et_2_O (1Cs·Et_2_O)], through metathesis reactions between 1Li and the corresponding alkali metal alkoxides. The short M–P bonds, combined with the sp^2^-hybridised phosphorus centre in the majority of these complexes lead to a significant downfield shift of the ^31^P chemical shift of 39.0 ppm, suggestive of a significant relativistic spin–orbit heavy atom on light atom contribution to the chemical shift. These structural features also enable the observation of ^31^P–^*n*^M scalar coupling for the first time for ^23^Na, ^85/87^Rb and ^133^Cs. For 1Li and 1Cs·Et_2_O this coupling is observed both in the solid state and in solution, whereas for 1Na and 1Rb coupling is observed only in the solid state due to the poor solubility of these compounds in less polar solvents. For 1K·Tol scalar coupling is not resolved due to the small value of the ^31^P–^39^K coupling constant and broadening of the ^31^P NMR signal due to the quadrupolar nature of the ^39^K nucleus. Given the prevalence of M⋯arene interactions in the solid-state structures of 1M it is possible that these play at least some role in stabilising the M–P contacts in solution and thus in enabling the observation of ^133^Cs–^31^P scalar coupling, where dynamic exchange might be expected to be rather rapid.

The extreme steric bulk of the (^Me^Bhp)_2_P ligand suggests that it will form unique low-coordination number complexes with elements from other groups of the periodic table. The tendency of this ligand to adopt a planar configuration at phosphorus and the likely short M–P distances in these compounds may enable the observation of new ^31^P–^*n*^M scalar coupling, providing unparalleled insight into bonding and solution-state structure in the future.

## Author contributions

Ahmed Hadadi—methodology, formal analysis, investigation, data curation, writing—review & editing. Corinne Y. Wills—formal analysis, investigation, data curation, writing—review & editing. Casey M. Dixon—formal analysis, investigation, data curation, writing—review & editing. Paul G. Waddell—formal analysis, investigation, data curation, writing—review & editing. Keith Izod—conceptualization, methodology, validation, formal analysis, data curation, writing—original draft, supervision.

## Conflicts of interest

The authors declare no conflict of interest.

## Supplementary Material

DT-055-D6DT02307J-s001

## Data Availability

The data supporting this article have been included as part of the supplementary information (SI). Supplementary information: details of the synthesis and characterization of all compounds; details of structure determination, atomic coordinates, bond lengths and angles, and displacement parameters in CIF format; ^1^H, ^13^C{^1^H}, and ^31^P{^1^H} NMR spectra of all compounds and ^7^Li and ^133^Cs NMR spectra where applicable; details of DFT calculations, calculated atomic coordinates and final energies. See DOI: https://doi.org/10.1039/d6dt02307j. Ref. 30–38 are cited in the SI. CCDC 2540444 and 275916 (for two polymorphs of 1H), 2540445 (for 1H·Ben), 2540446 (for 1H·Tol), 2540447 (for 1H·THF), 2540448 (for 1Cl), 2540449 and 2575917 (for two polymorphs of 1Li), 2540450 (for 1Na), 2540451 (for 1K·THF), 2540452 (for 1K·Tol), 2540453 (for 1Rb) and 2540454 (for 1Cs·Et_2_O) contain the supplementary crystallographic data for this paper.^39*a*–*m*^
