# Supplementary material for: A super-bulky phosphanide ligand enables the observation of scalar coupling between 31P and 7Li, 23Na, 85Rb/87Rb, and 133Cs
Source: Dalton Trans. 2026 Oct 21. Online ahead of print. doi: 10.1039/d6dt02307j (PMC13711305; doi:10.1039/d6dt02307j)
Supplement: Supplementary file 1 [file DT-OLF-D6DT02307J-s001.pdf]

Supplementary Information (SI) for Dalton Transactions.
This journal is © The Royal Society of Chemistry

A Super-Bulky Phosphanide Ligand Enables the Observation of Scalar Coupling Between ^{31}P and ^{7}Li, ^{23}Na, ^{85}Rb/^{87}Rb, and ^{133}Cs

Ahmed Hadadi,[a,b] Corinne Y. Wills,[a] Casey M. Dixon,[a] Paul G. Waddell,[a] and Keith Izod*[a]

[a]*Main Group Chemistry Laboratories, School of Chemistry, Newcastle University, Newcastle upon Tyne, NE1 7RU, UK.* [b]*Department of Chemistry, Imam Mohammad Ibn Saud Islamic University (IMSIU), Riyadh 11623, Saudi Arabia*

SUPPORTING INFORMATION

Contents:

S3-S8 Details of synthetic procedures; syntheses of **1Cl**, **1H**, **1Li**, **1Na**, **1K**, **1Rb** and **1Cs**.

S9 Details of DFT calculations.

S9-S10 Additional references.

S11 Figure S1. Molecular structure of **1H**. Figure S2. Molecular structure of **1Cl**.

S12-S23 Figures S3-S25. Solution state ^{1}H, ^{13}C{^{1}H}, ^{31}P{^{1}H}, ^{7}Li, and ^{133}Cs NMR spectra of **1Cl**, **1H**, **1Li**, **1Na**, **1K**, **1Rb**, and **1Cs⋅Tol**.

S23 Figure S26. Variable-temperature ^{31}P{^{1}H} NMR spectra of **1Cs⋅Et$_2$O** in d_8-toluene.

S24-S27 Figures S27-S34. Solid state ^{31}P{^{1}H}, ^{7}Li, ^{23}Na, and ^{133}Cs NMR spectra of **1Li**, **1Na**, **1K**, **1Rb**, and **1Cs□Et$_2$O**.

S28-S30 Table S1. Crystallographic data for **1H**, **1H⋅THF**, **1H⋅Tol**, **1H□Ben**, **1Cl**, **1Li**, **1Na**, **1K□Tol**, **1K⋅THF**, **1Rb**, and **1Cs□Et$_2$O**.

S31-S41 Converged geometries, atomic coordinates, final electronic energies, and final Gibbs free energies for **1Na$_{pyr}$**, **1Na$_{plan}$**, and **1Na□THF$_{plan}$**.

General Experimental:

All manipulations were carried out using standard Schlenk and dry-box techniques under an atmosphere of dry nitrogen or argon. THF, diethyl ether, toluene, *n*-hexane, methylcyclohexane, benzene, and light petroleum (b.p. 40-60 °C) were dried prior to use by distillation under nitrogen from sodium, potassium, or sodium/potassium alloy, as appropriate. THF was stored over activated 4A molecular sieves; all other solvents were stored over a potassium film. Deuterated benzene, toluene and THF were distilled from potassium and $CDCl_3$ was distilled from CaH_2 under argon; all NMR solvents were deoxygenated by three freeze-pump-thaw cycles and were stored over activated 4Å molecular sieves. ^{Me}BhpI was synthesized by a previously published procedure.[19] Rubidium and Caesium 2-ethylhexoxide were prepared as standard solutions by the reaction of rubidium and caesium metals with 2-ethylhexanol in either toluene or *n*-hexane, respectively. *n*-Butyllithium was purchased from Aldrich as a 2.5 M solution in hexanes and its concentration accurately determined by titration before use. Sodium- and potassium *t*-butoxide were dried under vacuum (100 °C/0.01 Torr) before use. All other compounds were used as supplied by the manufacturer.

Solution state ^{1}H, $^{13}C\{^{1}H\}$, and $^{31}P\{^{1}H\}$ NMR spectra were recorded on a Bruker AvanceIII HD 500 spectrometer operating at 500.13, 125.77, and 202.46 MHz, respectively, or a Bruker AvanceIII 300 spectrometer operating at 300.13, 75.48, and 121.49 MHz, respectively; solution state ^{7}Li NMR spectra were recorded on a Bruker AvanceIII 300 spectrometer operating at 116.64 MHz; solution state ^{133}Cs NMR spectra were recorded on a Bruker AvanceIII HD 500 spectrometer operating at 65.59 MHz. Chemical shifts are quoted in ppm relative to

tetramethylsilane (^{1}H, ^{13}C), external 1.0 M LiCl in D_2O (^{7}Li), external 0.1 M CsCl in D_2O (^{133}Cs), or external 85% H_3PO_4 (^{31}P).

Solid state NMR measurements were made using a Bruker Avance III HD spectrometer equipped with a 11.7 Tesla magnet and fitted with a 4 mm Magic Angle Spinning (MAS) probe. Operating frequencies were 500.13 (^{1}H), 194.37 (^{7}Li), 132.29 (^{23}Na), 202.46 (^{31}P) and 65.60 (^{133}Cs). All measurements were made at 298 K with a spinning rate of 10 kHz. ^{31}P{^{1}H} Cross-Polarization-Magic-Angle-Spinning (CP-MAS) measurements were made with contact times of 1080 – 2822 ms, relaxation delays of 10-60 seconds and scans of 256 to 8192. Spectra were externally referenced to 85% H_3PO_4. The Bruker 'onepulse' pulse program was used to acquire spectra of the ^{7}Li, ^{23}Na and ^{133}Cs nuclei in the corresponding compounds. For the ^{7}Li SSNMR analysis of **1Li**, 256 scans were acquired and a relaxation delay of 5 s was used; the spectrum was referenced to external LiCl (0 ppm). The ^{23}Na SSNMR analysis of **1Na** was acquired with 8192 scans and a relaxation delay of 8 s; the spectrum was referenced to external NaCl (7.9 ppm). The ^{133}Cs SSNMR analysis of **1Cs⋅Et$_2$O** was acquired with 1024 scans and a relaxation delay of 5 s; the external reference was CsI (271.05 ppm). Samples were prepared in a dry-box under dry, oxygen-free nitrogen and were packed into a standard Bruker 4 mm zirconia rotor fitted with a Kel-F cap. Spectra were obtained within a few hours of the sample preparation, during which time no observable degradation was observed.

Due to the air sensitivity of the reported compounds **1M** consistent elemental analyses could not be not obtained; evidence of purity for these compounds is furnished by their comprehensive NMR spectroscopic characterisation.

^{Me}BhpLi: This compound was prepared by a modified, previously published procedure.[30] To a cold (–40 °C) solution of 2,6-bis(benzhydryl)-4-methylphenyl-1-iodobenzene (22.00 g, 39.96 mmol) in toluene (70 mL) was added, dropwise, 1.1 equivalents of *n*BuLi (18.71 mL, 2.35 M in hexane, 43.96 mmol). This mixture was stirred for 3 hours at –40 °C, to give a colourless solid. The solid was isolated by filtration and washed with cold (–40 °C) toluene (10 mL) followed by cold (–40 °C) light petroleum (2 x 10 mL). Residual solvent was removed *in vacuo* to give 2,6-bis(benzhydryl)-4-methylphenyllithium as a colourless powder. Yield 19.68 g, 94.3%.

(^{Me}Bhp)$_2$PCl (1Cl): To a cold (–40 °C) solution of ^{Me}BhpLi (19.68 g, 37.65 mmol) in toluene (70 mL) was added, dropwise, 0.5 equivalents of a solution of PCl_3 in toluene (18.75 mL, 1.0 M, 18.75 mmol). This mixture was stirred overnight at –40 °C, then allowed to warm to room temperature. The solution was filtered and solvent was removed *in vacuo* from the filtrate to give a colourless solid, which was washed with hot methylcyclohexane (20 mL). Residual solvent was removed *in vacuo* to give **1Cl** as a colourless powder. Yield 12.30 g, 72%. Single crystals of **1Cl** were obtained from cold (3 °C) methylcyclohexane. Anal. Calcd. for $C_{66}H_{54}ClP$: C 86.7, H 6.0%; found: C 86.2, H 6.2%. ^{1}H NMR ($CDCl_3$, 298 K): δ 2.05 (s, 6H, Ar*Me*), 6.56 (s, 4H, C*H*Ph$_2$), 6.68-7.12 (m, 44H, ArH). ^{13}C{^{1}H} NMR ($CDCl_3$, 298 K): δ 21.7 (Ar*Me*), 53.3 (d, J_{PC} = 17.1 Hz, *C*HPh$_2$), 126.1 (d, J_{PC} = 3.0 Hz, Ar), 127.9 (d, J_{PC} = 2.0 Hz, Ar), 130.1, 131.6, 139.2, 143.9, 144.6 (Ar), 147.1 (d, J_{PC} = 17.1 Hz, Ar). ^{31}P{^{1}H} NMR ($CDCl_3$, 298 K): δ 87.1.

(^{Me}Bhp)$_2$PH (1H): To a cold (0 °C) solution of **1Cl** (6.83 g, 7.48 mmol) in THF (30 ml) was added solid $LiAlH_4$ (0.28 g, 7.48 mmol) in portions and this mixture was allowed to gradually warm to room temperature, with stirring, over 12 hours. The mixture was re-cooled to 0 °C and

degassed water (50 mL) was carefully added. The organic phase was decanted, the aqueous phase was extracted into a 1:1 mixture of THF and Et_2O (3 x 25 mL), and the combined organic extracts were dried over activated 4 Å molecular sieves overnight. The solution was filtered, and the solvent was removed *in vacuo* from the filtrate to give **1H** as an off-white powder. Yield 4.30 g, 66%. Single crystals of the solvates $(^{Me}Bhp)_2PH$⋅4PhMe, $(^{Me}Bhp)_2PH$⋅4PhH, and $(^{Me}Bhp)_2PH$⋅½PhMe.THF were obtained from solutions of **1H** in toluene at -30 °C, benzene at 5 °C, and a 1:1 mixture of toluene and THF at -30 °C, respectively. ^1H NMR ($CDCl_3$, 298 K): δ 2.09 (s, 6H, Ar*Me*), 4.67 (d, J_{PH} = 239.9 Hz, PH), 5.95 (s, 4H, C*H*Ph_2), 6.64-7.14, (m, 44H, ArH). $^{13}C\{^1H\}$ NMR ($CDCl_3$, 298 K): δ 21.8 (Ar*Me*), 55.3 (d, J_{PC} = 10.6 Hz, *C*HPh_2), 126.0 (d, J_{PC} = 3.0 Hz, Ar), 128.0, (Ar), 130.0 (d, J_{PC} = 15.1 Hz, Ar), 133.8 (d, J_{PC} = 25.1 Hz, Ar), 137.9, 143.7, 144.5 (Ar), 148.4 (d, J_{PC} = 10.6 Hz, Ar). ^{31}P NMR ($CDCl_3$, 298 K): δ –94.4 (d, J_{PH} = 239.9 Hz).

$\{(^{Me}Bhp)_2P\}Li(THF)_2(PhMe)_{0.5}$ (1Li): To a solution of **1Cl** (12.30 g, 13.46 mmol) in THF (40 mL) was added 2.1 equivalents of Li shot (0.196 g, 28.27 mmol). The mixture was sonicated for 1 h and then stirred at room temperature for 3 days. The solvent was removed *in vacuo* from the red solution and the resulting orange solid was extracted into hot toluene (2 x 20 mL) and filtered. The solvent was removed *in vacuo* from the filtrate and the solid was washed with light petroleum (30 mL). Residual solvent was removed *in vacuo* to give **1Li** as an orange powder. Yield 12.56 g, 86%. Single crystals of **1Li** were obtained from cold (–25 °C) toluene. ^1H NMR (C_6D_6, 298 K): δ 1.14 (m, 8H, THF), 2.03 (s, 6H, Ar*Me*), 2.10 (s, 1.5H, Ph*Me*), 2.91 (m, 8H, THF), 6.89-7.16 (m, 47H, ArH+*Ph*Me), 7.34 (d, J_{PH} = 4.5 Hz, 4H, C*H*Ph_2). $^{13}C\{^1H\}$ NMR (C_6D_6, 343 K): δ 21.4 (Ph*Me*), 21.6 (Ar*Me*), 25.4 (THF), 56.7 (d, J_{PC} = 12.9 Hz, *C*HPh_2), 68.4 (THF), 125.5 (Ar),

125.7 (*Ph*Me), 128.3 (Ar), 128.6 (*Ph*Me), 129.3 (*Ph*Me), 130.1 (Ar), 130.7 (Ar), 132.4 (*Ph*Me), 137.9, 147.5, 148.3 (Ar), 150.1 (d, J_{PC} = 44.1 Hz, Ar). ^{7}Li NMR (C_6D_6, 298 K): δ −0.9 (d, J_{PLi} = 115.7 Hz). ^{31}P{^{1}H} NMR (C_6D_6, 298 K): δ −101.6 (q, J_{PLi} = 115.7 Hz).

{(^{Me}Bhp)$_2$P}Na(OEt$_2$) (1Na): To a solution of **1Li** (0.50 g, 0.458 mmol) in toluene (15 mL) was added sodium *tert*-butoxide (0.044 g, 0.458 mmol) and this mixture was stirred for 30 min. The solvent was removed *in vacuo* and the crude red solid was washed thoroughly with light petroleum (25 mL). Residual solvent was removed *in vacuo* and the resulting red solid was crystallized by slow evaporation of a diethyl ether solution at room temperature to give the solvate **1Na**. Yield 0.32 g, 72%. ^{1}H NMR (d_8-THF, 298 K): δ 1.12 (t, 6H, Et_2O), 1.97 (s, 6H, Ar*Me*), 3.39 (q, 4H, Et_2O), 6.51-6.96 (m, 44H, ArH), 7.05 (d, J_{PH} = 4.8 Hz, 4H, C*H*Ph_2). ^{13}C{^{1}H} NMR (d_8-THF, 298 K): δ 15.7 (Et_2O), 21.7 (Ar*Me*), 56.9 (d, J_{PC} = 13.1 Hz, *C*HPh_2), 66.4 (Et_2O), 125.5, 128.0, 129.8, 131.0, 131.2, 147.9, 148.2 (Ar), 153.1 (d, J_{PC} = 47.3 Hz). ^{31}P{^{1}H} NMR (d_8-THF, 298 K): δ −100.3

{(^{Me}Bhp)$_2$P}K: To a solution of **1Li** (0.50 g, 0.458 mmol) in toluene (15 mL) was added potassium *tert*-butoxide (0.051 g, 0.458 mmol) and this mixture was stirred for 30 mins. The solvent was removed *in vacuo* and the crude red solid was washed thoroughly with light petroleum (25 mL). Residual solvent was removed *in vacuo* to give {(^{Me}Bhp)$_2$P}K (**1K**) as a red powder. Yield 0.36 g, 86%. Single crystals of [{(^{Me}Bhp)$_2$P}K(PhMe)⋅2.5PhMe]$_□$ (**1K⋅Tol**) were obtained from cold (−30 °C) toluene; single crystals of the alternative solvate {(^{Me}Bhp)$_2$P}K(THF)$_3$⋅(PhMe)$_2$ (**1K⋅THF**) were obtained from a cold (−30 °C) 1:1 toluene/THF solution. Data for **1K** - ^{1}H NMR (d_8-THF, 298 K): δ 1.97 (s, 6H, Ar*Me*), 6.50-6.96 (m, 44H, ArH), 7.13 (s, 4H, C*H*Ph_2).

$^{13}C\{^1H\}$ NMR (d_8-THF, 298 K): δ 21.9 (Ar*Me*), 57.1 (d, J_{PC} = 13.8 Hz, *C*HPh$_2$), 125.5, 128.1, 129.8, 130.7, 131.3, 147.3, 148.5 (Ar), 155.5 (d, J_{PC} = 56.7 Hz, Ar). $^{31}P\{^1H\}$ NMR (d_8-THF, 298 K): δ –88.4.

[{(^{Me}Bhp)$_2$P}Rb]$_□$ (1Rb): To a solution of **1Li** (0.50 g, 0.458 mmol) in toluene (15 mL) was added a solution of rubidium 2-ethylhexoxide in toluene (1.39 mL, 0.33 M, 0.458 mmol) and this mixture was stirred for 30 minutes. The solvent was removed *in vacuo* and the crude solid was washed thoroughly with light petroleum (25 mL). Residual solvent was removed *in vacuo* to give **1Rb** as a red powder. Yield 0.43 g, 97%. Single crystals of **1Rb** were obtained by slow evaporation of a diethyl ether solution at room temperature. ^1H NMR (d_8-THF, 298 K): δ 1.96 (s, 6H, Ar*Me*), 6.50-7.00 (m, 44H, ArH), 7.17 (d, J_{PH} = 5.1 Hz, 4H, C*H*Ph$_2$). $^{13}C\{^1H\}$ NMR (d_8-THF, 298 K): δ 21.9 (Ar*Me*), 57.1 (d, J_{PC} = 14.0 Hz, *C*HPh$_2$), 125.5, 128.1, 129.7, 130.6, 131.5 (Ar), 147.2 (d, J_{PC} = 7.8 Hz, Ar), 148.6 (Ar), 155.9 (d, J_{PC} = 58.9 Hz, Ar). $^{31}P\{^1H\}$ NMR (d_8-THF, 298 K): δ –81.7.

{(^{Me}Bhp)$_2$P}Cs⋅PhMe (1Cs⋅Tol): To a solution of **1Li** (0.50 g, 0.458 mmol) in toluene (15 mL) was added a solution of caesium 2-ethylhexoxide in hexane (6.11 mL, 0.075 M, 0.458 mmol) and this mixture was stirred for 30 min. The solvent was removed *in vacuo* and the crude solid was washed thoroughly with light petroleum (25 mL). Residual solvent was removed *in vacuo* to give **1Cs□Tol** as a red powder. Yield 0.40 g, 79%. Single crystals of the alternative solvate [{(^{Me}Bhp)$_2$P}Cs(OEt$_2$).Et$_2$O]$_□$ (**1Cs⋅Et$_2$O**) were obtained by slow evaporation of a diethyl ether solution at room temperature. Data for **1Cs□Tol** - ^1H NMR (C_6D_6, 298 K): δ 2.02 (s, 6H, Ar*Me*), 2.10 (s, 3H, Ph*Me*), 6.86 (m, 8H, ArH), 6.96 (m, 20H, ArH), 7.00-7.15 (m, 5H, *Ph*Me), 7.21 (m, 16H, ArH), 7.40 (d, J_{PH} = 4.8 Hz, 4H, C*H*Ph$_2$). $^{13}C\{^1H\}$ NMR (C_6D_6, 298 K): δ 21.4 (Ph*Me*),

21.7 (Ar*Me*), 56.5 (d, J_{PC} = 12.6 Hz, *C*HPh$_2$) 125.4 (Ar), 125.7 (*Ph*Me), 128.3, 128.6 (Ar), 129.3 (*Ph*Me), 129.6 (*Ph*Me), 131.0, 131.3 (Ar), 137.9 (*Ph*Me), 147.8 (d, J_{PC} = 7.5 Hz, Ar) 148.0 (Ar), 153.5 (d, J_{PC} = 62.9 Hz, Ar). ^{31}P{^{1}H} NMR (C_6D_6, 298 K): δ –62.6. ^{133}Cs NMR (d_8-toluene, 298 K): δ –50.1.

X-ray crystallography: Crystal structure datasets for all compounds were collected on a Rigaku Oxford Diffraction XtaLAB Synergy-S HyPix-Arc 100 diffractometer using copper radiation ($\lambda_{CuK\alpha}$ = 1.54184 Å). Using an Oxford Cryosystems CryostreamPlus open-flow N_2 cooling device, data for all structures were collected at 150 K, with the exception of **1Na** for which data were collected at 200 K as the crystals underwent a destructive phase transition at lower temperatures. Cell refinement, data collection, and data reduction were undertaken using CrysAlisPro.[31] Analytical numeric absorption correction using a multifaceted crystal model based on expressions derived by R. C. Clark and J. S. Reid[32] was applied in each case. The structures were solved using SHELXT[33] and refined by SHELXL[34] through the Olex2 interface.[35] Hydrogen atoms bound to carbon were positioned with idealised geometry and those attached to phosphorus were located from peaks in the Fourier difference map. In all cases, the displacement parameters for hydrogen atoms were constrained using a riding model.

DFT calculations:

Geometry optimisations were performed with the Gaussian16 suite of programs (revision C.02).[36] The B97D functional,[37] which includes Grimme's D2 correction for dispersion effects, was employed throughout and the 6-311G(2d,p) all-electron basis set[38] was used on all atoms. The

identity of minima was confirmed by the absence of imaginary vibrational frequencies in each case. Automatic density fitting was employed for all geometry optimisations and frequency calculations. Final energies were obtained from single point calculations (B97D/6-311G(2d,p)) on the converged geometries with implicit solvation by THF using the integral equation formulation polarisable continuum model (IEFPCM).

References:

30. B. G. Steller, B. Doler, R. C. Fischer. Diaryltin dihydrides and aryltin trihydrides with intriguing stability. *Molecules* **2020**, *25*, 1076.
31. CrysAlisPro, Agilent Technologies, Version 1.171.36.
32. R. C. Clark, J. S. Reid. The analytical calculation of absorption in multifaceted crystals. *Acta Crystallogr.* **1995**, *A51*, 887-897.
33. G. M. Sheldrick. SHELXT – Integrated space-group and crystal-structure determination. *Acta Crystallogr., Sect. A* **2015**, *71*, 3-8.
34. (a) G. M. Sheldrick. A short history of SHELX. *Acta Crystallogr., Sect. A* **2008**, *64*, 112-122. (b) G. M. Sheldrick. Crystal structure refinement with SHELXL. *Acta Crystallogr. Sect. C* **2015**, *71*, 3-8.
35. O. V. Dolomanov, L. J. Bourhis, R. J. Gildea, J. A. K. Howard, H. Puschmann. OLEX2: a complete structure solution, refinement and analysis program. *J. Appl. Crystallogr.* **2009**, *42*, 339-341.

36. Gaussian 16, Revision C.02, M. J. Frisch, G. W. Trucks, H. B. Schlegel, G. E. Scuseria, M. A. Robb, J. R. Cheeseman, G. Scalmani, V. Barone, G. A. Petersson, H. Nakatsuji, X. Li, M. Caricato, A. V. Marenich, J. Bloino, B. G. Janesko, R. Gomperts, B. Mennucci, H. P. Hratchian, J. V. Ortiz, A. F. Izmaylov, J. L. Sonnenberg, D. Williams-Young, F. Ding, F. Lipparini, F. Egidi, J. Goings, B. Peng, A. Petrone, T. Henderson, D. Ranasinghe, V. G. Zakrzewski, J. Gao, N. Rega, G. Zheng, W. Liang, M. Hada, M. Ehara, K. Toyota, R. Fukuda, J. Hasegawa, M. Ishida, T. Nakajima, Y. Honda, O. Kitao, H. Nakai, T. Vreven, K. Throssell, J. A. Montgomery, Jr., J. E. Peralta, F. Ogliaro, M. J. Bearpark, J. J. Heyd, E. N. Brothers, K. N. Kudin, V. N. Staroverov, T. A. Keith, R. Kobayashi, J. Normand, K. Raghavachari, A. P. Rendell, J. C. Burant, S. S. Iyengar, J. Tomasi, M. Cossi, J. M. Millam, M. Klene, C. Adamo, R. Cammi, J. W. Ochterski, R. L. Martin, K. Morokuma, O. Farkas, J. B. Foresman, and D. J. Fox, Gaussian, Inc., Wallingford CT, 2016.

37. S. Grimme, Semiempirical GGA-type density functional constructed with a long-range dispersion correction. *J. Comput. Chem.* 2006, **27**, 1787-1799.

38. a) A. D. McLean, G. S. Chandler, Contracted Gaussian basis sets for molecular calculations. I. Second row atoms, *Z*=11–18. *J. Chem. Phys.* 1980, **72**, 5639-5648. b) K. Raghavachari, J. S. Binkley, R. Seeger, J. A. Pople, Self-consistent molecular orbital methods. XX. A basis set for correlated wave functions. *J. Chem. Phys.*, 1980, **72**, 650-654. c) R. C. Binning Jr., L. A. Curtiss, Compact contracted basis sets for third-row atoms: Ga–Kr. *J. Comput. Chem.* 1990, **11**, 1206-1216. d) L. A. Curtiss, M. P. McGrath, J.-P. Blaudeau, N. E.

Davis, R. C. Binning Jr., L. Radom, Extension of Gaussian-2 theory to molecules containing third-row atoms Ga–Kr. *J. Chem. Phys.* 1995, **103**, 6104-6113.

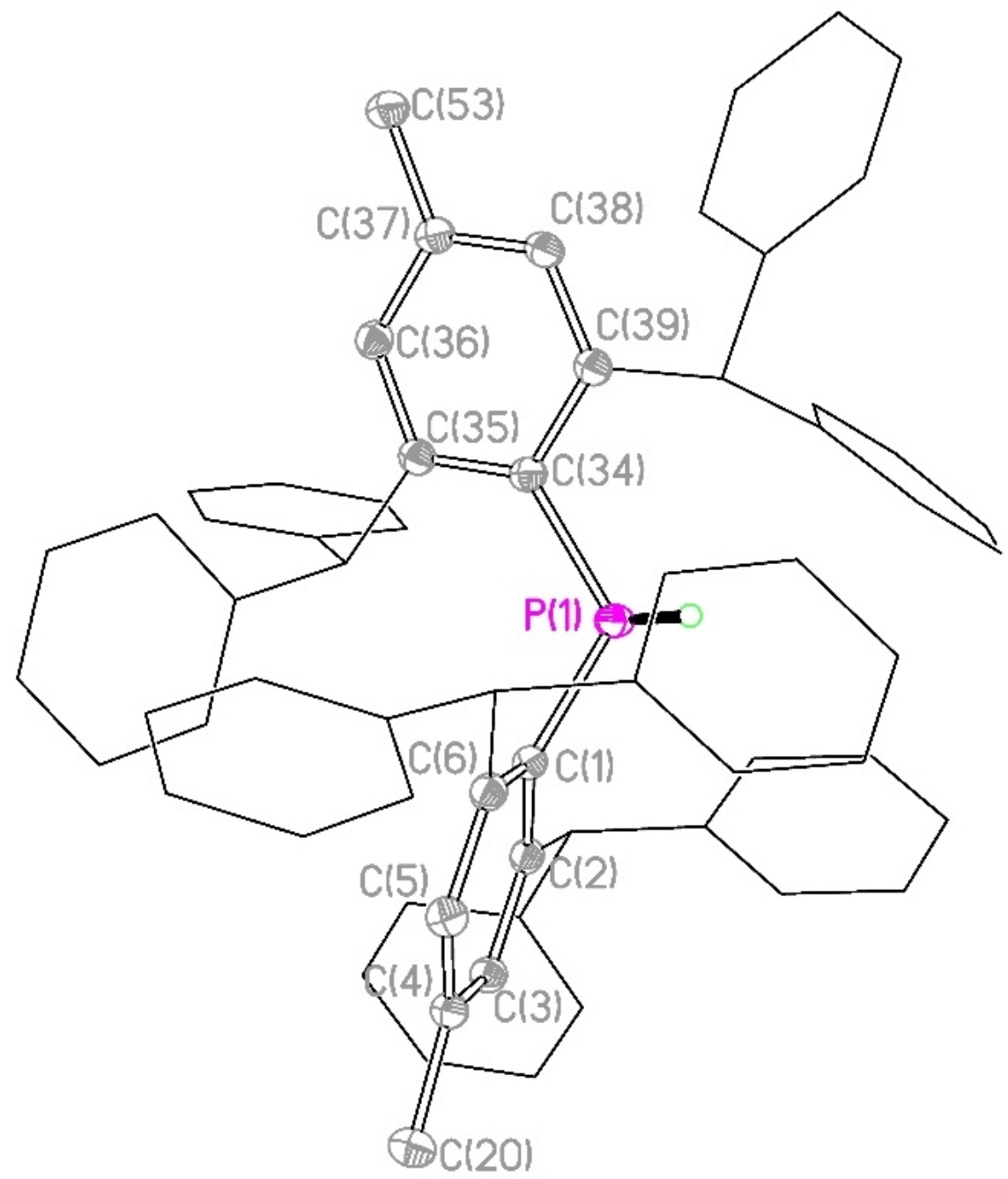

Figure S1. Molecular structure of **1H** with 40% probability ellipsoids and with C-bound H atoms omitted for clarity; benzhydryl groups shown in wireframe.

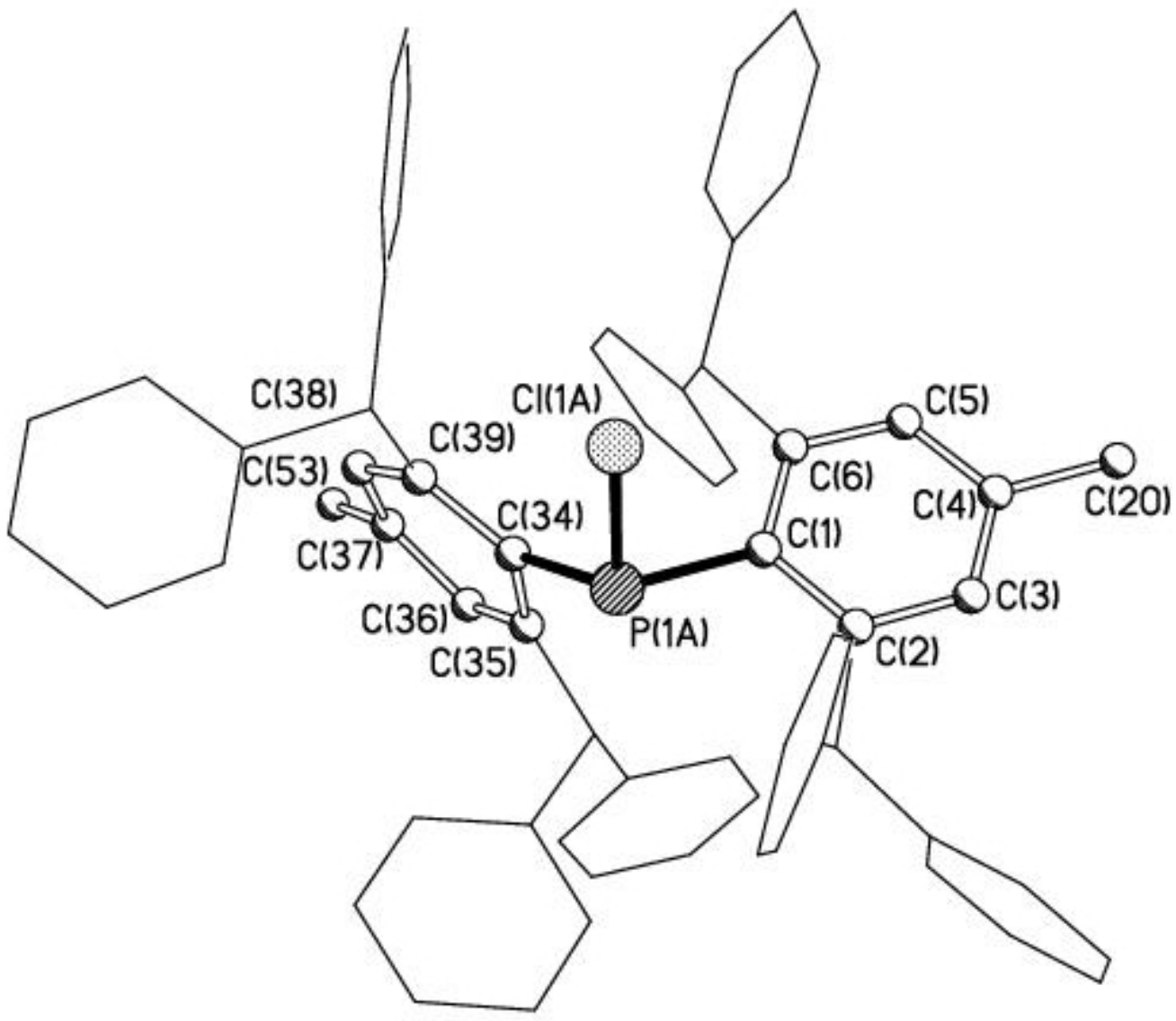

Figure S2. Molecular structure of **1Cl** with 40% probability ellipsoids and with H atoms omitted for clarity; benzhydryl groups shown in wireframe.

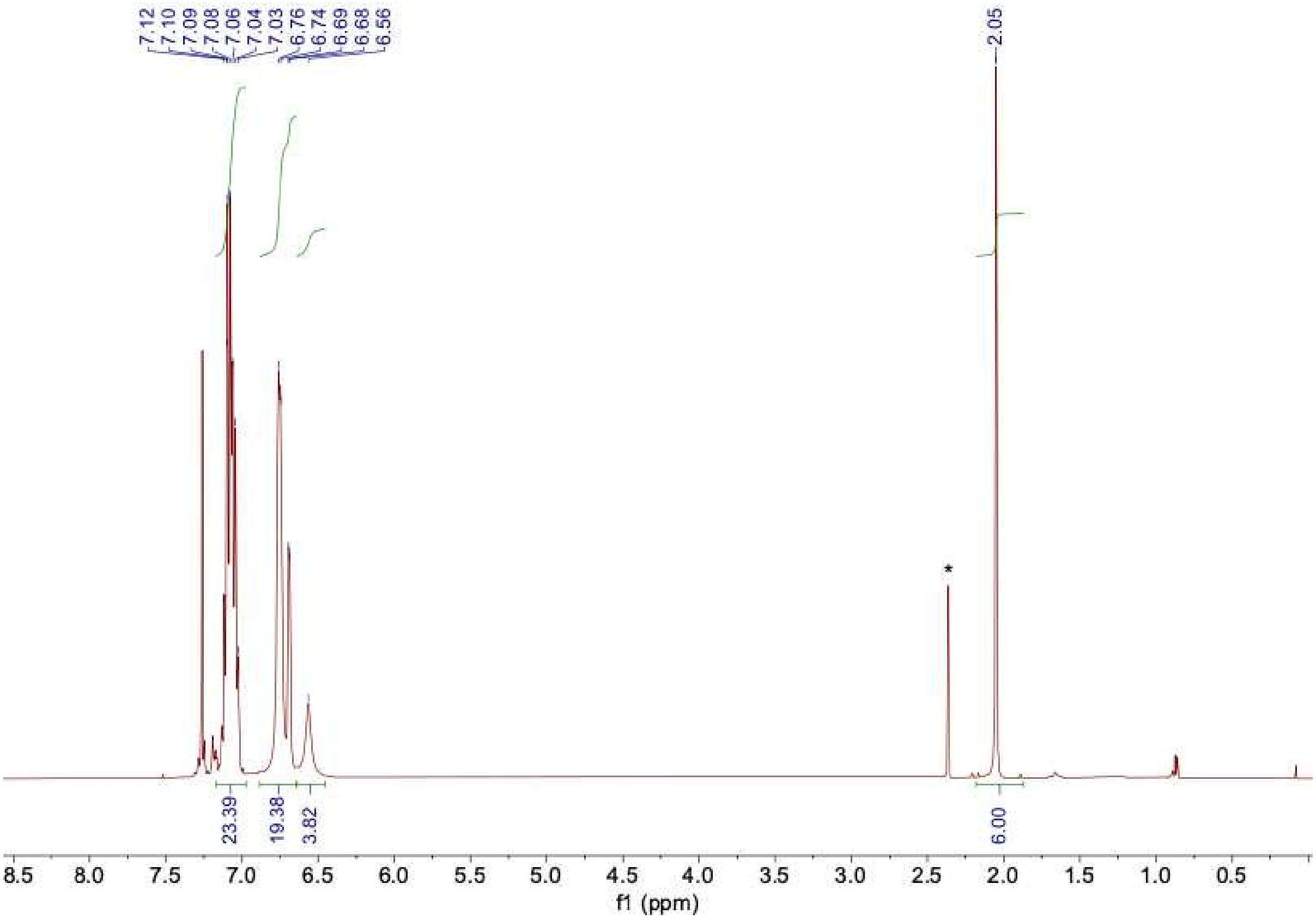

Figure S3. ^{1}H NMR spectrum of **1Cl** in $CDCl_3$ at 298 K. *Unknown impurity.

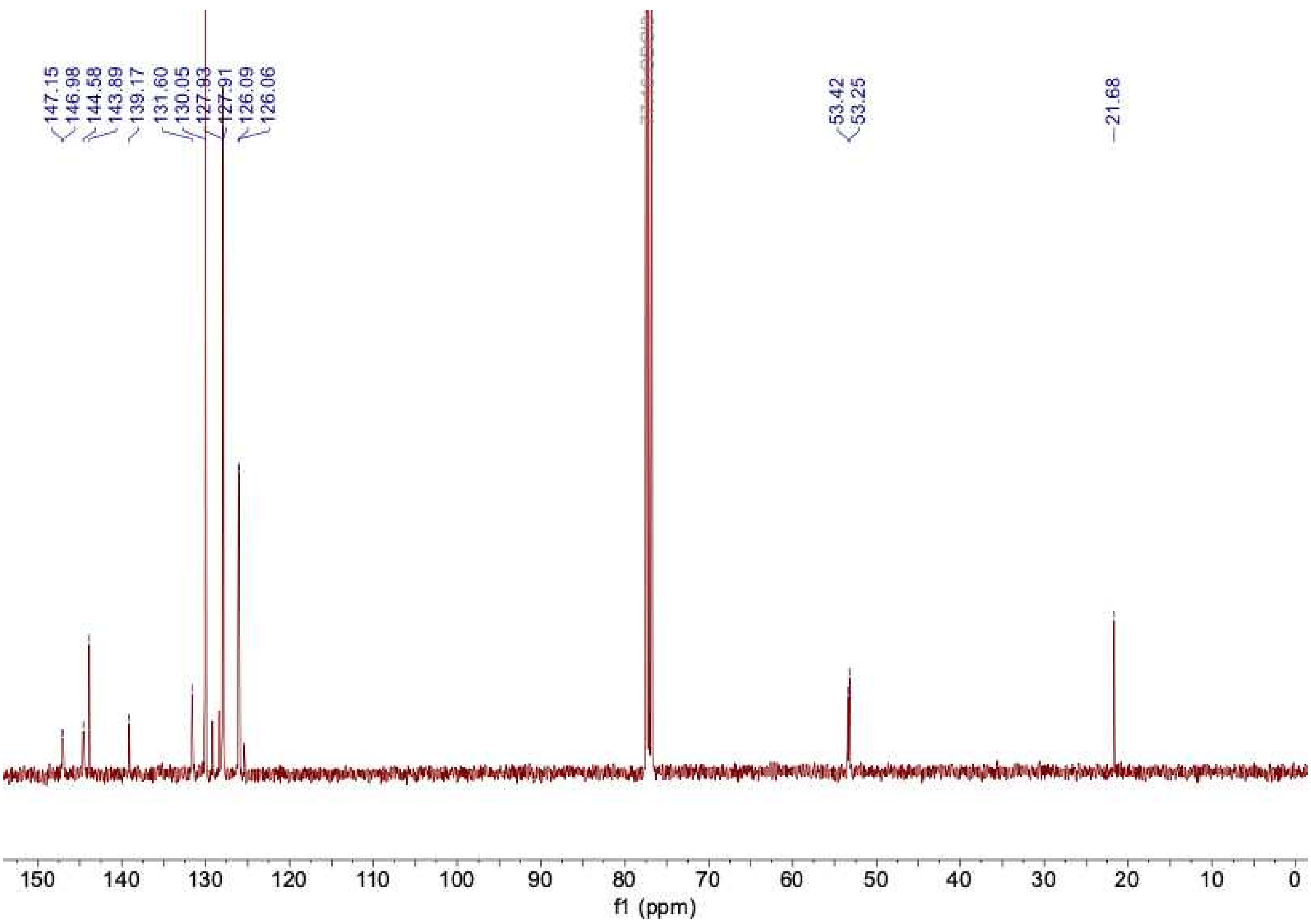

Figure S4. $^{13}C\{^1H\}$ NMR spectrum of **1Cl** in $CDCl_3$ at 298 K.

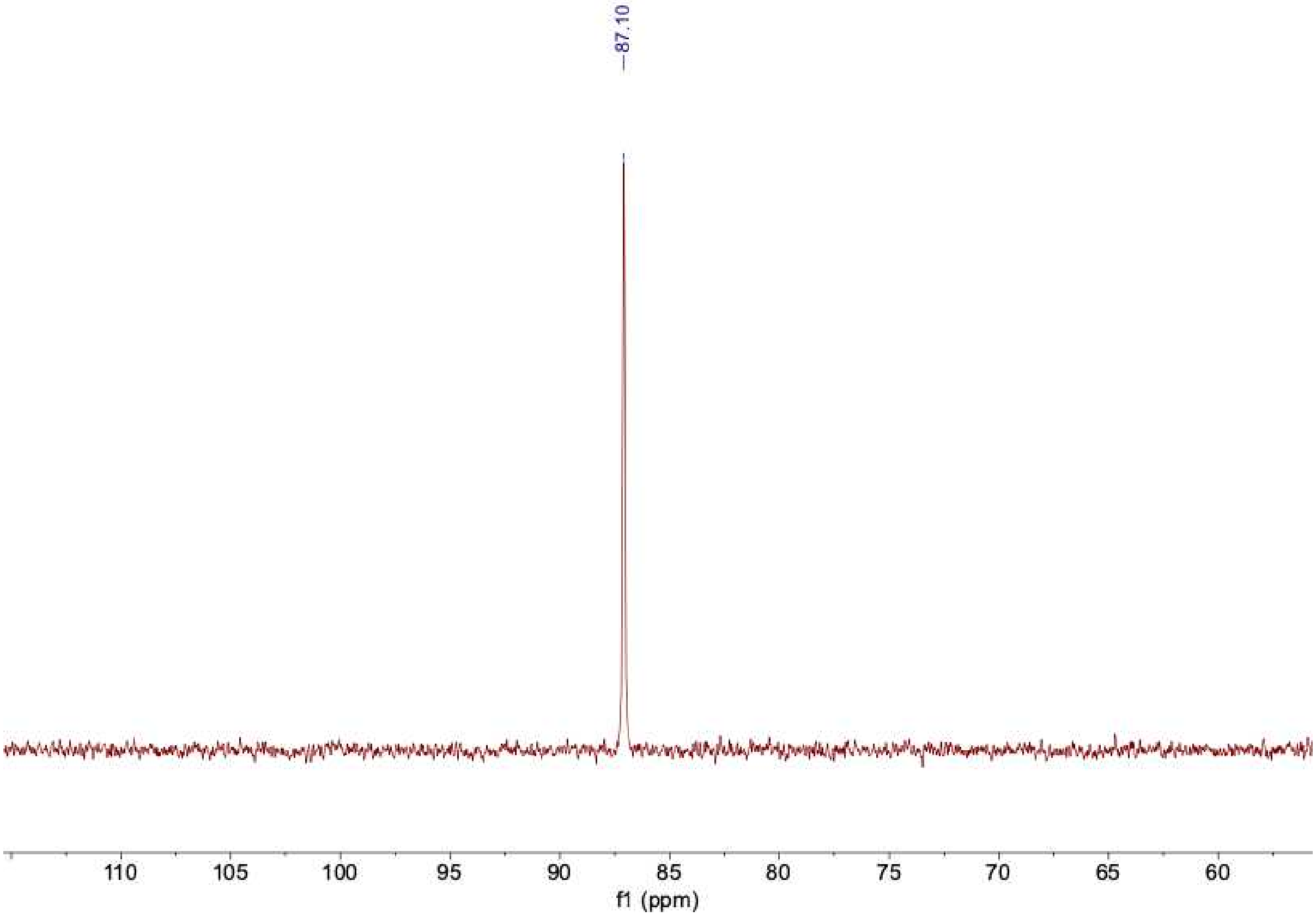

Figure S5. $^{31}P\{^1H\}$ NMR spectrum of **1Cl** in $CDCl_3$ at 298 K.

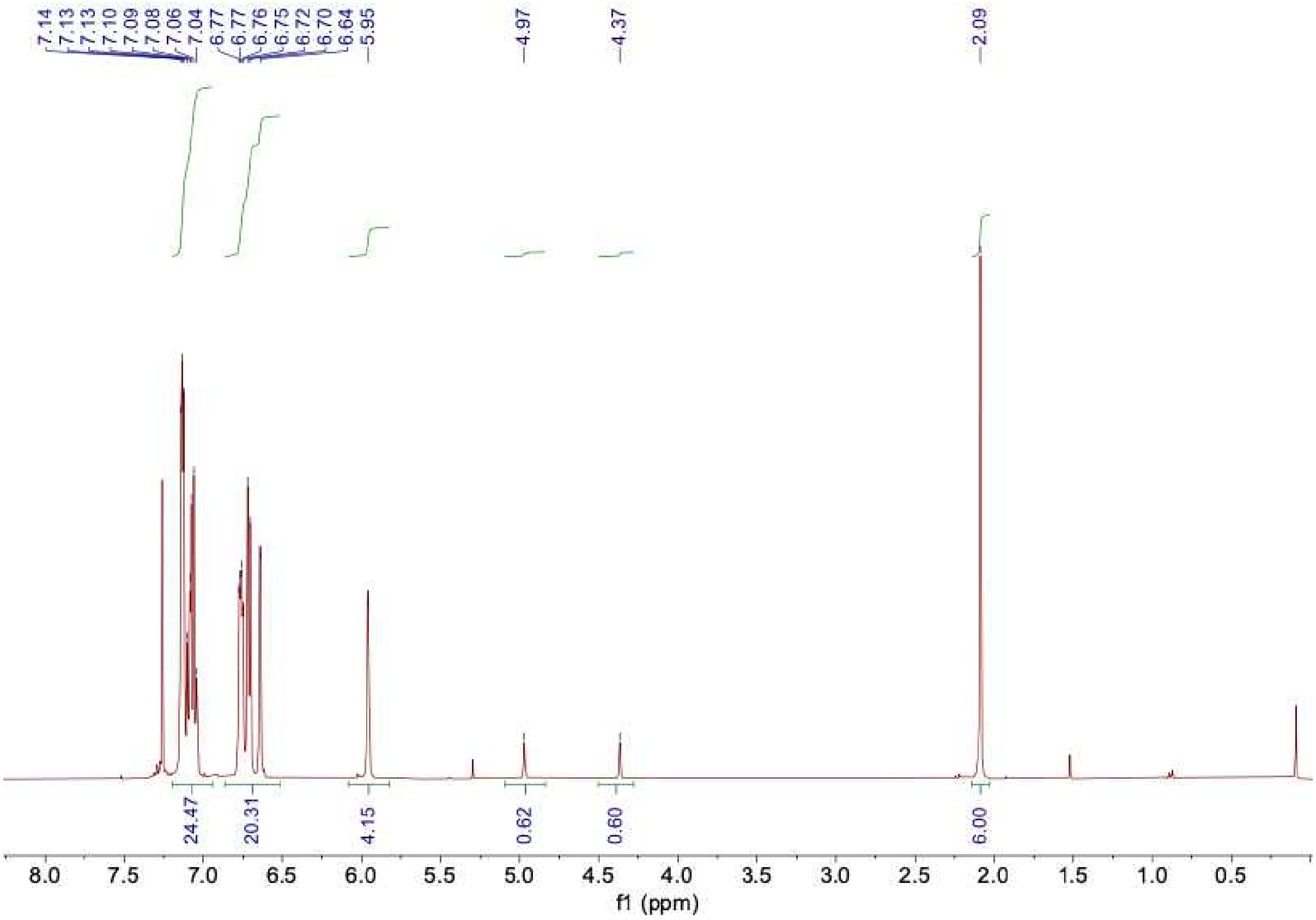

Figure S6. ^{1}H NMR spectrum of **1H** in $CDCl_3$ at 298 K.

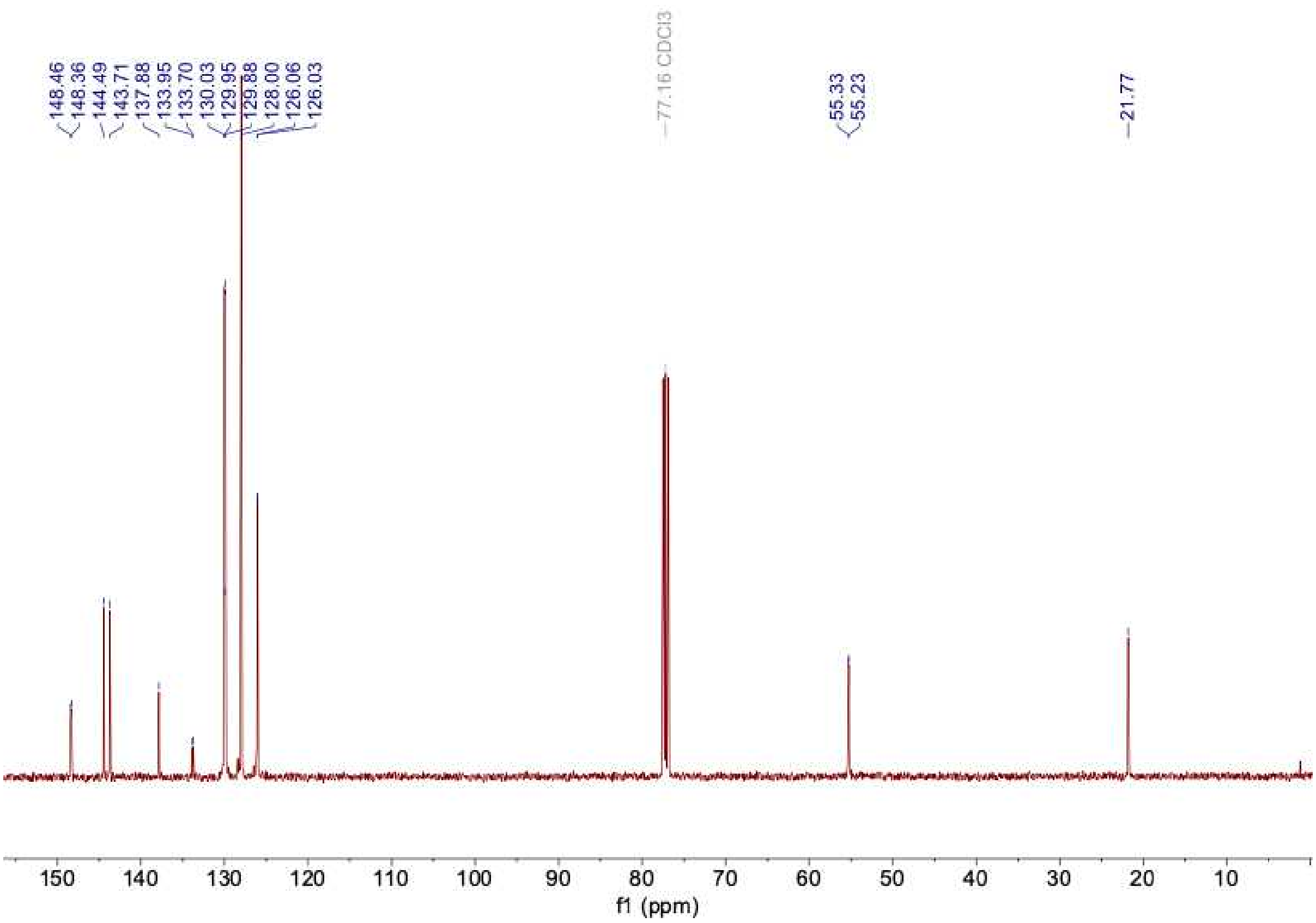

Figure S7. $^{13}C\{^{1}H\}$ NMR spectrum of **1H** in $CDCl_3$ at 298 K.

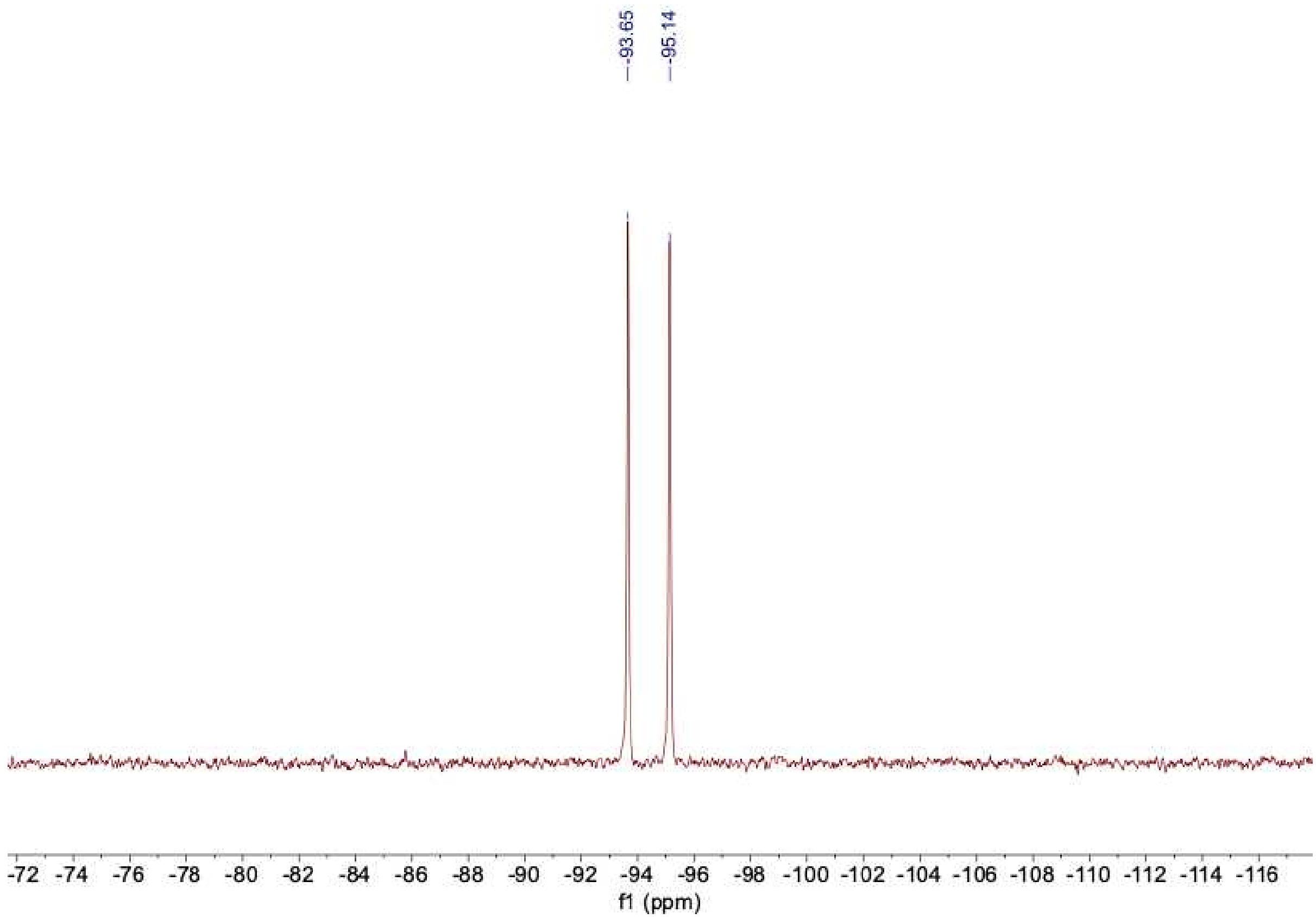

Figure S8. ^{31}P NMR spectrum of **1H** in $CDCl_3$ at 298 K.

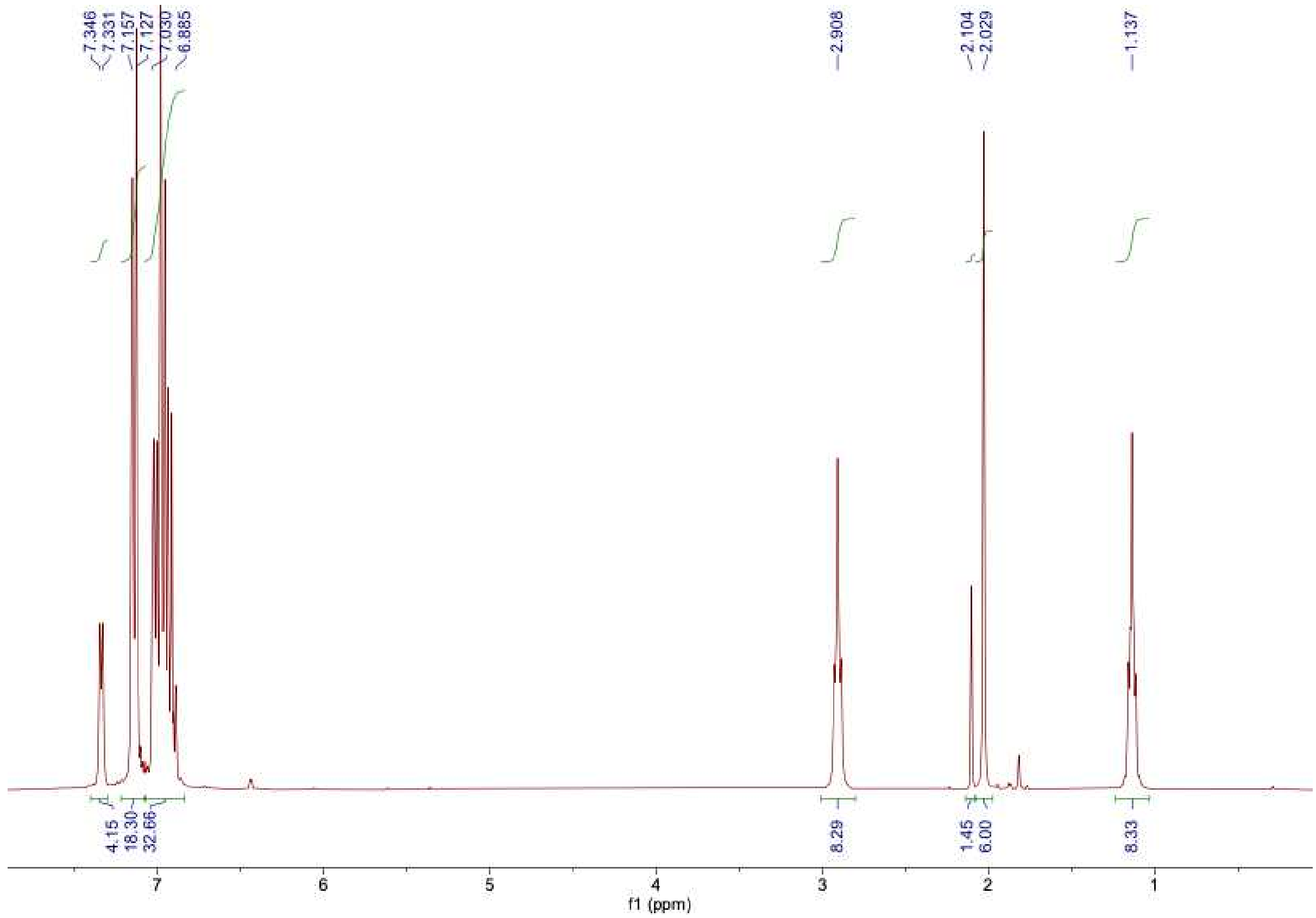

Figure S9. ^{1}H NMR spectrum of **1Li** in C_6D_6 at 298 K.

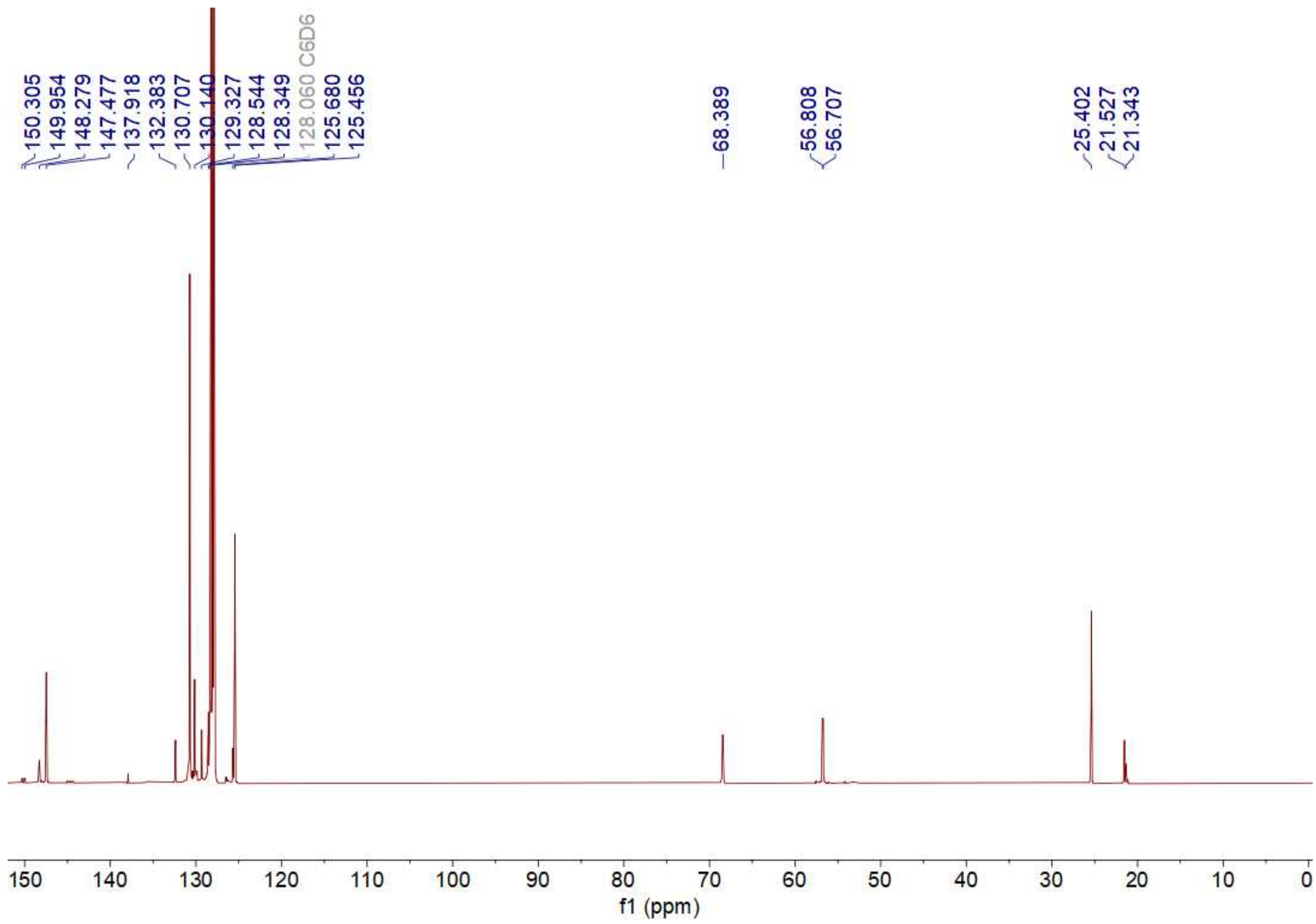

Figure S10. $^{13}C\{^{1}H\}$ NMR spectrum of **1Li** in C_6D_6 at 298 K.

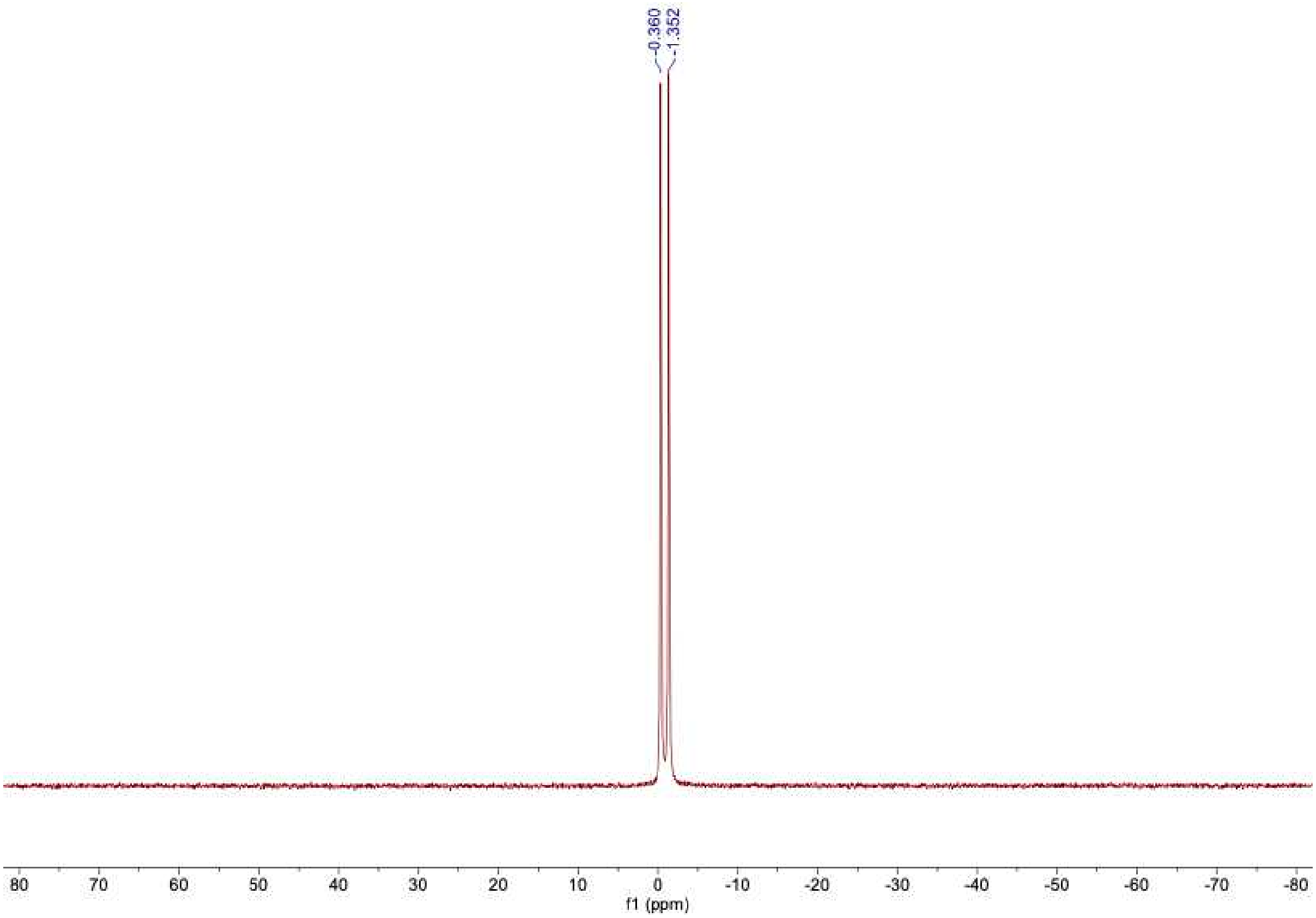

Figure S11. ^{7}Li NMR spectrum of **1Li** in C_6D_6 at 298 K.

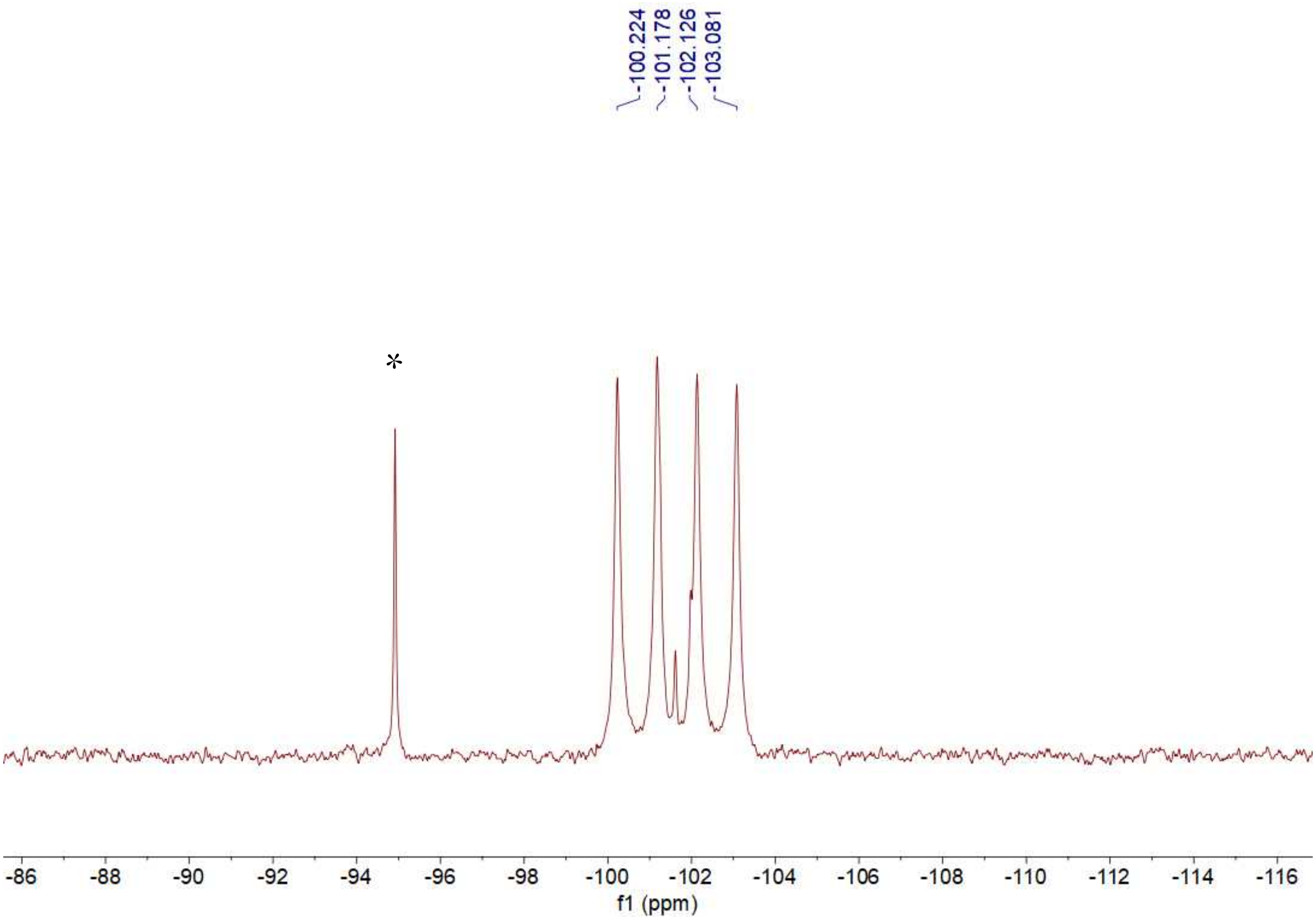

Figure S12. $^{31}P\{^1H\}$ NMR spectrum of **1Li** in C_6D_6 at 298 K. *Residual **1H** due to partial hydrolysis.

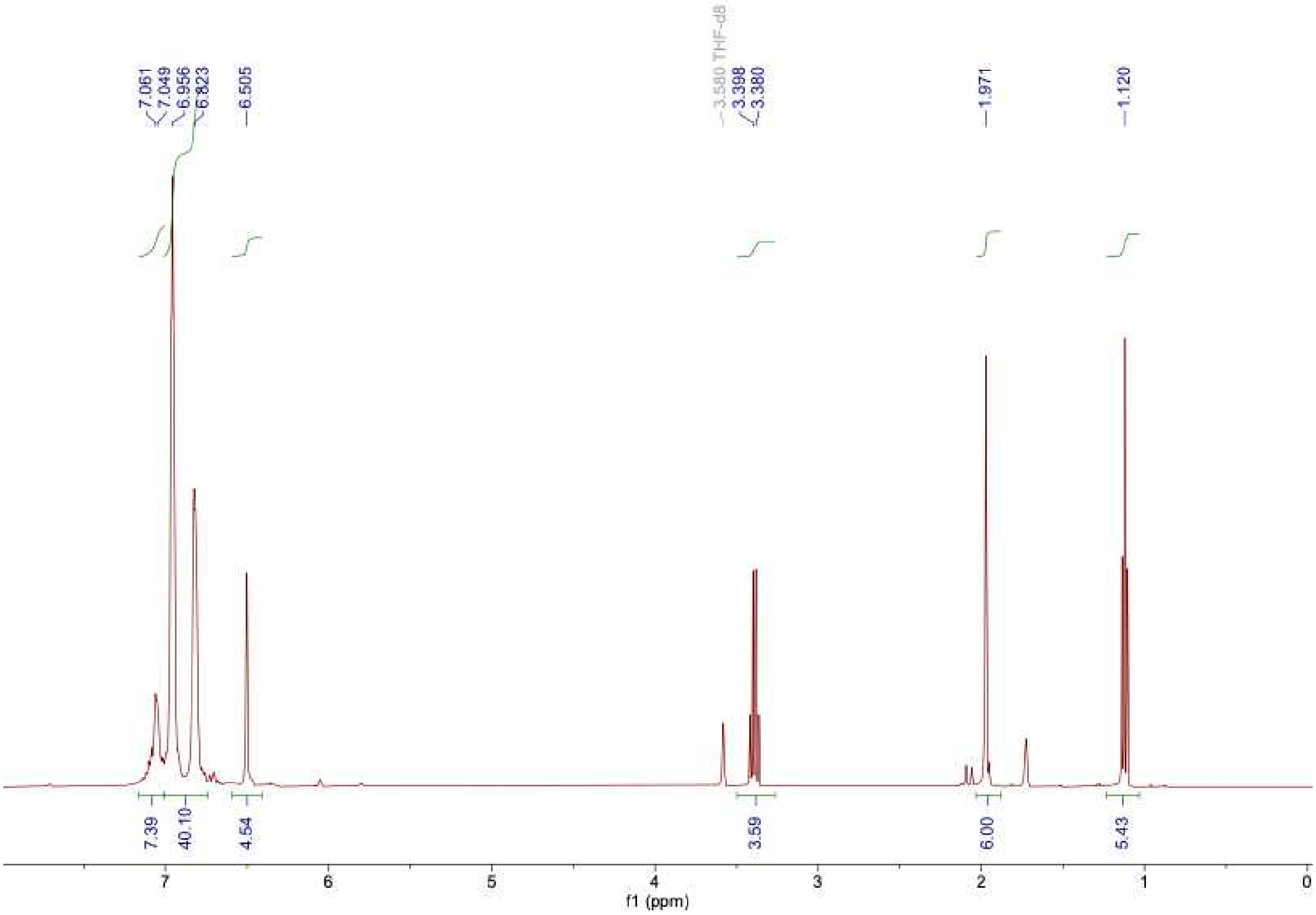

Figure S13. ^1H NMR spectrum of **1Na** in d_8-THF at 298 K.

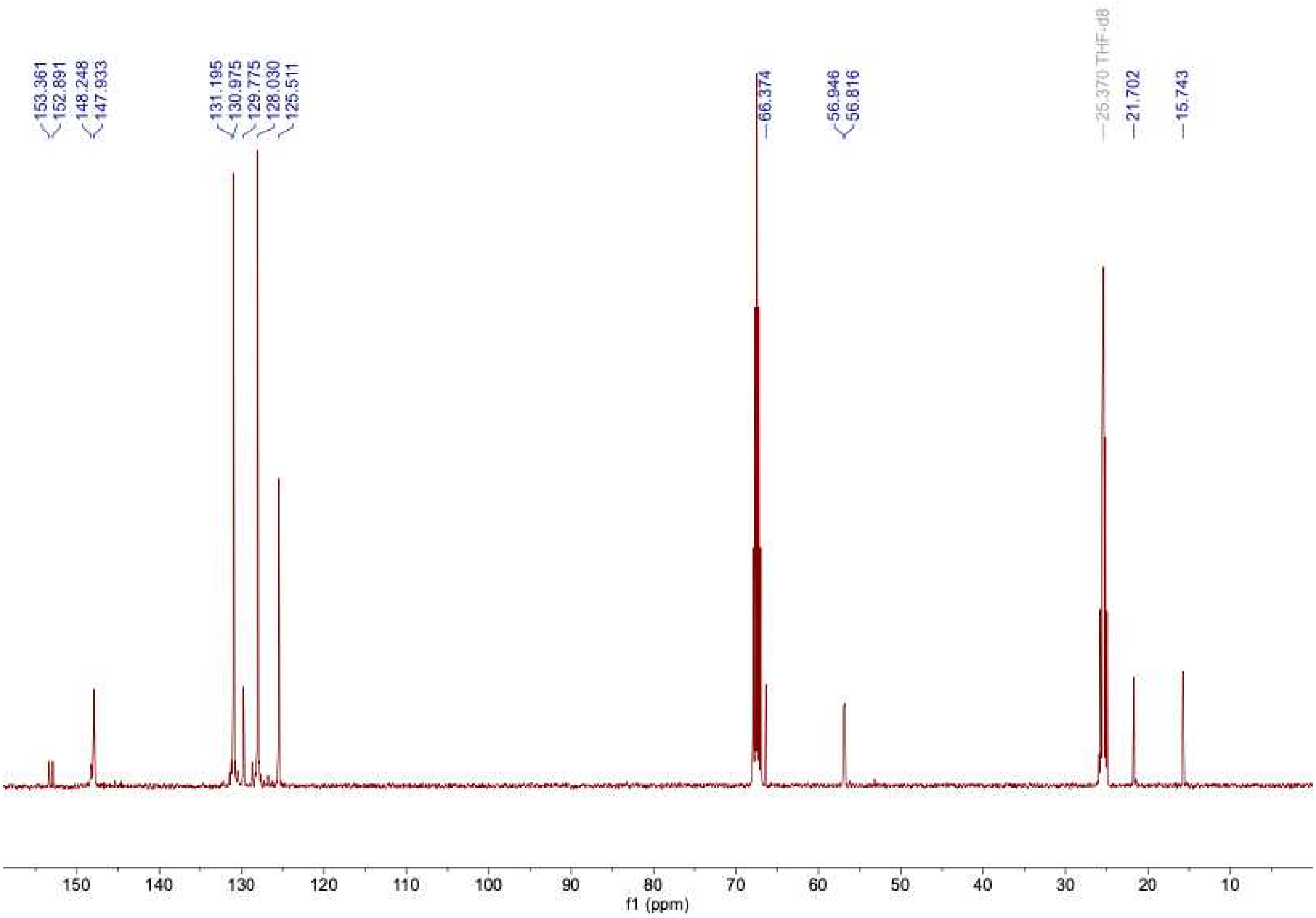

Figure S14. $^{13}C\{^{1}H\}$ NMR spectrum of **1Na** in d_8-THF at 298 K.

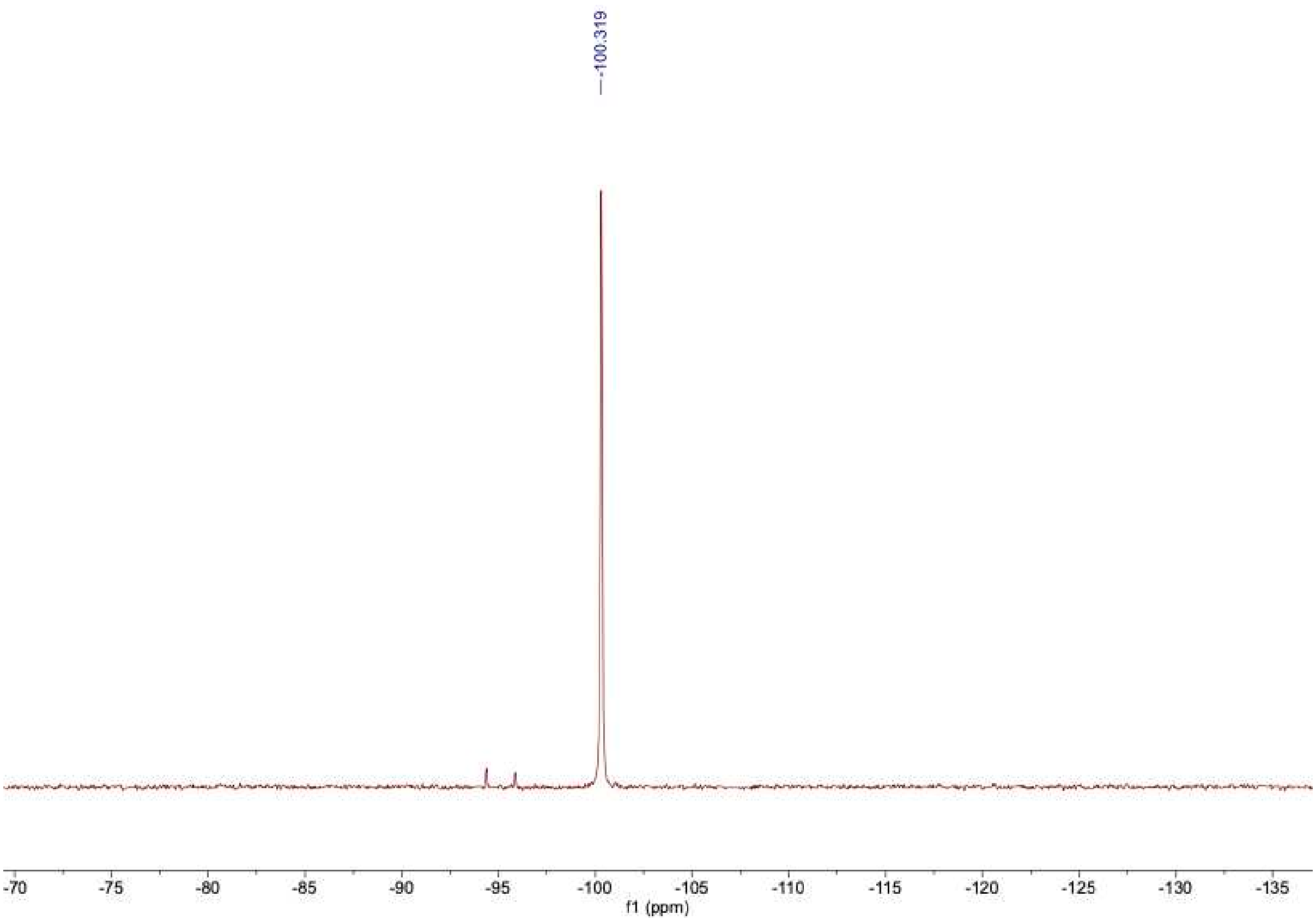

Figure S15. $^{31}P\{^{1}H\}$ NMR spectrum of **1Na** in d_8-THF at 298 K.

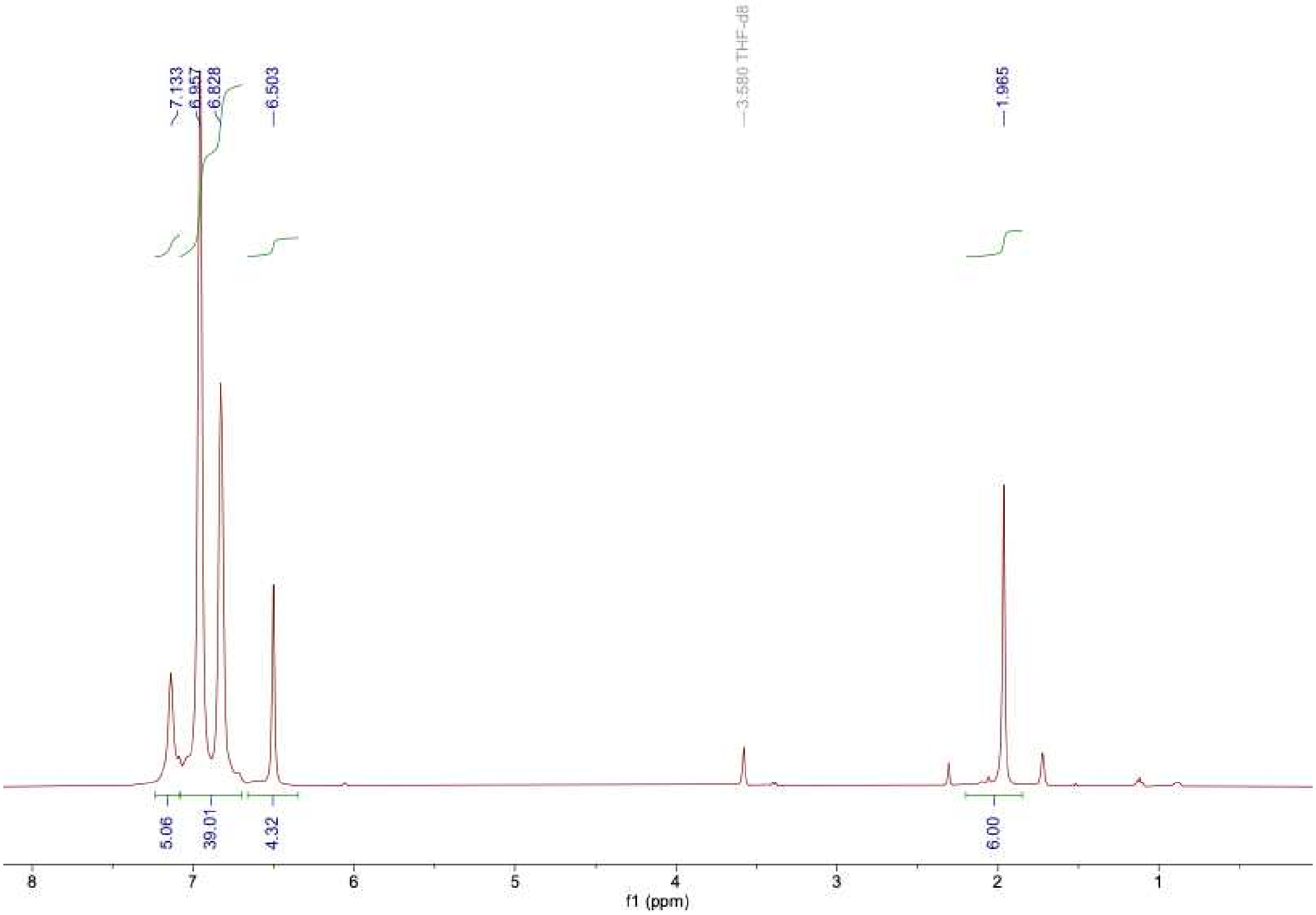

Figure S16. ^{1}H NMR spectrum of **1K** in d_8-THF at 298 K.

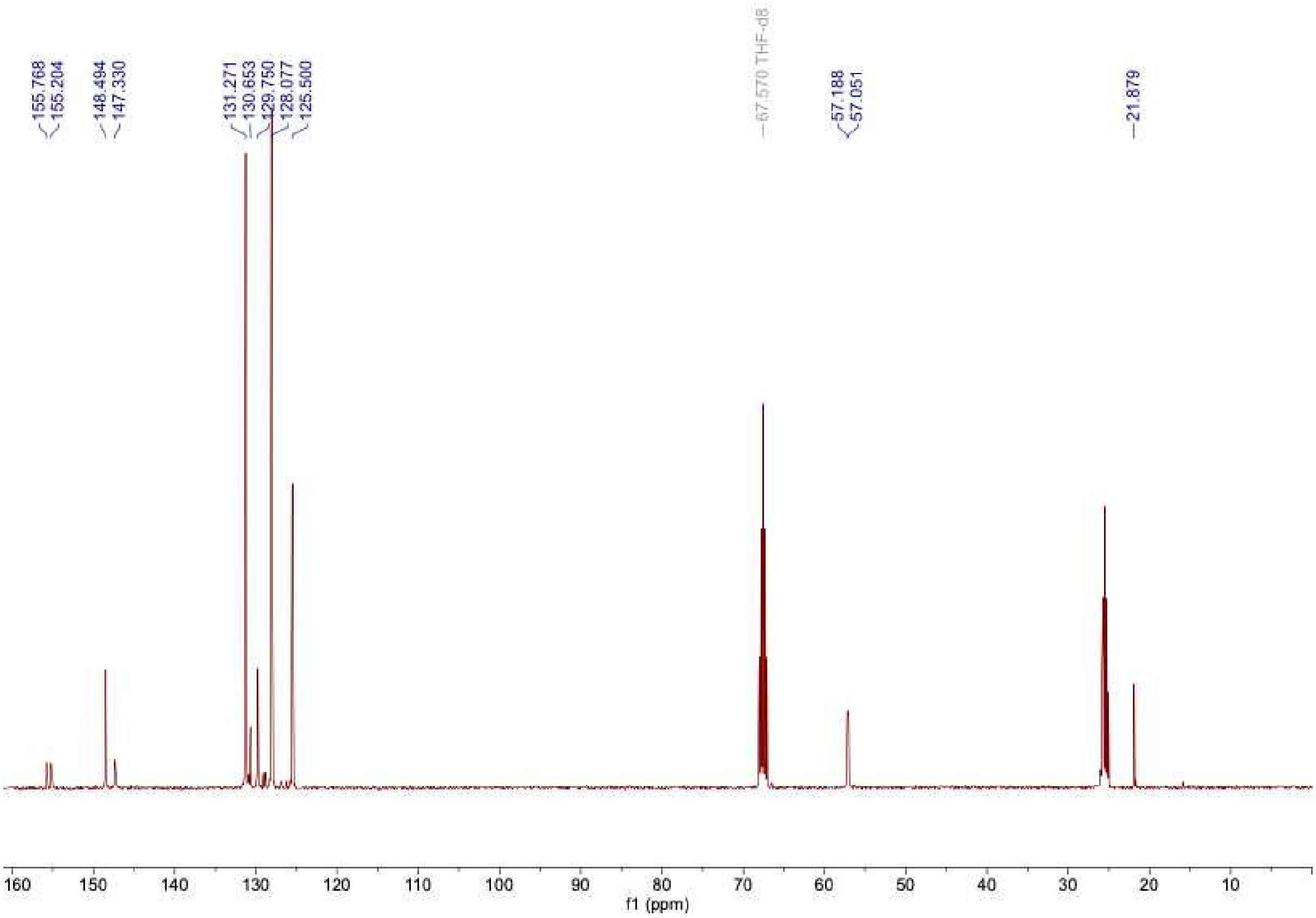

Figure S17. $^{13}C\{^{1}H\}$ NMR spectrum of **1K** in d_8-THF at 298 K.

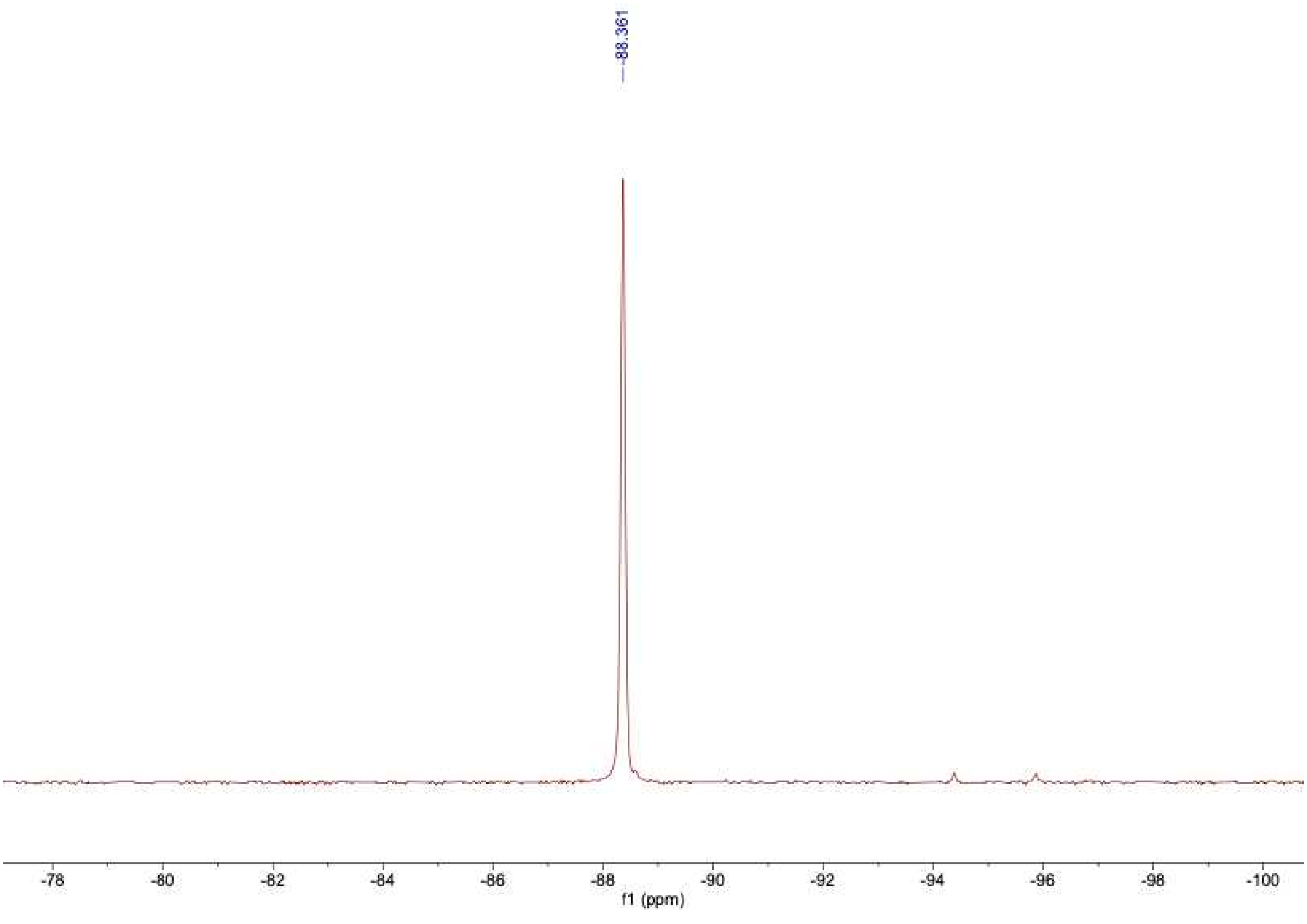

Figure S18. $^{31}P\{^{1}H\}$ NMR spectrum of **1K** in d_8-THF at 298 K.

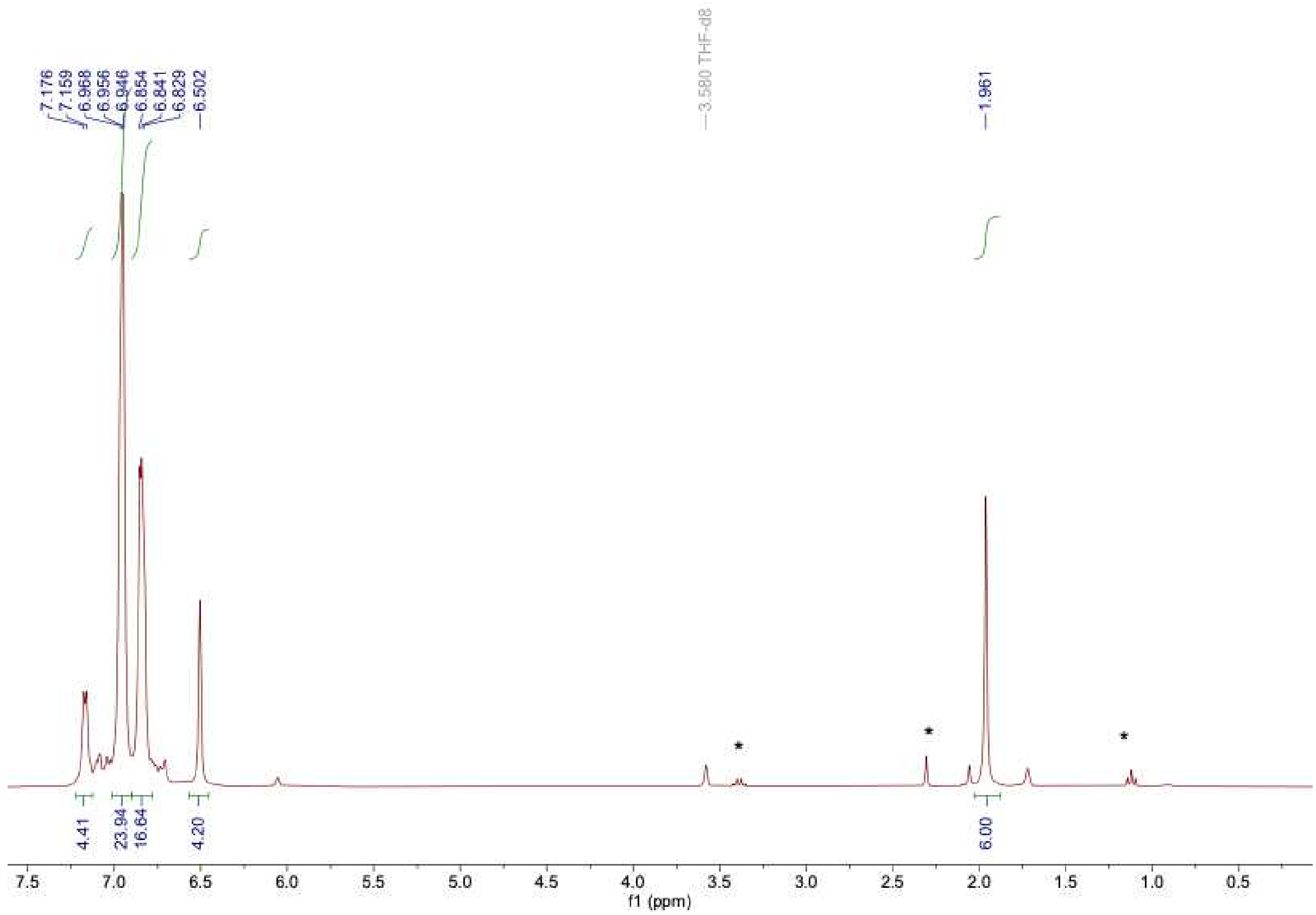

Figure S19. ^{1}H NMR spectrum of **1Rb** in d_8-THF at 298 K. *Residual solvent (toluene and diethyl ether).

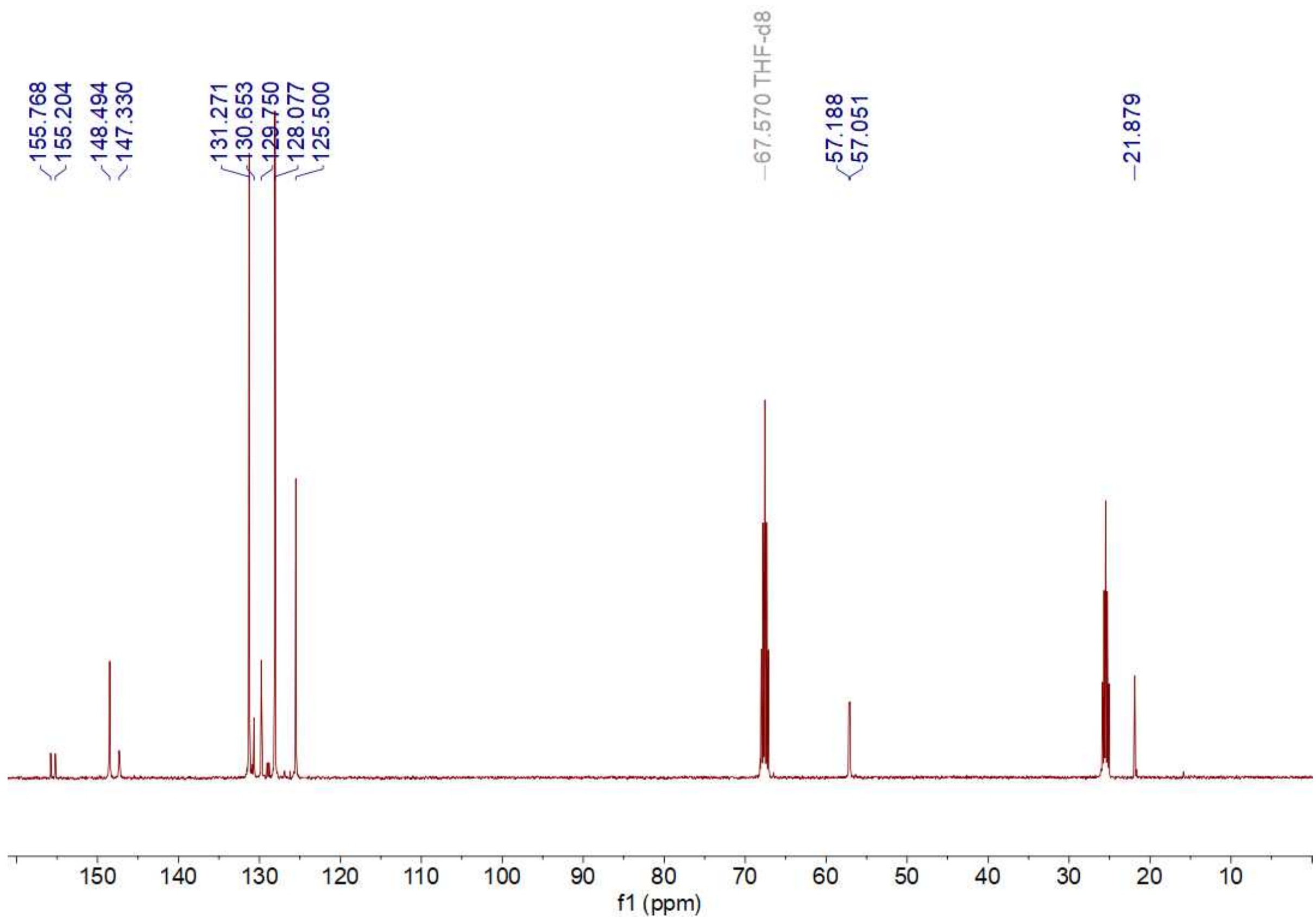

Figure S20. $^{13}C\{^{1}H\}$ NMR spectrum of **1Rb** in d_8-THF at 298 K.

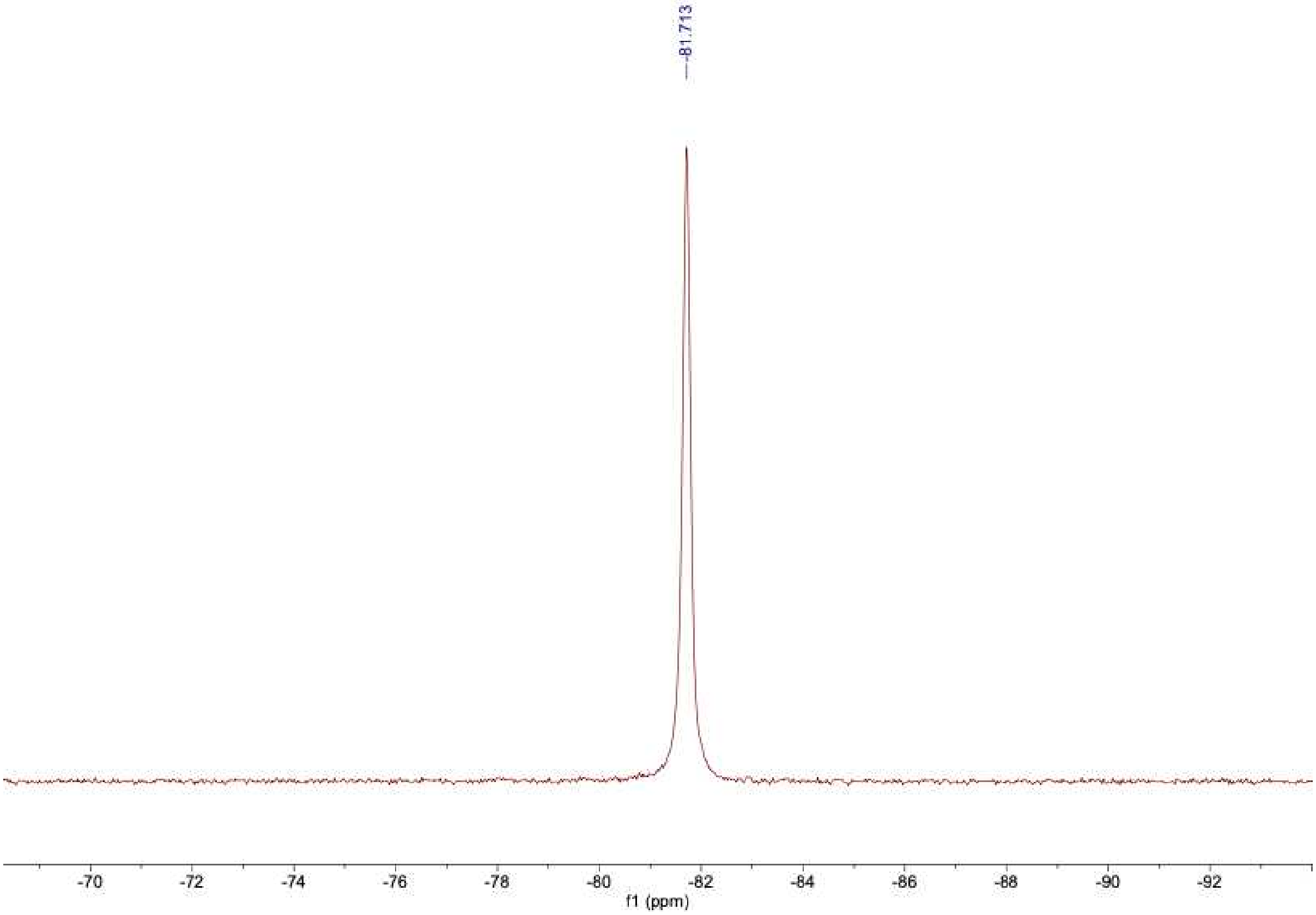

Figure S21. $^{31}P\{^{1}H\}$ NMR spectrum of **1Rb** in d_8-THF at 298 K.

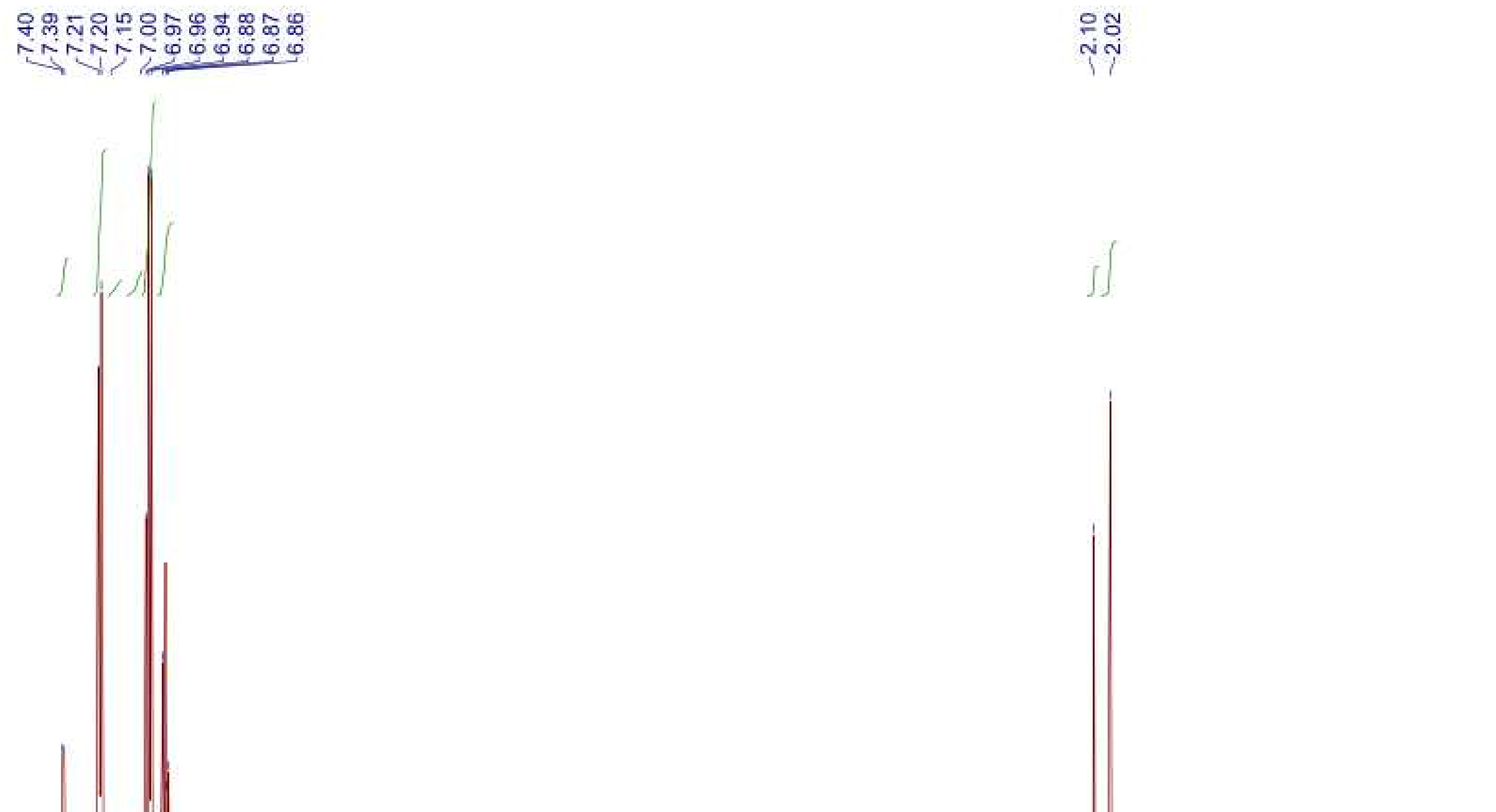

Figure S22. ^{1}H NMR spectrum of **1Cs⋅Tol** in C_6D_6 at 298 K.

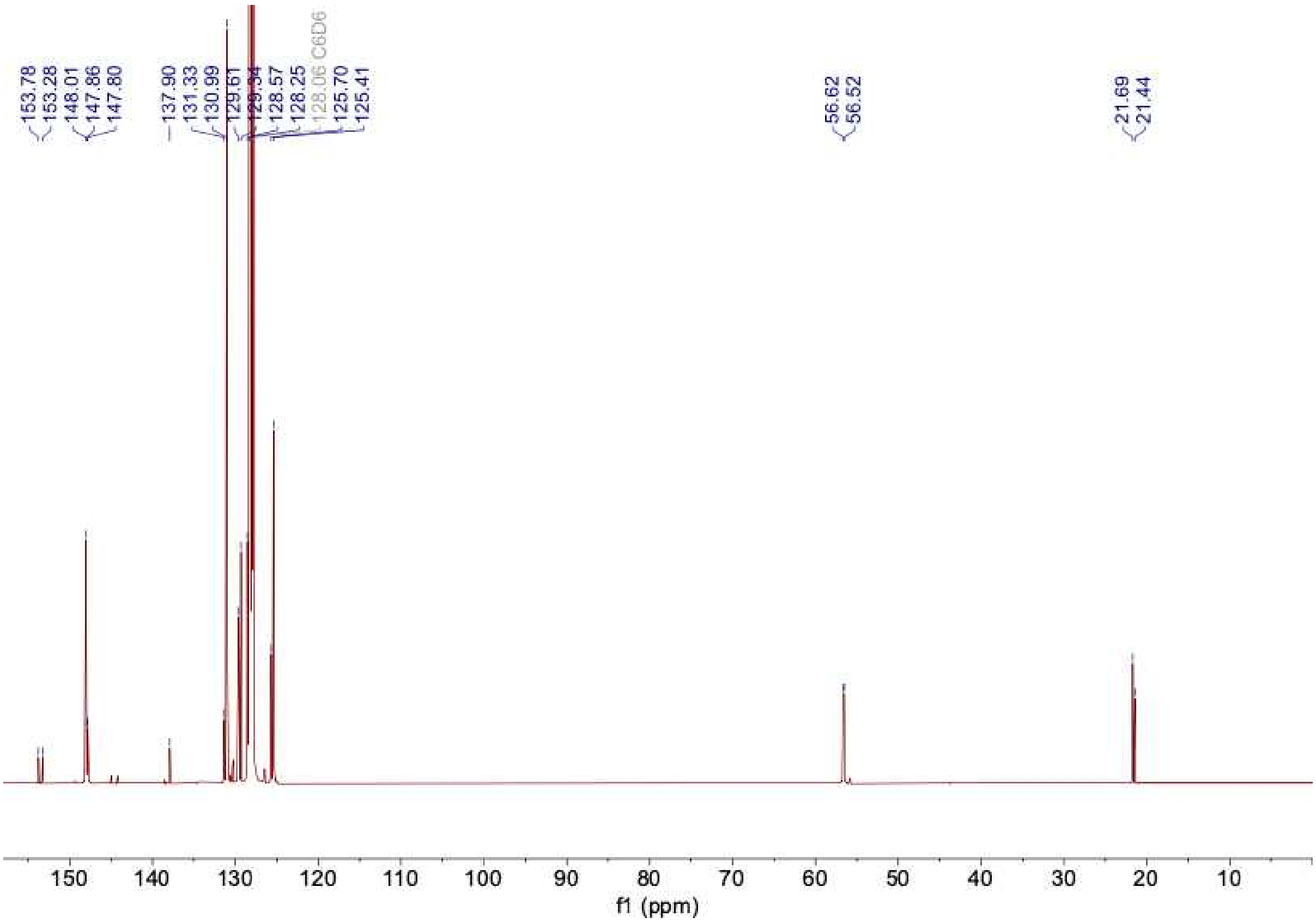

Figure S23. ^{13}C{^{1}H} NMR spectrum of **1Cs⋅Tol** in C_6D_6 at 298 K.

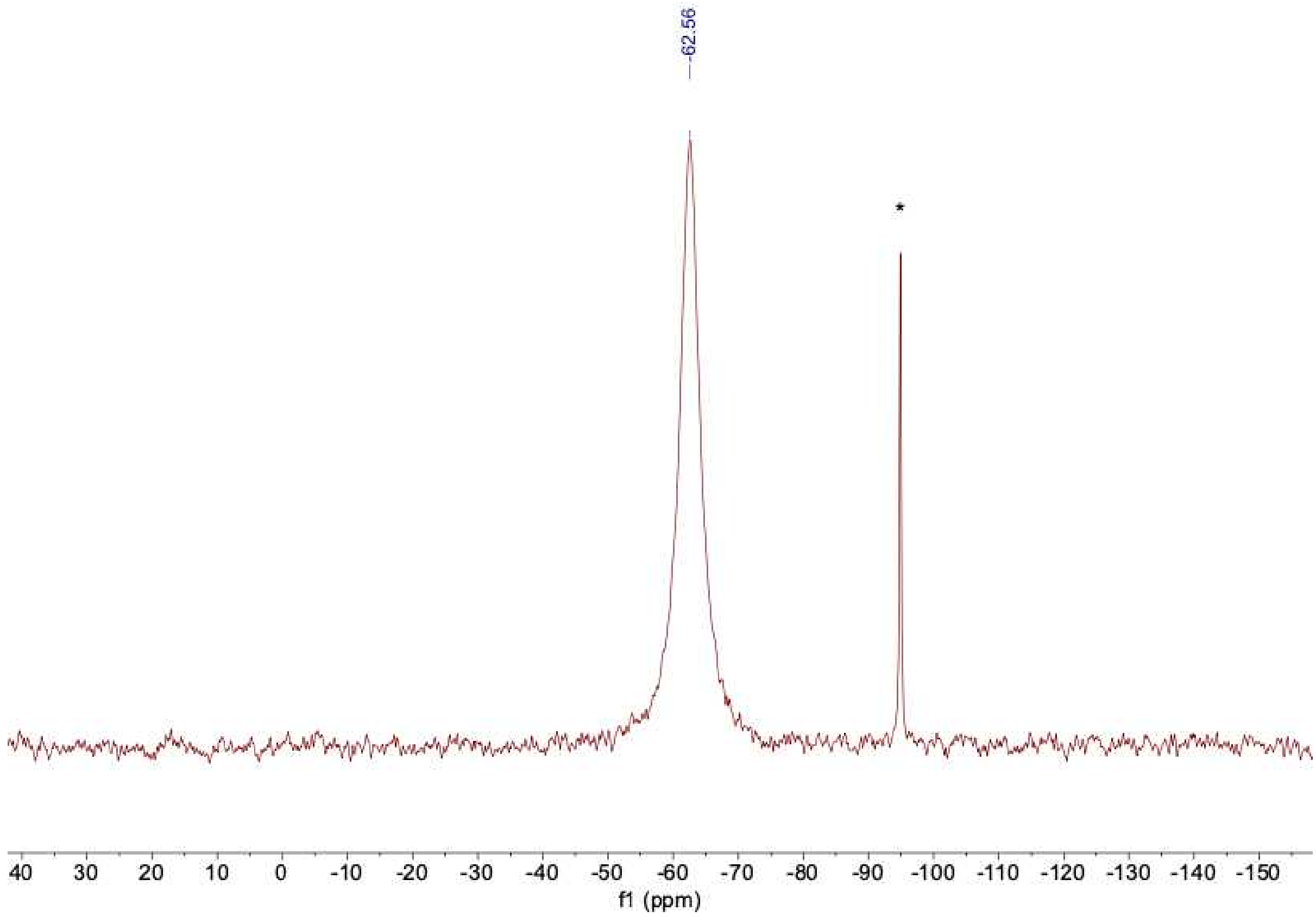

Figure S24. $^{31}P\{^{1}H\}$ NMR spectrum of **1Cs⋅Tol** in C_6D_6 at 298 K. *Residual **1H** due to hydrolysis.

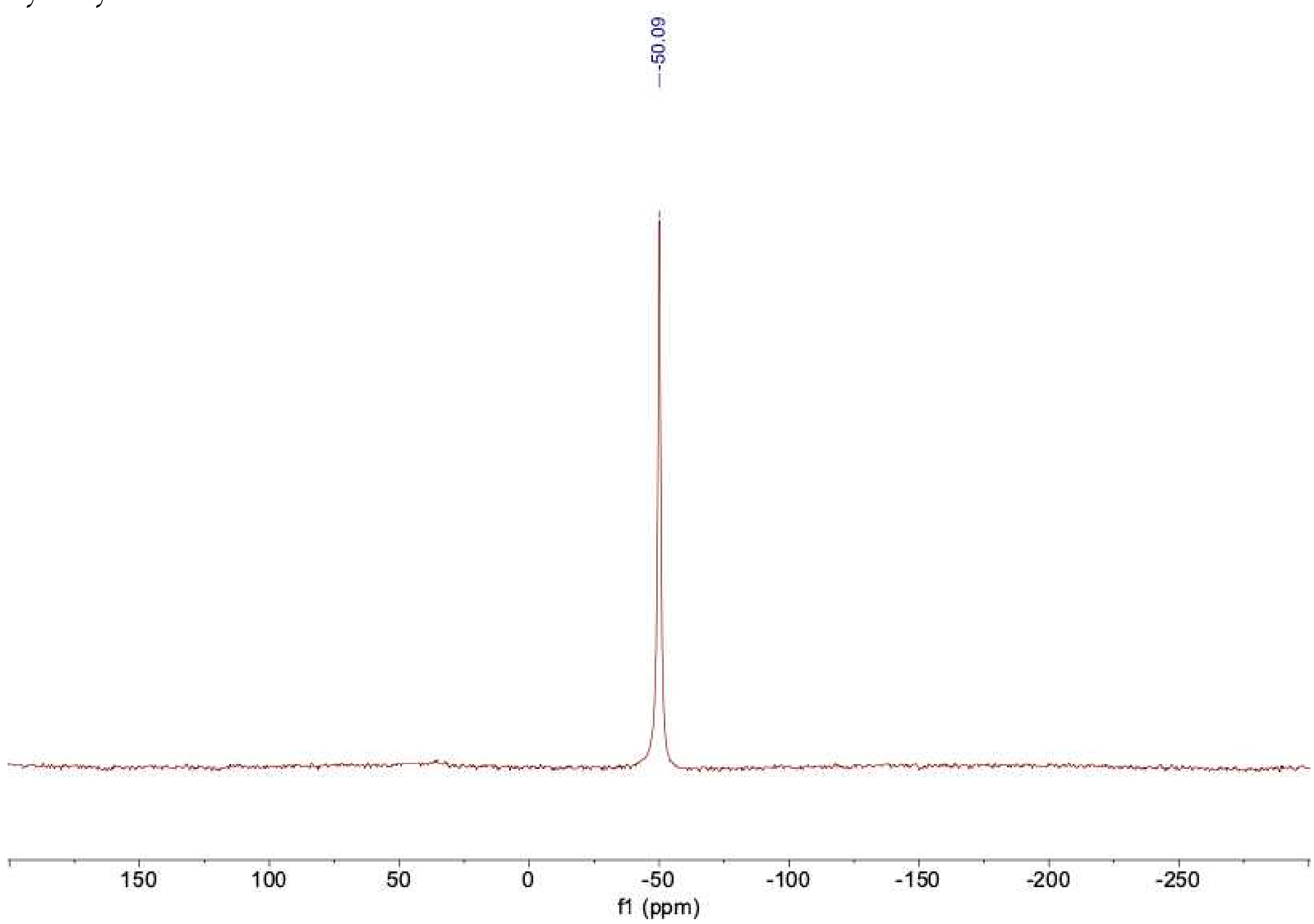

Figure S25. ^{133}Cs NMR spectrum of **1Cs⋅Tol** in C_6D_6 at 298 K.

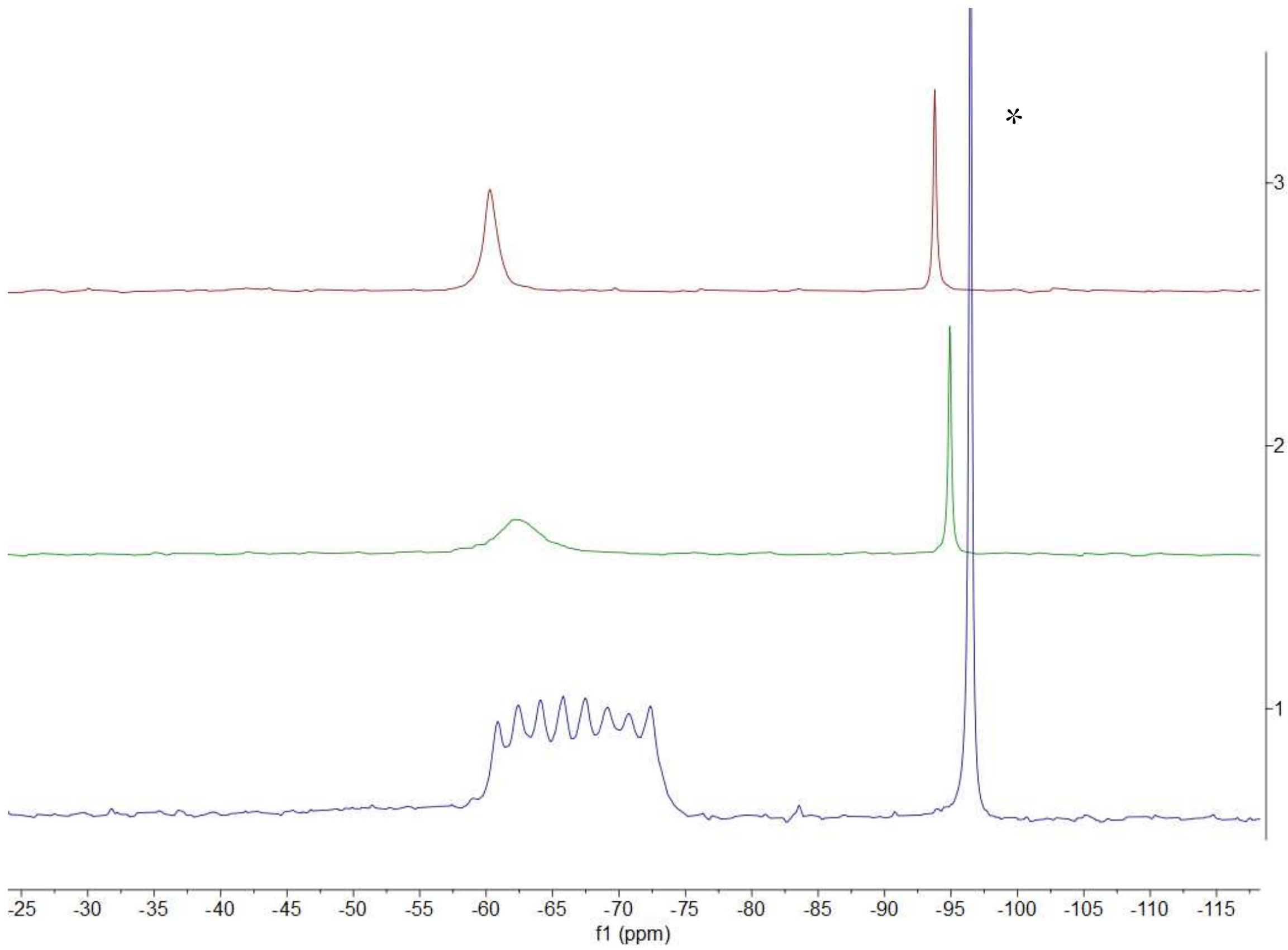

Figure S26. ^{31}P{^{1}H} NMR spectra of **1Cs□Et$_2$O** in d_8-toluene at 353 (top), 298 (middle), and 233 K (bottom). *Residual **1H** due to sample hydrolysis.

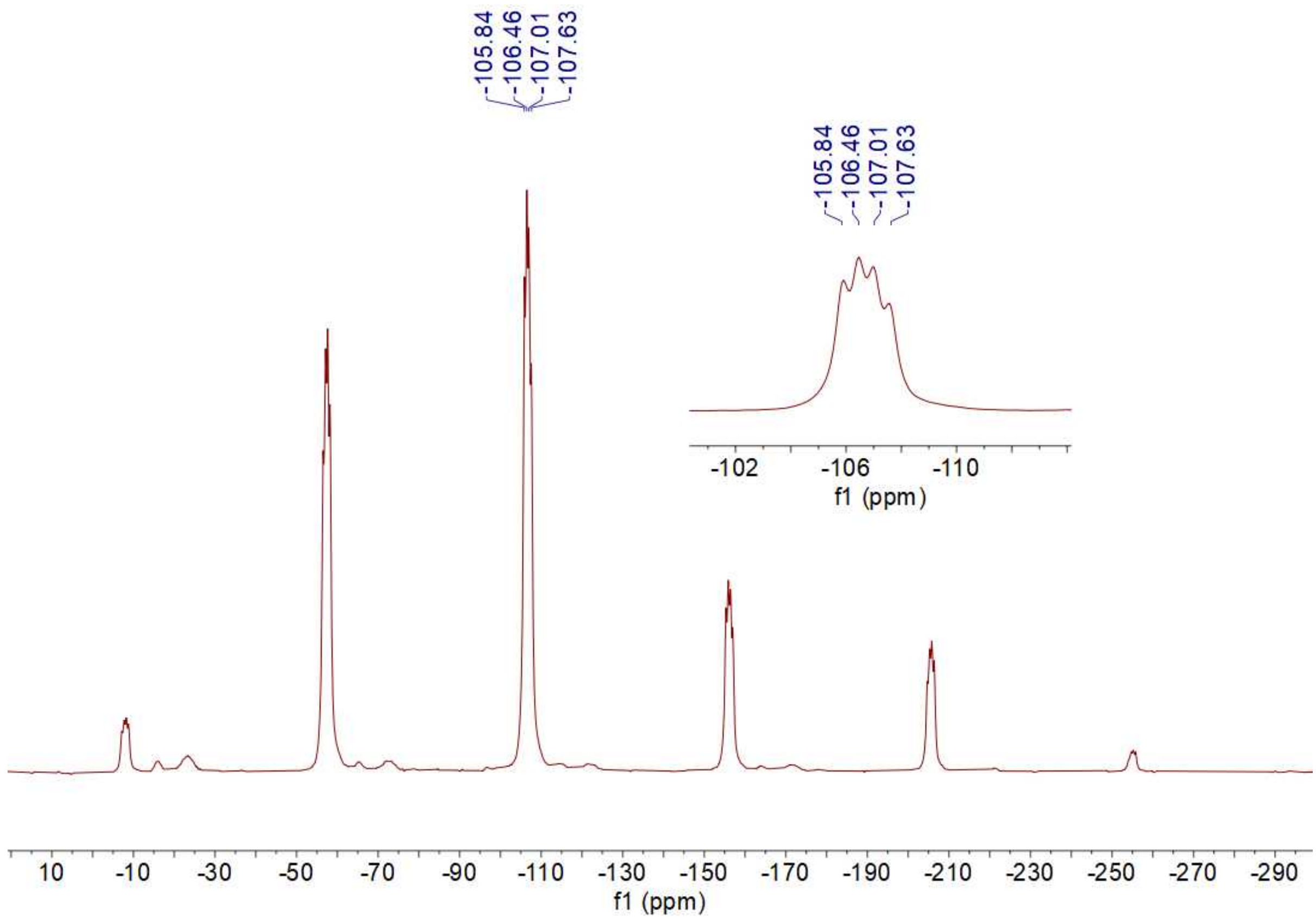

Figure S27. Solid state CP-MAS ^{31}P{^{1}H} NMR spectrum of **1Li** (spinning speed 10 kHz).

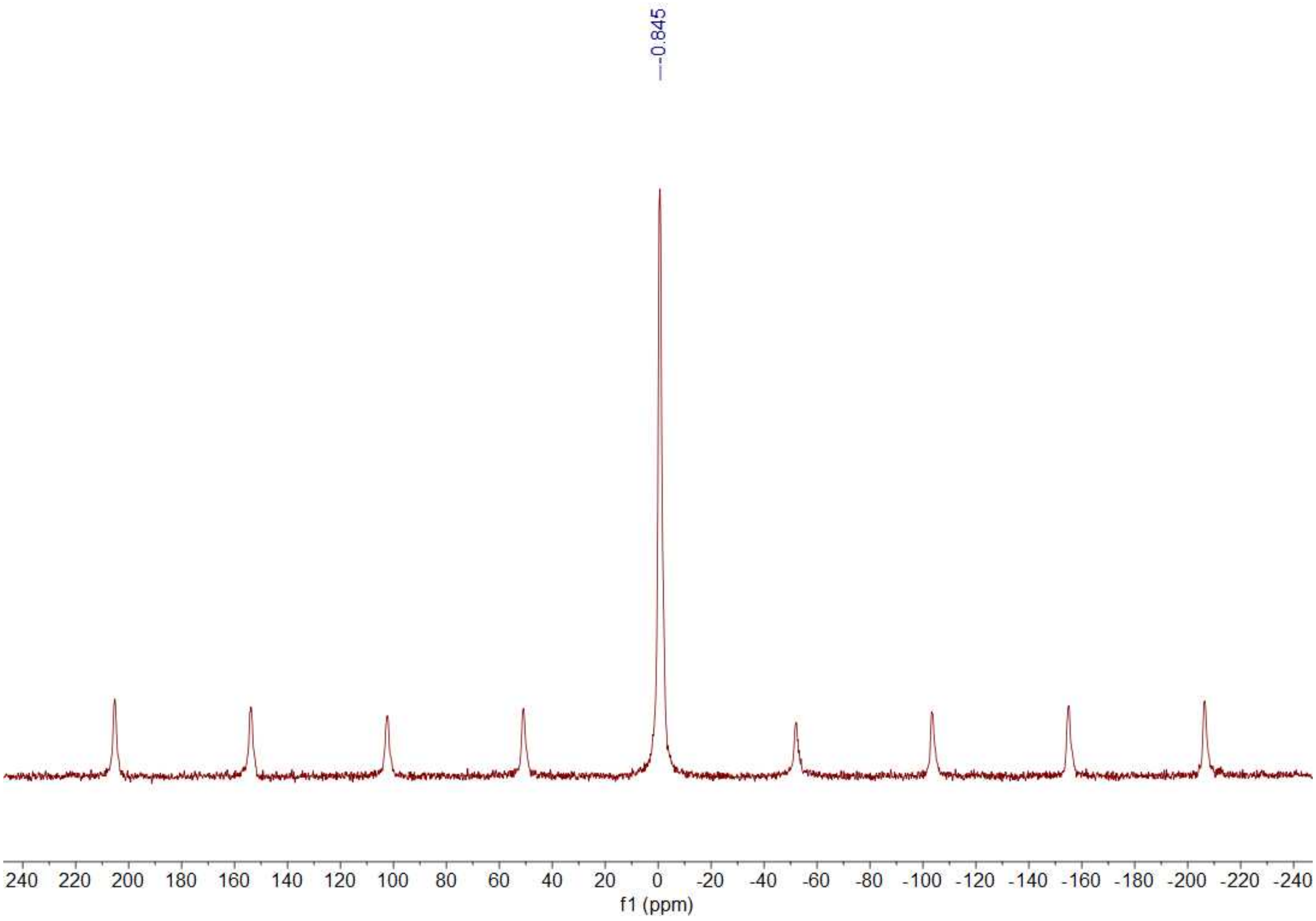

Figure S28. Solid state MAS ^{7}Li NMR spectrum of **1Li** (spinning speed 10 kHz).

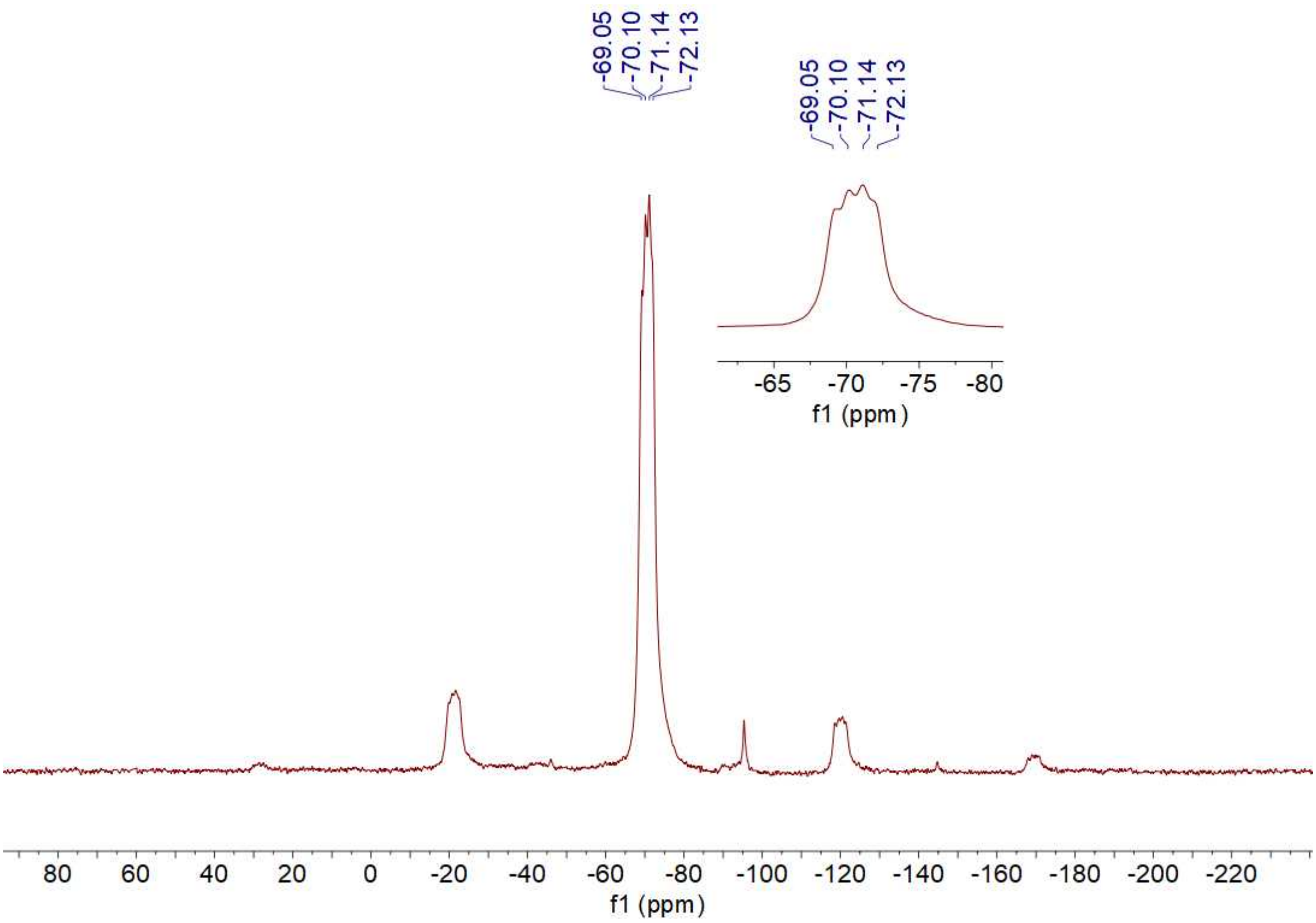

Figure S29. Solid state CP-MAS ^{31}P{^{1}H} NMR spectrum of **1Na** (spinning speed 10 kHz).

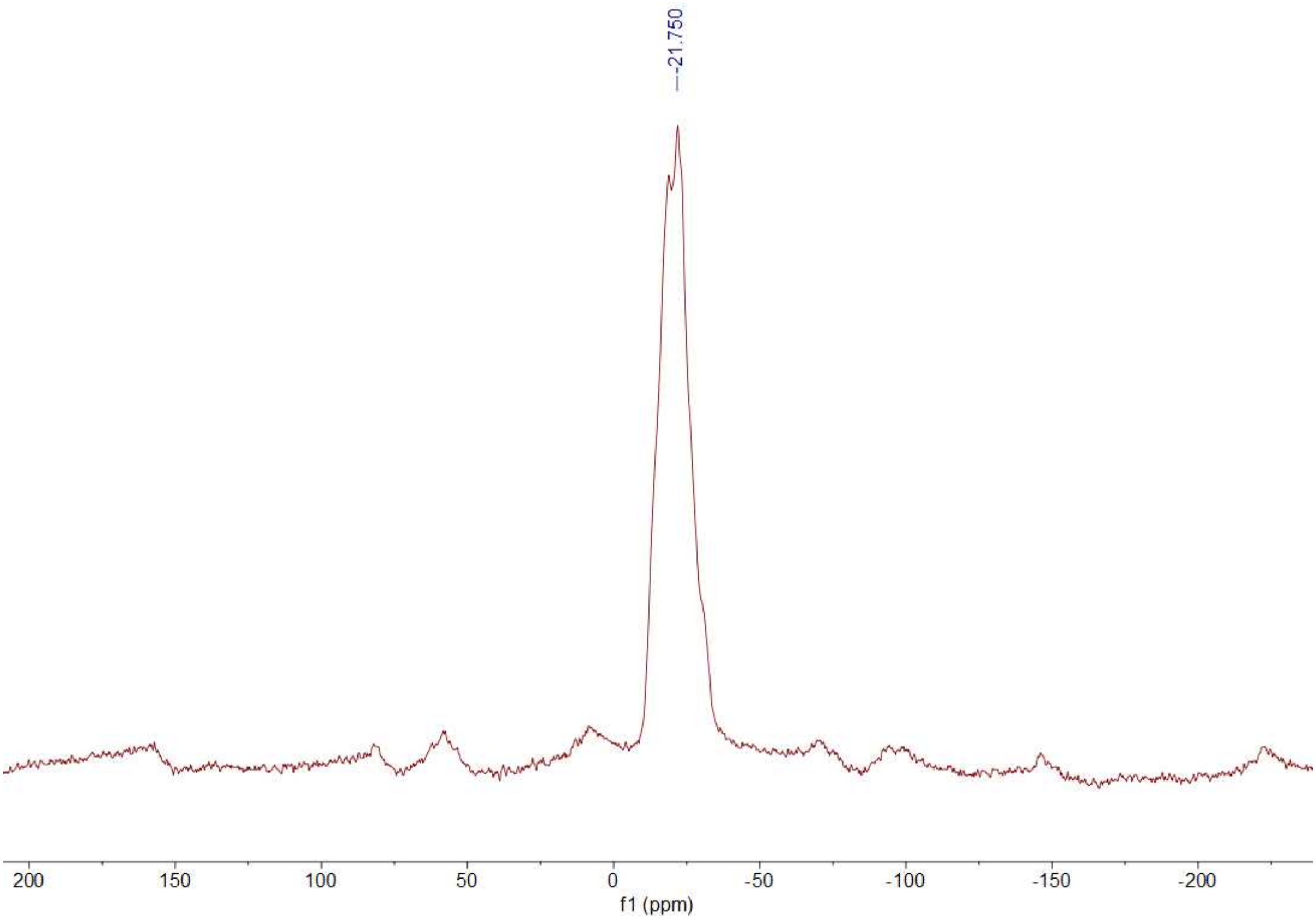

Figure S30. Solid state MAS ^{23}Na NMR spectrum of **1Na** (spinning speed 10 kHz).

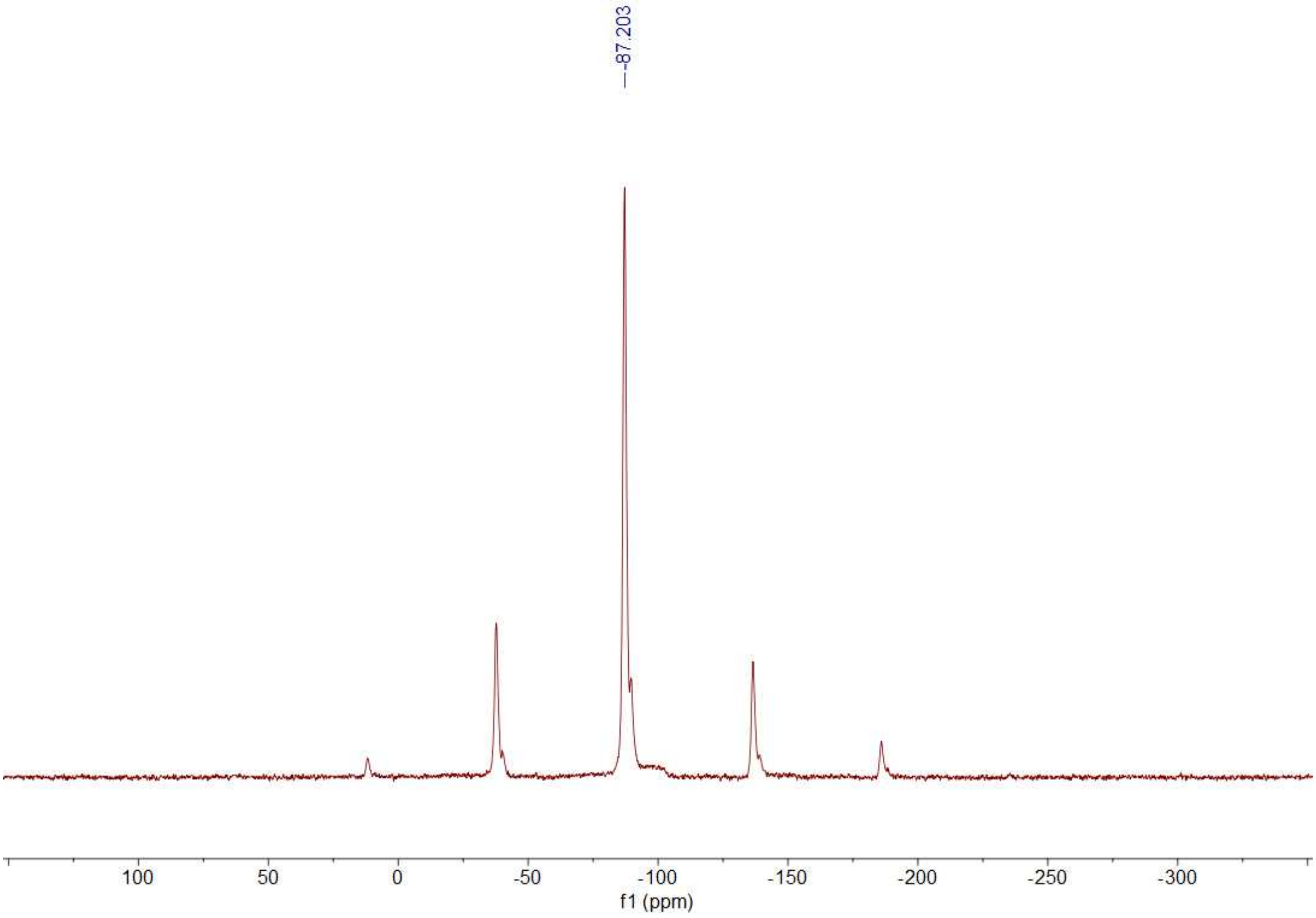

Figure S31. Solid state CP-MAS $^{31}P\{^{1}H\}$ NMR spectrum of **1K** (spinning speed 10 kHz).

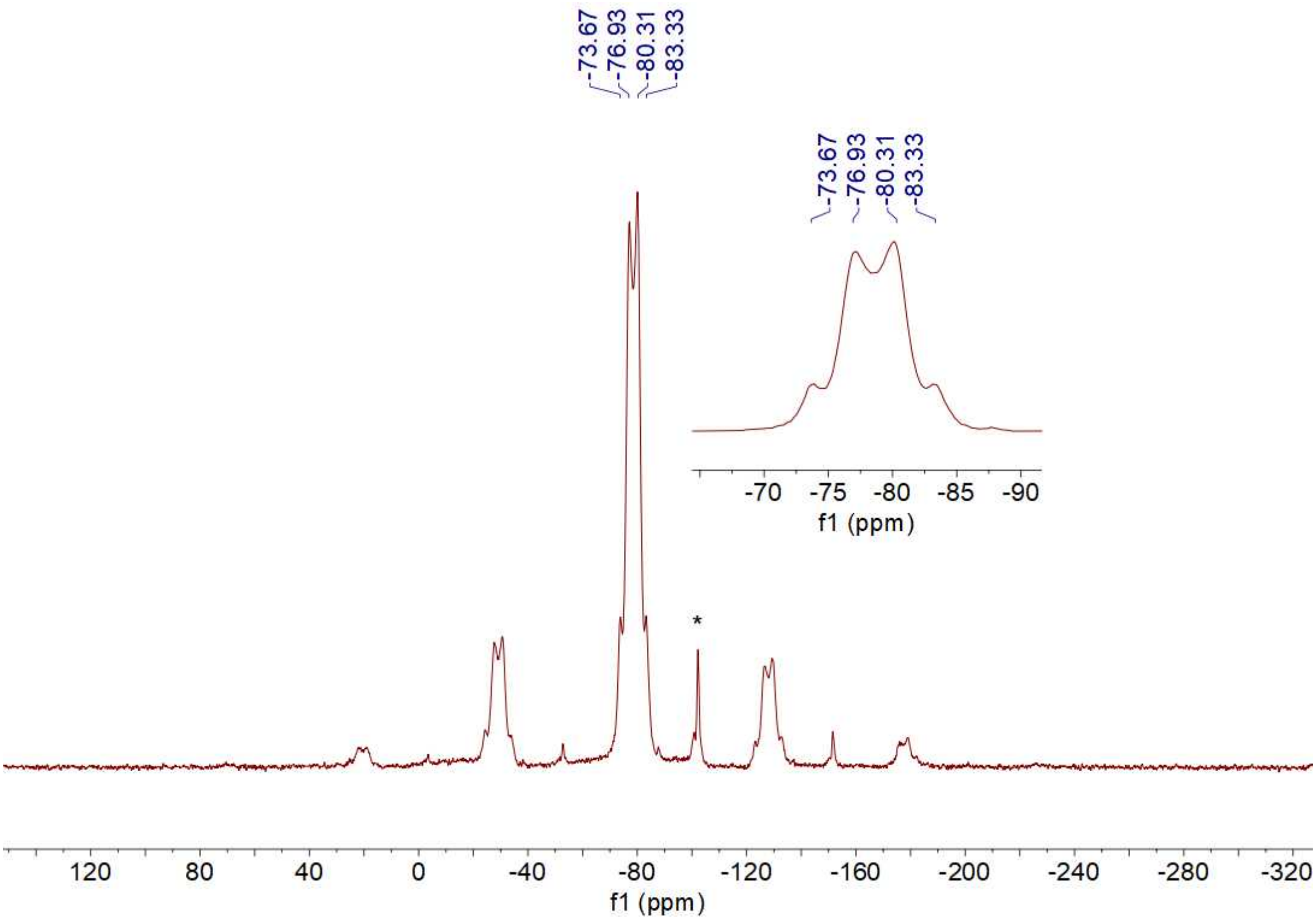

Figure S32. Solid state CP-MAS $^{31}P\{^{1}H\}$ NMR spectrum of **1Rb** (spinning speed 10 kHz). *Residual **1H** due to sample hydrolysis.

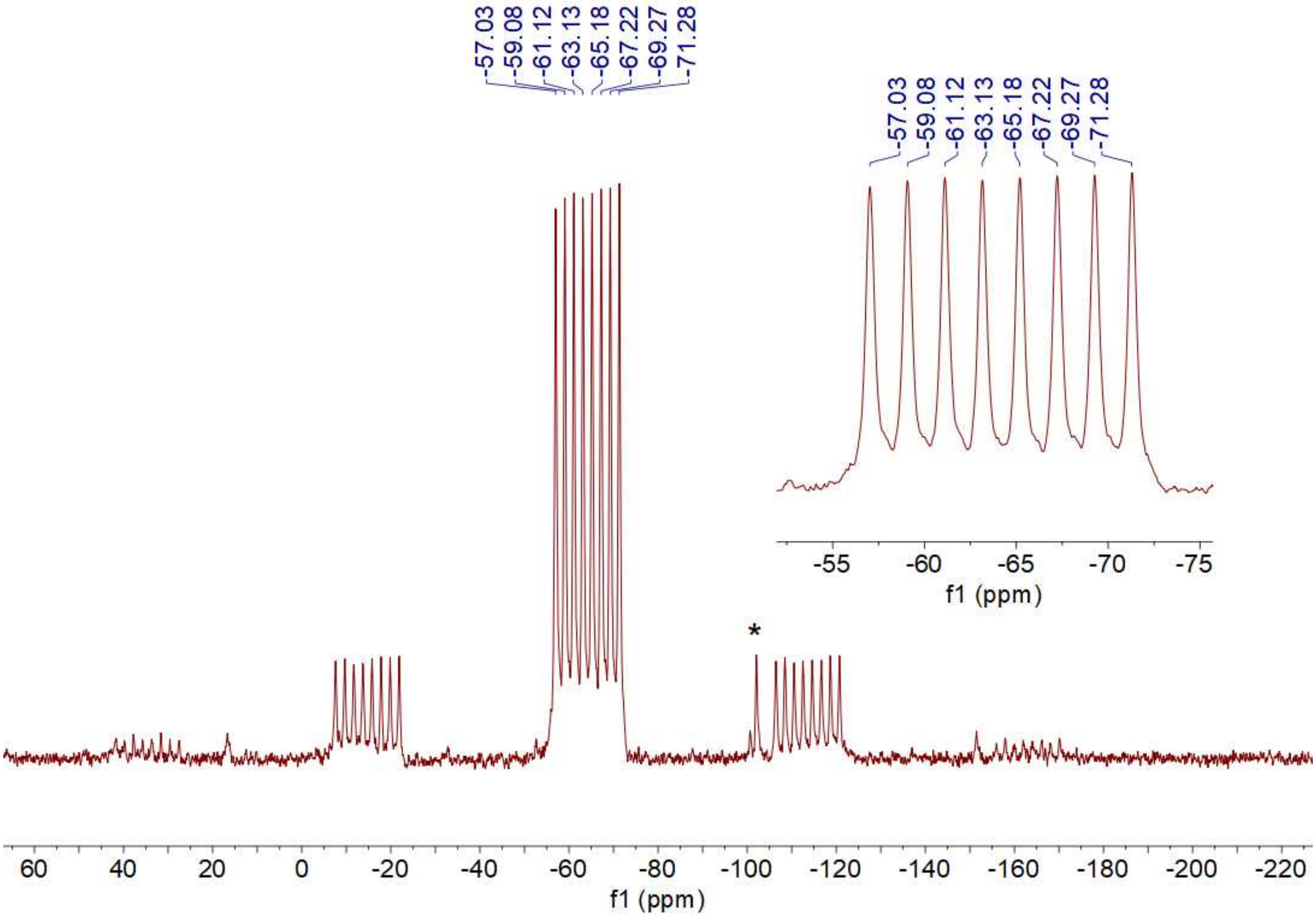

Figure S33. Solid state CP-MAS $^{31}P\{^{1}H\}$ NMR spectrum of **1Cs□Et$_2$O** (spinning speed 10 kHz). *Residual **1H** due to sample hydrolysis.

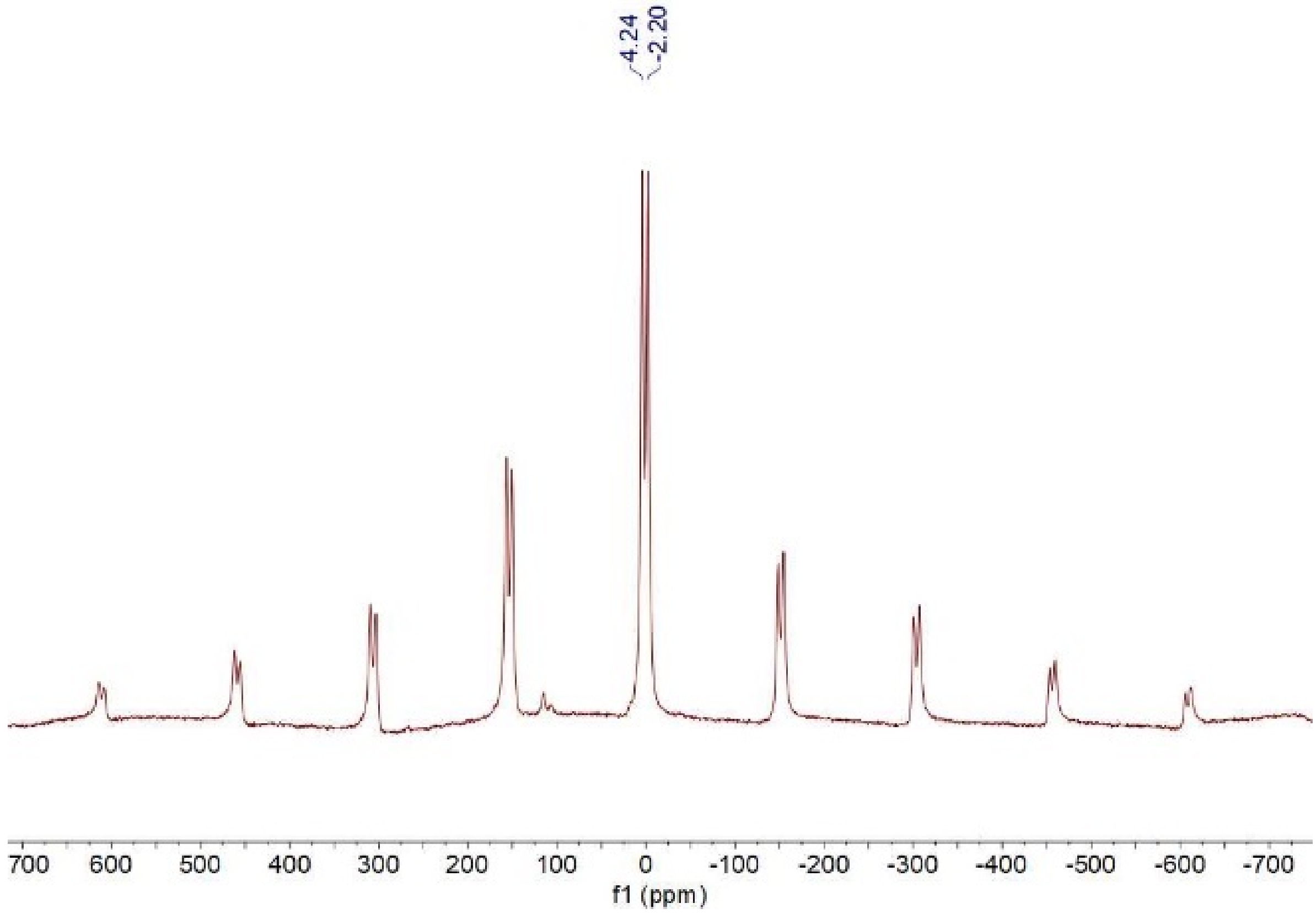

Figure S34. Solid state MAS [133]Cs NMR spectrum of **1Cs⋅Et$_2$O** (spinning speed 10 kHz).

Table S1. Crystallographic data for 1H (two polymorphs), 1H□THF, 1H□Tol, 1H⋅Ben, 1Cl, 1Li (two polymorphs), 1Na, 1K⋅Tol, 1K⋅THF, 1Rb, and 1Cs⋅Et$_2$O.

Compound	1H	1H	1H⋅THF	1H⋅Tol
formula	$C_{66}H_{55}P$	$C_{66}H_{55}P$	$C_{175}H_{166}O_2P_2$	$C_{94}H_{87}P$
M_w	879.07	879.07	2363.01	1247.60
cryst. size (mm)	0.15x0.10x0.08	0.17x0.14x0.04	0.16x0.11x0.05	0.15x0.12x0.07
cryst. syst.	Monoclinic	Monoclinic	Triclinic	Triclinic
space group	$P2_1/c$	$P2_1/c$	*P*-1	*P*-1
a (Å)	18.7237(2)	12.42550(10)	13.9092(2)	14.1736(2)
b (Å)	18.1284(2)	13.9759(2)	14.4910(2)	14.8539(2)
c (Å)	14.2140(2)	28.4306(2)	19.0829(2)	19.2755(2)
α (deg)			108.760(10)	69.9650(10)
β (deg)	99.9410(10)	92.0190(10)	99.3670(10)	82.7990(10)

γ (deg)			107.3870(10)	68.4260(10)
V (Å^3)	4752.23(10)	4934.12(9)	3329.90(9)	3545.46(8)
Z	4	4	1	2
μ (mm^{-1})	0.829	0.798	0.727	0.699
reflns. measd.	62468	47031	63048	45199
unique reflns.	9467	9761	13258	14165
R_{int}	0.032	0.026	0.033	0.026
refined params.	613	610	937	932
R (on F, $F^2>2\sigma$)	0.035	0.036	0.056	0.045
R_w (on F^2, all data)	0.090	0.096	0.168	0.125
goodness of fit	1.060	1.042	1.046	1.051
max, min electron density (e Å^{-3})	0.31/-0.70	0.30/-0.42	0.87/-0.47	1.16/-0.41

Compound	**1H·Ben**	**1Cl**	**1Li**	**1Li**
formula	$C_{90}H_{79}P$	$C_{80}H_{82}ClP$	$C_{155}H_{148}Li_2O_4P_2$	$C_{155}H_{148}Li_2O_4P_2$
M_w	1191.50	1109.87	2150.55	2150.55
cryst. size (mm)	0.17x0.12x0.10	0.10x0.07x0.04	0.15x0.10x0.09	0.15x0.11x0.09
cryst. syst.	Triclinic	Triclinic	Monoclinic	Monoclinic
space group	*P*-1	*P*-1	$P2_1/c$	$P2_1/c$
a (Å)	14.57450(10)	13.9041(2)	19.1573(3)	29.6421(2)
b (Å)	14.7039(2)	14.0141(2)	18.3551(4)	18.36660(10)
c (Å)	18.32110(10)	17.5059(2)	17.9202(3)	56.6428(3)
α (deg)	94.5020(10)	109.7540(10)		
β (deg)	95.6340(10)	101.5170(10)	106.211(2)	101.1840(10)
γ (deg)	118.3260(10)	96.5170(10)		
V ($Å^3$)	3404.19(6)	3081.57(8)	6050.8(2)	30252.1(3)
Z	2	2	2	2
μ (mm^{-1})	0.706	1.127	0.761	0.761
reflns. measd.	64681	47198	112616	325241
unique reflns.	13707	12242	12213	63913
R_{int}	0.031	0.031	0.038	0.026
refined params.	990	885	931	4039
R (on *F*, $F^2>2\sigma$)	0.040	0.045	0.047	0.048
R_w (on F^2, all data)	0.107	0.133	0.131	0.144
goodness of fit	1.040	1.060	1.039	1.0338
max, min electron density (e $Å^{-3}$)	0.23/-0.30	0.37/-0.54	0.34/-0.27	0.32/-0.30

Compound	**1Na**	**1K□Tol**	**1K□THF**	**1Rb**	**1Cs□Et$_2$O**
formula	$C_{70}H_{64}NaOP$	$C_{181}H_{164}K_2P_2$	$C_{92}H_{94}KO_3P$	$C_{66}H_{54}PRb$	$C_{74}H_{74}CsO_2P$
M_w	975.17	2479.25	1317.74	963.53	1159.21
cryst. size (mm)	0.21x0.03x0.02	0.31x0.10x0.02	0.20x0.11x0.07	0.1x0.04x0.02	0.10x0.07x0.03
cryst. syst.	Monoclinic	Monoclinic	Monoclinic	Monoclinic	Triclinic
space group	$P2_1/n$	$I2/a$	$P2_1/n$	$P2_1/c$	P-1
a (Å)	13.4847(3)	26.7175(5)	14.9127(2)	10.1613(2)	10.4534(2)
b (Å)	11.1981(3)	19.8189(3)	24.7329(3)	23.0947(3)	14.0867(2)
c (Å)	36.6360(8)	26.4471(4)	20.9886(3)	21.2323(3)	21.8231(4)
α (deg)					71.386(2)
β (deg)	97.634(2)	98.496(2)	105.2570(10)	95.9480(10)	85.0970(10)
γ (deg)					86.0980(10)
V (Å^3)	5483.1(2)	13850.4(4)	7472.98(18)	4955.81(14)	3031.55(10)
Z	4	4	4	4	2
μ (mm^{-1})	0.851	1.240	1.206	1.989	5.356
reflns. measd.	41805	53706	109758	66184	81135
unique reflns.	10844	13555	15004	10141	11825
R_{int}	0.028	0.026	0.033	0.037	0.041
refined params.	662	1054	1030	615	766
R (on F, $F^2>2\sigma$)	0..043	0.039	0.047	0.041	0.026
R_w (on F^2, all data)	0.112	0.103	0.1387	0.110	0.067
goodness of fit	1.044	1.035	1.055	1.038	1.054
max, min electron	0.24/-0.31	0.37/-0.32	0.43/-0.57	1.39/-0.78	0.46/-0.60

density (e Å^{-3})

Converged geometry, atomic coordinates, final electronic energy, and final Gibbs free energy of **1Na$_{pyr}$**.

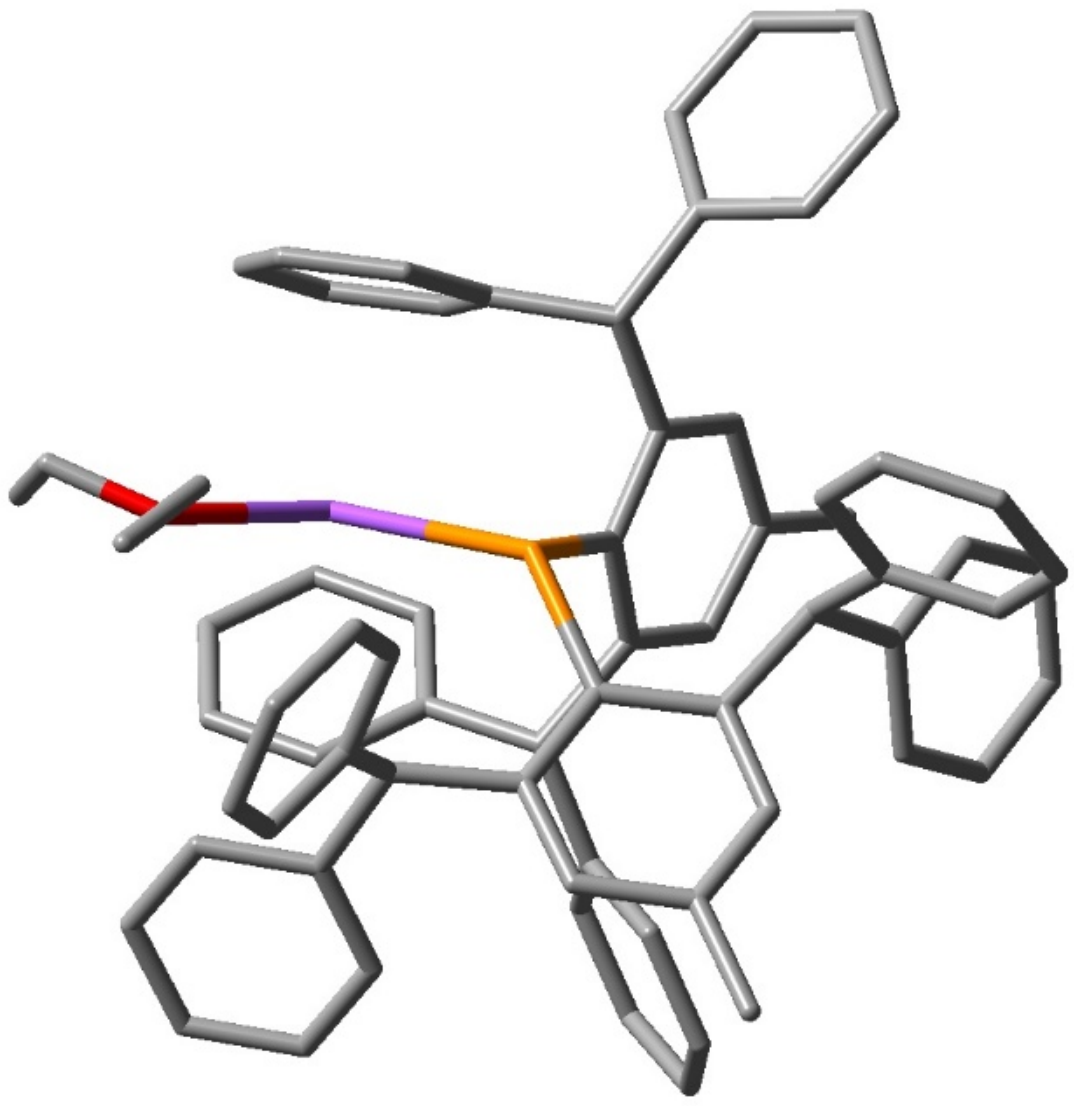

Center Number	Atomic Number	Atomic Type	Coordinates (Angstroms) X	Y	Z
1	15	0	-0.000563	0.358419	0.925867
2	11	0	-1.490047	2.518251	-0.378515
3	8	0	-3.460031	2.700712	1.155303
4	6	0	1.119361	0.695934	-0.516238
5	6	0	0.953070	0.089420	-1.794407
6	6	0	1.945019	0.199345	-2.770615
7	1	0	1.801508	-0.304695	-3.725289
8	6	0	3.082398	0.992643	-2.582666
9	6	0	3.166920	1.728692	-1.395115
10	1	0	4.004227	2.409743	-1.249843
11	6	0	2.216241	1.593334	-0.380671
12	6	0	-0.441506	-0.377866	-2.212012
13	1	0	-0.950259	-0.768493	-1.328908
14	6	0	-1.255411	0.859135	-2.622170
15	6	0	-2.653869	0.818829	-2.488592
16	1	0	-3.128040	-0.106493	-2.185531
17	6	0	-3.436570	1.951853	-2.718455
18	1	0	-4.513209	1.888640	-2.577189
19	6	0	-2.837806	3.155451	-3.109816

20	1	0	-3.444203	4.042654	-3.280916
21	6	0	-1.451095	3.201878	-3.280984
22	1	0	-0.971186	4.125835	-3.598114
23	6	0	-0.666736	2.066550	-3.037697
24	1	0	0.412525	2.130344	-3.138372
25	6	0	-0.467019	-1.508442	-3.224883
26	6	0	-0.548572	-1.293834	-4.606162
27	1	0	-0.626303	-0.275832	-4.984709
28	6	0	-0.539816	-2.374388	-5.496258
29	1	0	-0.603841	-2.192790	-6.568306
30	6	0	-0.452141	-3.682642	-5.012040
31	1	0	-0.447755	-4.523406	-5.704030
32	6	0	-0.376908	-3.905228	-3.630970
33	1	0	-0.314125	-4.921147	-3.242864
34	6	0	-0.386323	-2.826237	-2.745882
35	1	0	-0.329871	-2.995243	-1.672060
36	6	0	4.152140	1.085797	-3.642541
37	1	0	4.838936	0.230827	-3.569688
38	1	0	4.742011	2.005361	-3.531500
39	1	0	3.715026	1.070367	-4.650799
40	6	0	2.236362	2.501120	0.845622
41	1	0	1.953009	1.861760	1.699726
42	6	0	1.109128	3.523079	0.701506
43	6	0	0.882284	4.193829	-0.512406
44	1	0	1.534838	3.982781	-1.356312
45	6	0	-0.183408	5.089745	-0.652229
46	1	0	-0.341023	5.600034	-1.599958
47	6	0	-1.051455	5.321884	0.422201
48	1	0	-1.885601	6.011983	0.316009
49	6	0	-0.839314	4.652777	1.632484
50	1	0	-1.516245	4.815484	2.467895
51	6	0	0.228975	3.760771	1.768096
52	1	0	0.373890	3.215113	2.697820
53	6	0	3.599538	3.094680	1.163562
54	6	0	3.966238	4.410257	0.855571
55	1	0	3.239201	5.079931	0.400858
56	6	0	5.258887	4.873479	1.130786
57	1	0	5.527444	5.899974	0.884790
58	6	0	6.200824	4.025491	1.718132
59	1	0	7.205428	4.386286	1.932630
60	6	0	5.841540	2.709324	2.035180
61	1	0	6.565121	2.040986	2.499533

62	6	0	4.552753	2.251591	1.760096
63	1	0	4.272161	1.228005	2.006690
64	6	0	-0.163999	-1.495575	0.801722
65	6	0	-1.434897	-2.127872	0.878624
66	6	0	-1.545420	-3.523984	0.920059
67	1	0	-2.535432	-3.967924	1.023404
68	6	0	-0.428418	-4.353513	0.840674
69	6	0	0.826050	-3.736047	0.786359
70	1	0	1.720912	-4.357490	0.794161
71	6	0	0.981977	-2.347527	0.781530
72	6	0	-2.716835	-1.311218	1.009402
73	1	0	-2.395993	-0.255988	0.952638
74	6	0	-3.729438	-1.464908	-0.122165
75	6	0	-4.789419	-0.539957	-0.178088
76	1	0	-4.864827	0.220354	0.595733
77	6	0	-5.710191	-0.554647	-1.225589
78	1	0	-6.518771	0.175502	-1.245245
79	6	0	-5.584534	-1.492650	-2.258070
80	1	0	-6.292072	-1.498731	-3.085486
81	6	0	-4.532518	-2.410215	-2.220947
82	1	0	-4.404247	-3.130054	-3.027443
83	6	0	-3.617620	-2.399098	-1.160797
84	1	0	-2.787824	-3.097341	-1.173012
85	6	0	-3.290472	-1.503548	2.414695
86	6	0	-2.484156	-1.141030	3.508948
87	1	0	-1.484482	-0.751196	3.320829
88	6	0	-2.942804	-1.288837	4.816950
89	1	0	-2.301884	-1.001196	5.649496
90	6	0	-4.220699	-1.809537	5.060973
91	1	0	-4.580906	-1.926656	6.081789
92	6	0	-5.023016	-2.187056	3.982121
93	1	0	-6.013563	-2.604521	4.158582
94	6	0	-4.560016	-2.037530	2.668721
95	1	0	-5.194899	-2.331178	1.835869
96	6	0	-0.556996	-5.859238	0.832929
97	1	0	-0.434250	-6.263787	-0.183992
98	1	0	-1.542210	-6.174002	1.200015
99	1	0	0.213670	-6.324891	1.462232
100	6	0	2.413367	-1.808579	0.865874
101	1	0	2.334137	-0.727761	1.016857
102	6	0	3.274842	-2.003846	-0.378268
103	6	0	2.911133	-2.804567	-1.466020

104	1	0	1.948370	-3.303681	-1.469138
105	6	0	3.759812	-2.949729	-2.568934
106	1	0	3.442848	-3.565712	-3.409312
107	6	0	4.998307	-2.305731	-2.594334
108	1	0	5.663881	-2.423921	-3.448304
109	6	0	5.371157	-1.494888	-1.515204
110	1	0	6.325826	-0.970614	-1.528553
111	6	0	4.513249	-1.341129	-0.427432
112	1	0	4.803196	-0.701133	0.404012
113	6	0	3.076103	-2.343249	2.139072
114	6	0	4.001591	-3.394531	2.137639
115	1	0	4.310178	-3.831764	1.190074
116	6	0	4.540749	-3.869634	3.338540
117	1	0	5.259277	-4.688314	3.320986
118	6	0	4.164924	-3.294738	4.555984
119	1	0	4.588273	-3.661955	5.489761
120	6	0	3.243482	-2.240293	4.565764
121	1	0	2.947035	-1.783510	5.509395
122	6	0	2.703307	-1.772036	3.366726
123	1	0	1.975923	-0.959861	3.363149
124	6	0	-3.170264	2.203468	2.484524
125	1	0	-2.704304	3.016361	3.066715
126	1	0	-2.418364	1.422746	2.329125
127	6	0	-4.389842	1.655997	3.220361
128	1	0	-4.872276	0.858474	2.645953
129	1	0	-5.124486	2.441031	3.440621
130	1	0	-4.060701	1.214555	4.168219
131	6	0	-4.266422	3.884198	1.163210
132	1	0	-3.763164	4.660717	1.767000
133	1	0	-5.239585	3.672035	1.630546
134	6	0	-4.461816	4.350252	-0.270071
135	1	0	-4.964730	3.575353	-0.859291
136	1	0	-3.498581	4.572627	-0.744930
137	1	0	-5.074376	5.261296	-0.286226

Final electronic energy: -32882.62157346 au
Final Gibbs free energy: -2059886.3 kcal mol^{-1}

Converged geometry, atomic coordinates, final electronic energy, and final Gibbs free energy of **1Na**$_{plan}$.

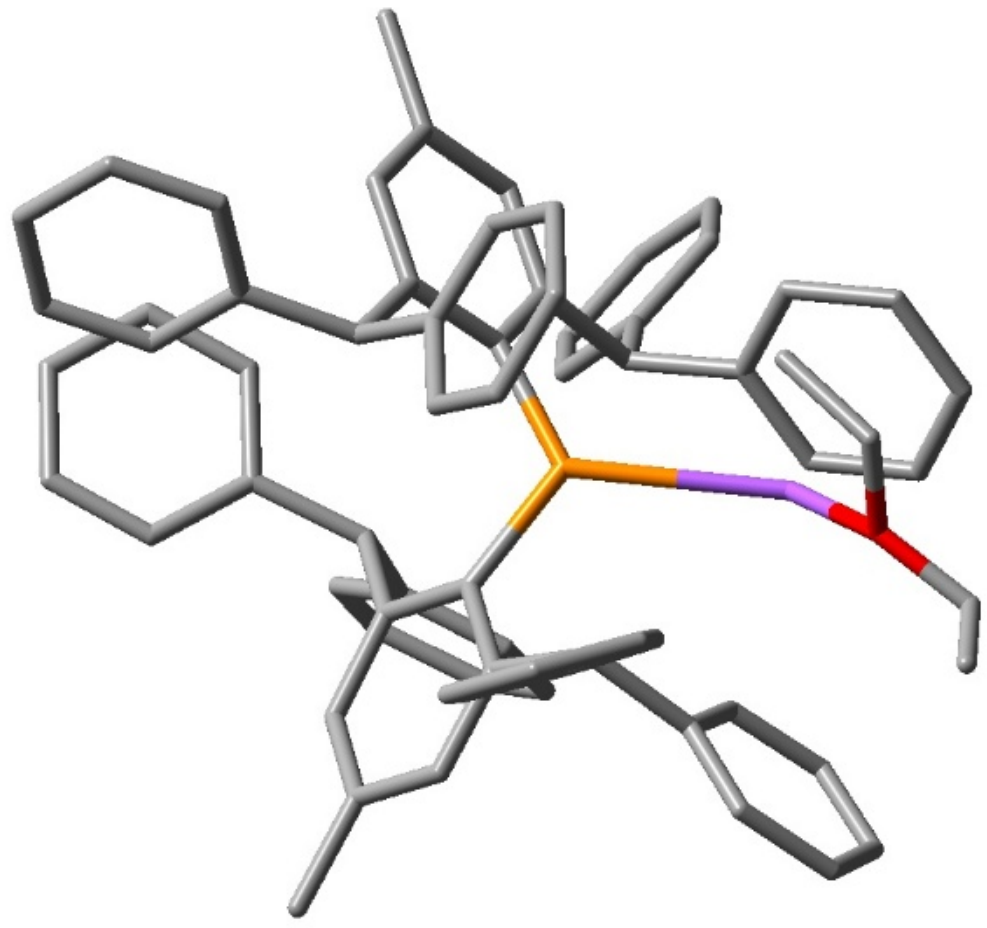

Center Number	Atomic Number	Atomic Type	Coordinates (Angstroms) X	Y	Z
1	15	0	0.291931	-0.245283	-0.247040
2	11	0	2.136171	-2.145754	-1.087918
3	8	0	4.428106	-1.716721	-1.442320
4	6	0	-1.246973	0.165698	-1.161690
5	6	0	-1.585907	1.495661	-1.536250
6	6	0	-2.651233	1.725976	-2.408714
7	1	0	-2.907823	2.750853	-2.670820
8	6	0	-3.408345	0.677796	-2.945414
9	6	0	-3.100061	-0.627846	-2.553083
10	1	0	-3.718353	-1.445443	-2.918774
11	6	0	-2.054742	-0.899389	-1.661382
12	6	0	-0.747630	2.668184	-1.015430
13	1	0	-0.508182	2.447066	0.031720
14	6	0	0.585221	2.786981	-1.749850
15	6	0	1.719929	3.201185	-1.040751
16	1	0	1.648038	3.342912	0.035353
17	6	0	2.936241	3.417104	-1.689693
18	1	0	3.805894	3.718332	-1.111000
19	6	0	3.030154	3.234200	-3.074444
20	1	0	3.974881	3.411131	-3.587570
21	6	0	1.904624	2.817454	-3.793183
22	1	0	1.968464	2.671000	-4.871406
23	6	0	0.692972	2.586904	-3.133119

24	1	0	-0.180232	2.249989	-3.689158
25	6	0	-1.504035	3.999577	-1.005028
26	6	0	-1.426337	4.927885	-2.053677
27	1	0	-0.776116	4.726629	-2.901748
28	6	0	-2.164118	6.115801	-2.012938
29	1	0	-2.088256	6.825520	-2.836002
30	6	0	-2.989232	6.395475	-0.919665
31	1	0	-3.560183	7.322449	-0.885484
32	6	0	-3.070660	5.475979	0.131353
33	1	0	-3.710352	5.674245	0.989328
34	6	0	-2.335722	4.289875	0.085654
35	1	0	-2.414916	3.574114	0.899302
36	6	0	-4.537438	0.956244	-3.911881
37	1	0	-5.090864	1.859939	-3.622823
38	1	0	-5.240725	0.114390	-3.951692
39	1	0	-4.157154	1.121406	-4.932149
40	6	0	-1.826074	-2.325987	-1.142668
41	1	0	-1.644755	-2.218135	-0.064175
42	6	0	-0.570371	-3.003699	-1.684167
43	6	0	-0.023418	-2.698839	-2.940094
44	1	0	-0.468218	-1.896708	-3.525224
45	6	0	1.096264	-3.393420	-3.422624
46	1	0	1.506483	-3.141467	-4.399256
47	6	0	1.689539	-4.404933	-2.657754
48	1	0	2.558103	-4.942491	-3.032833
49	6	0	1.143513	-4.723578	-1.399814
50	1	0	1.591483	-5.510555	-0.795442
51	6	0	0.027707	-4.029800	-0.926452
52	1	0	-0.396012	-4.270880	0.047131
53	6	0	-3.038850	-3.242301	-1.285095
54	6	0	-3.266839	-4.016682	-2.431937
55	1	0	-2.545136	-3.992520	-3.246814
56	6	0	-4.408065	-4.818160	-2.534868
57	1	0	-4.572537	-5.412893	-3.432450
58	6	0	-5.333931	-4.859268	-1.487034
59	1	0	-6.220675	-5.486518	-1.565028
60	6	0	-5.108984	-4.096172	-0.336205
61	1	0	-5.816298	-4.126758	0.491036
62	6	0	-3.969565	-3.294199	-0.236890
63	1	0	-3.795556	-2.708304	0.663039
64	6	0	0.288885	0.410091	1.465297
65	6	0	1.570296	0.623008	2.068615

66	6	0	1.686736	0.903542	3.432087
67	1	0	2.684203	1.013057	3.858828
68	6	0	0.571837	1.008650	4.266693
69	6	0	-0.680194	0.772996	3.688520
70	1	0	-1.551148	0.726440	4.341269
71	6	0	-0.849153	0.491032	2.329880
72	6	0	2.854816	0.371862	1.274693
73	1	0	2.518486	0.230580	0.228551
74	6	0	3.857666	1.519047	1.183078
75	6	0	4.955194	1.354022	0.318606
76	1	0	5.055710	0.412322	-0.213433
77	6	0	5.897966	2.364724	0.137532
78	1	0	6.739155	2.207342	-0.536849
79	6	0	5.751586	3.585312	0.807904
80	1	0	6.478945	4.382905	0.666425
81	6	0	4.650929	3.773724	1.646964
82	1	0	4.513240	4.724808	2.159078
83	6	0	3.713473	2.749659	1.834148
84	1	0	2.855086	2.919350	2.477465
85	6	0	3.457099	-0.970333	1.709443
86	6	0	2.663386	-2.132365	1.606778
87	1	0	1.624839	-2.031212	1.284021
88	6	0	3.166523	-3.391065	1.938061
89	1	0	2.525919	-4.267668	1.845117
90	6	0	4.477637	-3.522656	2.415879
91	1	0	4.874614	-4.501182	2.680332
92	6	0	5.256411	-2.374086	2.574581
93	1	0	6.267542	-2.453275	2.971858
94	6	0	4.752643	-1.114591	2.226376
95	1	0	5.379170	-0.235561	2.352818
96	6	0	0.709732	1.329103	5.736798
97	1	0	-0.059014	0.812417	6.327737
98	1	0	0.595717	2.408596	5.925435
99	1	0	1.695969	1.031133	6.116898
100	6	0	-2.238483	0.073430	1.848117
101	1	0	-2.107160	-0.260626	0.817481
102	6	0	-3.290534	1.173788	1.758262
103	6	0	-3.508068	2.132510	2.758561
104	1	0	-2.869111	2.159584	3.635366
105	6	0	-4.528313	3.081164	2.630976
106	1	0	-4.675873	3.820586	3.417083
107	6	0	-5.346782	3.090184	1.497971

108	1	0	-6.131038	3.838032	1.393172
109	6	0	-5.135382	2.143586	0.492007
110	1	0	-5.750332	2.150248	-0.406339
111	6	0	-4.121045	1.193846	0.626483
112	1	0	-3.953922	0.466985	-0.165231
113	6	0	-2.726908	-1.187208	2.572131
114	6	0	-4.046470	-1.359136	3.008848
115	1	0	-4.753371	-0.538243	2.914816
116	6	0	-4.468141	-2.583206	3.545921
117	1	0	-5.498775	-2.696352	3.879969
118	6	0	-3.578306	-3.654050	3.648896
119	1	0	-3.909359	-4.607250	4.057800
120	6	0	-2.253821	-3.489068	3.223030
121	1	0	-1.548457	-4.315697	3.303089
122	6	0	-1.833637	-2.268187	2.696273
123	1	0	-0.805090	-2.135668	2.365270
124	6	0	5.552928	-2.554332	-1.118337
125	1	0	5.755061	-3.219752	-1.976631
126	1	0	5.212943	-3.156041	-0.267835
127	6	0	6.811740	-1.776447	-0.738603
128	1	0	6.613404	-1.124221	0.118090
129	1	0	7.183205	-1.171735	-1.575376
130	1	0	7.600368	-2.487764	-0.458843
131	6	0	4.592868	-0.893830	-2.612101
132	1	0	5.001618	-1.509463	-3.432672
133	1	0	5.307716	-0.086048	-2.396421
134	6	0	3.230983	-0.320031	-2.968584
135	1	0	2.779791	0.206746	-2.117005
136	1	0	2.544849	-1.112678	-3.298559
137	1	0	3.320007	0.407941	-3.780573

Final electronic energy: -2383.60832045 au
Final Gibbs free energy: -2059878.6 kcal mol^{-1}

Converged geometry, atomic coordinates, final electronic energy, and final Gibbs free energy of **1Na□THF$_{plan}$**:

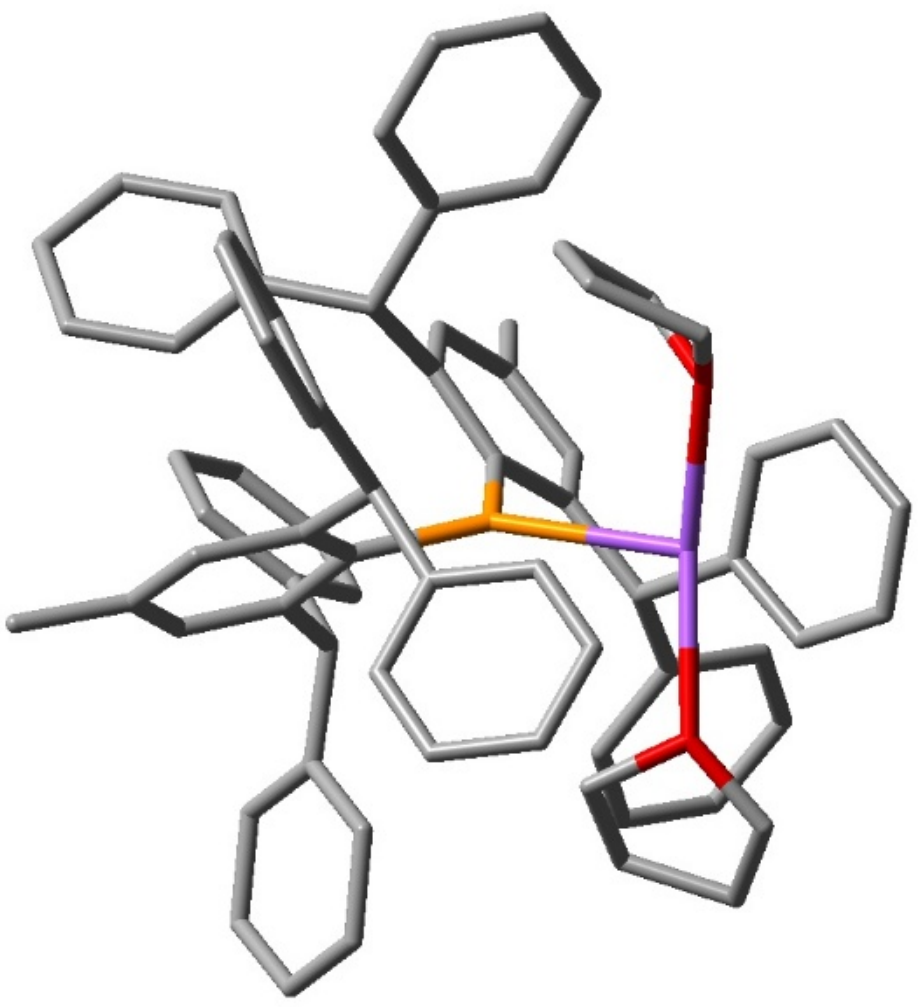

Center Number	Atomic Number	Atomic Type	Coordinates (Angstroms) X	Y	Z
1	15	0	0.044931	-0.524055	-0.109234
2	8	0	-1.189055	-4.372612	0.588517
3	8	0	2.280103	-3.229979	-2.252825
4	6	0	0.469314	0.353394	1.450486
5	6	0	-0.404942	1.267108	2.110413
6	6	0	-0.008461	1.876566	3.304468
7	1	0	-0.708183	2.551192	3.798122
8	6	0	1.235155	1.633467	3.897839
9	6	0	2.070876	0.696542	3.282598
10	1	0	3.039795	0.469553	3.726893
11	6	0	1.704184	0.052874	2.096777
12	6	0	-1.827625	1.537109	1.613796
13	1	0	-2.012867	0.811345	0.814864
14	6	0	-2.838463	1.172056	2.706903
15	6	0	-3.710244	2.091291	3.301821
16	1	0	-3.686306	3.131399	2.984940
17	6	0	-4.617427	1.679002	4.286904
18	1	0	-5.287283	2.408587	4.740419
19	6	0	-4.666933	0.341719	4.687236
20	1	0	-5.374514	0.022186	5.450831
21	6	0	-3.795750	-0.584573	4.098872
22	1	0	-3.820856	-1.629413	4.407233
23	6	0	-2.891196	-0.170106	3.122226
24	1	0	-2.202217	-0.880908	2.669857

25	6	0	1.653101	2.360960	5.154946
26	1	0	1.972669	3.390986	4.931509
27	1	0	2.492516	1.853080	5.647113
28	1	0	0.820839	2.429056	5.869293
29	6	0	2.598594	-1.033762	1.514525
30	1	0	2.372086	-1.033195	0.431133
31	6	0	2.184357	-2.414327	2.012660
32	6	0	2.124691	-3.482443	1.106670
33	1	0	2.355285	-3.286033	0.063507
34	6	0	1.778920	-4.770040	1.524834
35	1	0	1.731764	-5.584023	0.802366
36	6	0	1.482787	-5.009830	2.869669
37	1	0	1.209394	-6.009834	3.202429
38	6	0	1.532133	-3.950501	3.782819
39	1	0	1.297739	-4.126203	4.831918
40	6	0	1.876853	-2.663747	3.357204
41	1	0	1.897334	-1.839756	4.066786
42	6	0	4.088771	-0.743637	1.625406
43	6	0	5.012859	-1.622587	2.204170
44	1	0	4.663196	-2.551308	2.650263
45	6	0	6.380335	-1.317087	2.211678
46	1	0	7.084146	-2.011177	2.669334
47	6	0	6.841728	-0.130509	1.637448
48	1	0	7.904844	0.104827	1.642078
49	6	0	5.924558	0.756238	1.057986
50	1	0	6.270340	1.686361	0.608911
51	6	0	4.564592	0.451222	1.054978
52	1	0	3.852314	1.145440	0.613549
53	6	0	-0.660319	0.785175	-1.200960
54	6	0	0.041748	1.977401	-1.533723
55	6	0	-0.569056	2.940709	-2.339702
56	1	0	-0.032120	3.858123	-2.567614
57	6	0	-1.837966	2.755564	-2.895359
58	6	0	-2.494523	1.549511	-2.638793
59	1	0	-3.478837	1.379475	-3.072315
60	6	0	-1.938728	0.582513	-1.798822
61	6	0	1.528309	2.149433	-1.188833
62	1	0	1.769731	1.361757	-0.465762
63	6	0	2.434502	1.867715	-2.398531
64	6	0	1.959241	1.310916	-3.594483
65	1	0	0.895268	1.121942	-3.708332
66	6	0	2.832883	1.011466	-4.646757

67	1	0	2.436879	0.587423	-5.568976
68	6	0	4.201394	1.261464	-4.522750
69	1	0	4.880291	1.033850	-5.343206
70	6	0	4.689819	1.812704	-3.332155
71	1	0	5.754706	2.010379	-3.217328
72	6	0	3.815420	2.109029	-2.286318
73	1	0	4.201836	2.546563	-1.368311
74	6	0	-2.475656	3.835813	-3.734749
75	1	0	-2.809874	4.666306	-3.095737
76	1	0	-3.348465	3.453282	-4.279580
77	1	0	-1.763897	4.248159	-4.463904
78	6	0	-2.677658	-0.724727	-1.526365
79	1	0	-2.397537	-0.982885	-0.487257
80	6	0	-2.134648	-1.904639	-2.335786
81	6	0	-2.628519	-3.194107	-2.072682
82	1	0	-3.439397	-3.308977	-1.358581
83	6	0	-2.083821	-4.318774	-2.697140
84	1	0	-2.481648	-5.306938	-2.473914
85	6	0	-1.021729	-4.178532	-3.600426
86	1	0	-0.584286	-5.053421	-4.077391
87	6	0	-0.531415	-2.899555	-3.884158
88	1	0	0.299849	-2.776013	-4.573866
89	6	0	-1.084981	-1.775413	-3.259272
90	1	0	-0.679290	-0.788547	-3.462332
91	6	0	-2.142712	-5.442554	0.662823
92	1	0	-1.802010	-6.175560	1.413265
93	1	0	-2.176952	-5.923947	-0.320324
94	6	0	-3.483031	-4.795192	1.094771
95	1	0	-3.975968	-5.399141	1.865013
96	1	0	-4.173236	-4.702443	0.248752
97	6	0	-3.061638	-3.396866	1.631162
98	1	0	-3.377466	-2.605522	0.942533
99	1	0	-3.490454	-3.175556	2.613397
100	6	0	-1.529112	-3.472535	1.671324
101	1	0	-1.033433	-2.517195	1.465161
102	1	0	-1.158246	-3.896625	2.617168
103	6	0	3.582066	-3.841969	-2.146260
104	1	0	3.916895	-4.157685	-3.149237
105	1	0	3.478438	-4.725025	-1.504356
106	6	0	4.497212	-2.750160	-1.569441
107	1	0	4.484028	-2.775301	-0.474634
108	1	0	5.534698	-2.875323	-1.899643

109	6	0	3.860768	-1.431546	-2.087372
110	1	0	3.709001	-0.724536	-1.268201
111	1	0	4.480127	-0.933555	-2.837798
112	6	0	2.514183	-1.864347	-2.698715
113	1	0	1.675012	-1.258680	-2.340828
114	1	0	2.549611	-1.850239	-3.798728
115	6	0	-2.068812	2.911309	0.995147
116	6	0	-3.215140	3.090476	0.203410
117	1	0	-3.883824	2.249163	0.038180
118	6	0	-3.499275	4.323668	-0.381561
119	1	0	-4.390919	4.434461	-0.997099
120	6	0	-2.629188	5.405161	-0.200248
121	1	0	-2.841018	6.365003	-0.669786
122	6	0	-1.484913	5.238600	0.582683
123	1	0	-0.785211	6.060648	0.722323
124	6	0	-1.217768	4.004896	1.185325
125	1	0	-0.321786	3.892489	1.783258
126	6	0	1.869333	3.472327	-0.510252
127	6	0	2.029381	4.664112	-1.234663
128	1	0	1.950337	4.645238	-2.319582
129	6	0	2.319221	5.865272	-0.583537
130	1	0	2.430453	6.780797	-1.163064
131	6	0	2.480250	5.891191	0.806811
132	1	0	2.712588	6.826273	1.314452
133	6	0	2.359783	4.703204	1.533053
134	1	0	2.499562	4.701698	2.613071
135	6	0	2.057654	3.506065	0.877622
136	1	0	1.951549	2.590479	1.450487
137	6	0	-4.190826	-0.610729	-1.552905
138	6	0	-4.856835	-0.237408	-0.374653
139	1	0	-4.280271	-0.069729	0.534157
140	6	0	-6.244697	-0.078316	-0.356738
141	1	0	-6.741008	0.212277	0.567830
142	6	0	-6.988919	-0.295255	-1.521796
143	1	0	-8.070810	-0.173198	-1.511637
144	6	0	-6.335778	-0.676440	-2.698869
145	1	0	-6.909058	-0.849635	-3.608629
146	6	0	-4.946157	-0.835010	-2.712407
147	1	0	-4.439385	-1.136421	-3.627852
148	11	0	0.106226	-3.211127	-1.090663

Final electronic energy: -5314.78746373 au
Final Gibbs free energy: -2204890.1 kcal mol^{-1}
